# RER1 regulates lipid metabolism in monocytes and macrophages

**DOI:** 10.1007/s00018-025-05817-3

**Published:** 2025-08-13

**Authors:** Yanxia Liu, Sandra Theil, Mohamed H. Yaghmour, Anja Kerksiek, Peng Chen, Ingo G.H. Schmidt-Wolf, Rebecca Barker, Eva Bartok, Dieter Lütjohann, Christoph Thiele, Jochen Walter

**Affiliations:** 1 Center of Neurology, University Hospital Bonn, Bonn, Germany; 2 LIMES Life and Medical Sciences Institute, University of Bonn, Bonn, Germany; 3 Institute of Clinical Chemistry and Clinical Pharmacology, University Hospital Bonn, Bonn, Germany; 4 Department of Integrated Oncology, Center for Integrated Oncology (CIO), University Hospital Bonn, Bonn, Germany; 5 Institute of Experimental Hematology and Transfusion Medicine, University Hospital Bonn, Bonn, Germany

## Abstract

Retention in endoplasmic reticulum sorting receptor 1 (RER1) mediates the retention and retrieval of select cargo proteins, and thereby regulates protein transport in the secretory pathway and assembly of distinct protein complexes. Recently, RER1 was implicated in the assembly and subcellular transport of the TREM2-DAP12 immune receptor complex, and its function in intracellular signaling and phagocytosis. However, the role of RER1 in the regulation of immune cell metabolism remained unknown. Here, we demonstrate an important role of RER1 in the lipid metabolism of monocytic and macrophage-like differentiated THP-1 cells. The deficiency of RER1 resulted in the accumulation of lipid droplets (LDs) in both monocytes and macrophage-like cells. Comprehensive mass spectrometry analyses revealed complex changes in the cellular lipid metabolism and the composition of LDs. RNA sequencing revealed an important role of RER1 in the regulation of genes related to lipid metabolism. Further, western immunoblotting confirmed an important role of RER1 in the expression of select key proteins involved in cellular lipid metabolism. The combined data indicate that RER1 plays an essential role in lipid metabolism in monocytes and macrophages.

**Supplementary Information:**

The online version contains supplementary material available at 10.1007/s00018-025-05817-3.

## Introduction

Retention in endoplasmic reticulum sorting receptor 1 (RER1) is a sorting receptor localized in the endoplasmic reticulum-Golgi intermediate compartment (ERGIC) and cis-Golgi. RER1 can retrieve select proteins from these compartments to the ER to control proper subcellular localization, assembly of protein complexes or degradation [1–3]. Notably, RER1 dysfunction is involved in several diseases, including Charcot-Marie-Tooth (CMT) disease, Parkinson’s disease and pancreatic cancer [4–8]. However, the underlying mechanisms are not well understood but might involve impaired intracellular trafficking and metabolism of specific cargo proteins [4, 6, 7, 9]. Client proteins of RER1 in mammalian cells include the presenilin enhancer 2 (PEN2), nicastrin, α1-subunits of skeletal muscle nicotinic acetylcholine receptor (nAChR), and the peripheral myelin protein 22 (PMP22) [4, 8–14]. Recently, we identified the immune co-receptor DAP12 as a client of RER1. The deficiency of RER1 strongly reduces the levels of TREM2-DAP12 immunocomplexes and TREM2-DAP12 dependent intracellular signaling in differentiated macrophage-like THP-1 cells [15], thereby linking RER1 to immune cell function.

The transition of immune cells to different functional states critically involves lipid metabolic pathways [16–19]. Lipid metabolism also plays important roles in the regulation of macrophages and microglia during inflammatory processes [20–23]. Lipid droplets (LDs) are dynamic cytoplasmic organelles for the storage of fatty acids (FAs) in the form of neutral lipids like triacylglycerols (TAGs) and cholesterol esters (CEs) and are important for cellular energy metabolism. LD are surrounded by a phospholipid monolayer composed of phospholipids and associated proteins, including perilipin proteins [24–29]. Besides lipid storage for energy metabolism, LDs can also store other lipophilic molecules, like vitamins and certain signaling precursors. In addition, LDs play a vital role in the response to endoplasmic reticulum (ER) and oxidative stress, and also assist the maturation, storage, and turnover of proteins [30].

Here, we analyzed the role of RER1 in the lipid metabolism of monocytes and macrophage-like cells. Immunocytochemistry and comprehensive mass spectrometry showed that the deficiency of RER1 leads to the accumulation of lipid droplets and complex changes in cholesterol and glycerolipid metabolisms. RNA sequencing and western immunoblotting also support an important role of RER1 in the regulation of genes associated with lipid metabolism in monocytes and macrophage-like cells. Thus, the combined results demonstrate that RER1 exerts important functions in the lipid metabolism of immune cells.

## Materials and methods

**Table:** 

Reagent/resource	Reference or source	Identifier or catalog number	
**Chemicals/Reagents**			
Phorbol 12-myristate 13-acetate (PMA)	Sigma-Aldrich	Cat# P8139	
β-Mercaptoethanol	Sigma-Aldrich	Cat# M7522	
n-dodecyl-β-d-Maltoside	Sigma-Aldrich	Cat# D4641	
Protease inhibitor cocktail	Sigma-Aldrich	Cat# 04693116001	
Fetal Bovine Serum	PAN-Biotech	Cat# P30-3306	
Penicillin/streptomycin (P/S)	Gibco	Cat# 15140-122	
Paraformaldehyde (PFA)	Sigma-Aldrich	Cat# 16005-1KG-R	
Triton X-100	Carl Roth	Cat# 3051.2	
Tween^®^ 20	Sigma-Aldrich	Cat# P1379	
Immu-Mount™	Fisher Scientific	Cat# 9,990,402	
Ibidi mounting medium	Ibidi	Cat# 50,011	
LD540 dye	Lab Prof. Christoph Thiele (U. Bonn)	[31]	
**Antibodies**			
**Primary antibodies**			
Perilipin 2	Progen	Cat# 610,102	
CYP51A1	Proteintech	Cat#13431-1-AP	
LDLR	Abcam	Cat#ab52818	
LRP1	Abcam	Cat#ab92544	
NPC1	Abcam	Cat#ab36983	
ABCA1	Novus Biologicals	Cat#NB400-105	
APOE	Abcam	Ab1906	
**Secondary antibodies**			
IRDye800CW donkey anti mouse	Li-COR	Cat# 926-32212	
IRDye680CW donkey anti rabbit	Li-COR	Cat# 926-68073	
Alexa Fluor 546-conjugated anti-rabbit	Invitrogen	Cat# A10040	

### Cell culture

THP-1 cells were cultured in T75 flasks in an incubator with 5% CO_2_ at 37 °C with RPMI 1640 medium (Gibco, USA) containing 10% FBS, 1% P/S, 0.05 µM β-mercaptoethanol (Sigma-Aldrich, M7522, Germany). Cells were split before the cell concentration reached 1,000,000 cells/mL. The differentiation of THP-1 cells to macrophage-like cells was performed by incubating monocytes with 5 ng/mL phorbol 12-myristate 13-acetate (PMA, Sigma-Aldrich, P8139, Germany) for 48 h and recovering them in normal RPMI medium without PMA for one day. The RER1 knock out (RER1 ko) cells used in this study were described previously [15].

### Detection of LD540 florescence by flow cytometry

Undifferentiated cells were washed with PBS for three times after collecting and counting, and incubated with 0.5 µg/mL lipophilic dye LD540 in 1x DPBS for 20 min in a cell incubator with 5% CO_2_ at 37 ℃. Afterwards, the cells were washed twice with DPBS and resuspended in the same solution. All samples were analyzed by FACSAria II flow cytometer (BD Biosciences, San Jose, USA) by exciting with a 488 nm laser and detecting the fluorescence emission at 530 nm. FlowJo (BD Biosciences, San Jose, USA) was used for data processing.

### Preparation of cellular membrane fractions

Cells were washed with ice-cold PBS and harvested from culture dishes in hypotonic buffer (10 mM Tris, 1 mM EDTA, 1 mM EGTA, pH 7.4) supplemented with protease inhibitor cocktail (Sigma-Aldrich, 04693116001, Germany) by scraping. After 10 min incubation on ice, syringes with 0.6 mm cannula were used to mechanically homogenize the cells, followed by 10 min centrifugation at 200 g (4 °C). Resulting supernatants were centrifuged for 1 h at 16,000 g (4 °C). Proteins were extracted from the pellets with lysis buffer (1% n-dodecyl-β-d-maltoside (Sigma-Aldrich, D4641, Germany), 50 mM Tris–HCl (pH 8.0), 150 mM NaCl, 1 mM EDTA, 1.5 mM MgCl_2_, 10% glycerol) completed with protease inhibitor cocktail for 20 min on ice. Extracts were centrifuged for 10 min at 16,000 g (4 °C), and the protein concentration in the supernatants was determined by Bradford Assay.

### Sodium dodecyl-sulfate polyacrylamide gel electrophoresis (SDS-PAGE) and western Immunoblotting

Samples were heated at 95 °C for 5 min with SDS loading buffer (250 mM Tris–HCl (pH 6.8), 10% SDS, 500 mM DTT, 43% glycerol, 1.2 µM bromphenolblau) before separating proteins on pre-casted NuPAGE Novex Bis-Tris Gels 4–12% (Invitrogen, USA), using NuPAGE running chambers and NuPAGE MES SDS Running Buffer (Invitrogen, NP0002, USA) at 150 V. Proteins were transferred to nitrocellulose membranes for 105 min at 400 mA using a wet blot chamber system. Proteins were stained with Ponceau S solution at room temperature (RT) and membranes imaged with a Chemidoc XRS Imager (Bio-Rad, USA). Nitrocellulose membranes were blocked in Tris-Buffered Saline containing 0.1% Tween^®^ 20 Detergent (TBST) and 5% milk powder at RT, washed three times in TBST, and then incubated with primary antibodies diluted in TBST at 4 °C for overnight. Membranes were washed three times (5 min each), and incubated with fluorescently labeled secondary antibodies diluted in TBST for 1 h at RT. Membranes were finally washed three times with TBST (5 min each). All incubations were completed with constant overhead, horizontal or orbital shaking. Fluorescence signals were detected using an Odyssey^®^ CLx Imager (Li-COR Biosciences, Germany).

### Immunocytochemistry

THP-1 monocytes were seeded in ibidi dishes (ibidi, Gräfelfing, Germany), differentiated with 5 ng/mL PMA for 48 h, and recovered in normal RPMI medium for 1 day. The differentiated macrophage-like cells were fixed with PBS containing 4% paraformaldehyde (PFA, Sigma-Aldrich, Steinheim, Germany) for 20 min at room temperature (RT) and then washed with PBS three times. Cells were permeabilized with 0.1% Triton X-100 (Carl Roth, Karlsruhe, Germany) in PBS for 15 min. The blocking solution (3% bovine serum albumin (BSA) in PBST (0.1% Tween^®^ 20)) was applied to the cells for 1 h at RT. Afterwards, cells were incubated with PBST containing primary antibodies and 1% BSA for 1 h at RT followed by three washes with PBST. Cells were then incubated with secondary antibody solution (PBST, 1% BSA) for 1 h at RT. Finally, cells were washed three times with PBST and two times with PBS before addition of immune mounting medium supplemented with DAPI (ibidi, Gräfelfing, Germany).

For the visualization of LDs in differentiated THP-1 cells, cells were incubated with 0.5 µg/mL LD540 dye for 30 min after fixation with 4% PFA. Subsequently, cells were washed with PBS for three times before ibidi mounting medium was added to the dishes.

Images were taken with a Zeiss microscope (AxioVert 200) supplied with a Zeiss ApoTome using a 63/1.4 objective and DsRed and DAPI fluorescence filter sets. Images were processed with ImageJ (NIH, USA). For quantitative image analysis, images were randomly captured with identical camera settings within individual experiments. Analysis of LDs number and volume was done with the Automatic Lipid Droplet Quantification (ALDQ) plugin of Fiji ImageJ according to a previous publication [32]. A total number of at least 424 cells was included for each experimental condition in ALDQ quantification. Analysis of perilipin 2 intensity was performed in ImageJ and at least 408 cells for each experimental condition.

### Quantification of cholesterol and non-cholesterol sterols in THP-1 cells

THP-1 undifferentiated and differentiated cells were harvested and washed three times with PBS and stored at − 80 °C until analysis. Per sample, 3.5 mio cells were dried in a speedvac concentrator (12 mbar; SpeedVac DNA 130–230 Vacuum Concentrator, Thermo Scientific, Darmstadt, Germany) and weighed. Cholesterol and non-cholesterol sterols were extracted using chloroform/methanol (2:1; vol/vol)). After alkaline hydrolysis, the concentrations of silylated cholesterol and non-cholesterol sterols were measured with gas chromatography-flame ionization detection (GC-FID) and GC-mass spectrometry selected ion monitoring (GC-MS-SIM), respectively, as previously described [33]. The degree of esterification for cholesterol was calculated from the quantification of total cholesterol content (after alkaline hydrolysis) and the free cholesterol content (extraction without alkaline hydrolysis) using GC-MS-SIM.

### Lipid extraction

THP-1 undifferentiated cells were collected and washed three times with PBS. THP-1 differentiated cells were washed three times with PBS and collected by scraping. The cell pellets (500,000 cells per sample) were stored at − 80 °C until analysis. Lipid extraction was performed as previously described [34]. Briefly, 500 µL methanol: chloroform 5/1 containing 250 pmol PE 31:1(d7), 472 pmol PC 33:1(d7), 98 pmol PS 31:1, 56 pmol PA 31:1, 51 pmol PG 28:0, 39 pmol LPA 17:0, 35 pmol LPC 17:1, 38 pmol LPE 17:1, 32 pmol Cer 17:0, 241 pmol SM 18:1(d9), 55 pmol GlcCer 12:0, 339.7 pmol TG 50:1-D4, 111 pmol CE 18:1(d6), 64 pmol DG 31:1 and 103 pmol MG 17:1 as internal standards were quickly added to the samples, followed by sonication for 30 s in a bath sonicator. Subsequently, the samples were centrifuged at 20,000 g for 2 min and the supernatants transferred into new tubes for further process. 300 µl chloroform and 700 µl 1% acetic acid in water were added to the samples to induce phase separation. The samples were shaken manually for 5 s and centrifuged at 20,000 g for 2 min. The upper phase was carefully removed and discarded. The entire lower phase was transferred into a new tube and evaporated in the centrifuge at 45 °C for 20 min. 1 mL spray buffer (2-propanol/methanol/water 8/5/1 + 10 mM ammonium acetate) was added and samples sonicated for 5 min.

### Tandem mass spectrometry

Mass spectra were recorded on a Thermo Q-Exactive Plus spectrometer equipped with a standard HESI ion source using direct injection from a Thermo Dionex AS-AP autosampler driven by an AXP-MS pump under the control of Xcalibur software. MS1 spectra (resolution 280,000) were recorded in 100 *m/z* windows from 250 to 1,200 *m/z* (positive mode) followed by recording MS/MS spectra (resolution 70,000) by data independent acquisition in 1 *m/z* windows from 250 to 1,200 *m/z* (positive mode). The raw data were converted to.mzML files using MSConvert and analyzed with LipidXplorer software. For further analysis, absolute amounts were calculated using internal standard intensities, followed by the calculation of the molar fraction (‰) for the identified lipids.

### RNA sequencing

Total RNA was extracted from THP-1 cells by RNeasy^®^ Mini Kit (QIAGEN, Hilden, Germany) according to the manufacturer’s instructions and genomic DNA was removed by using RNase-Free DNase (QIAGEN, Hilden, Germany). Transcriptome library construction was performed by QuantSeq 3’-mRNA Library Prep for Illumina (Lexogen, Vienna, Austria). Single-end 100-base reads were generated by NovaSeq 6000 sequencing system.

Raw reads were filtered by using SOAPnuke (https://github.com/BGI-flexlab/SOAPnuke) [35] and mapped to human reference genome (Homo_sapiens_NCBI_GCF_000001405.40_GRCh38.p14) by Hierarchical Indexing for Spliced Alignment of Transcripts (HISAT, http://www.ccb.jhu.edu/software/hisat) [36] and aligned to the reference genes by using Bowtie2. The matched reads were calculated as transcript per million (TPM). DESeq2 was used to identify differentially expressed genes (DEGs) by setting adjust *p* value (Qvalue) < 0.05 and|log2FoldChange| ≥ 1 and presented by heatmaps and volcano plots. Kyoto Encyclopedia of Genes and Genomes (KEGG) function enrichment analysis was performed to identify the associated biochemical and signal transduction pathways. The functional enrichment analysis of Gene Ontology (GO) was performed to explore the potential roles of the differentially expressed mRNAs. Both enrichment analyses were performed by using the online Dr. Tom software (https://biosys.bgi.com), in which Qvalue < 0.05 was used as significance threshold in GO and KEGG analysis.

### Statistics

Data were analyzed by two tailed Student’s t test using GraphPad Prism 9 (GraphPad Software, Inc, USA) and presented as mean ± SEM. Significance of statistics was considered when the p value is below 0.05 (*p < 0.05; **p < 0.01; ***p < 0.001; ****p < 0.0001).

## Results

### Accumulation of lipid droplets (LDs) upon RER1 deficiency

**Fig. 1:**
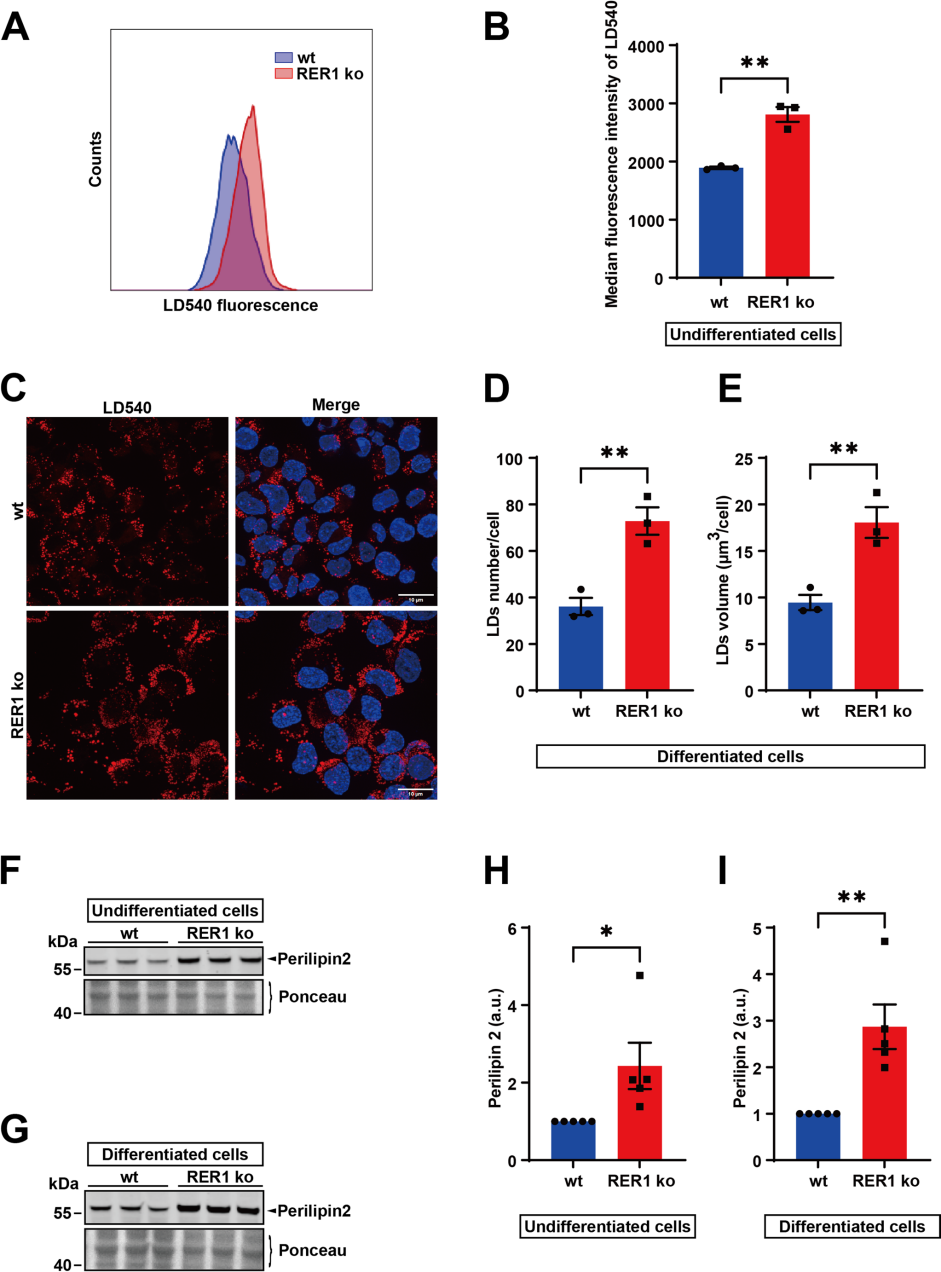
Accumulation of Lipid droplets (LDs) in RER1 ko cells. (**A**) Comparison of LDs in RER1 ko and wild type (wt) THP-1 undifferentiated cells by flow cytometry. LD540 dye was used for the staining of LDs. (**B**) Quantification of the median fluorescence intensity (MFI) for LD540 shown in (**A**). Data represent mean ± SEM of three independent experiments. Each data point represents the mean value of an individual experiment. Student’s t-test (unpaired, two-tailed). **p < 0.01. (**C**) Comparison of LDs in RER1 ko and wt THP-1 differentiated cells by LD540 staining. Representative images are shown. Cells were co-stained with LD540 (red) and DAPI (blue) to visualize LDs and nuclei, respectively. Scale bar = 10 μm. LDs numbers (**D**) and volume (**E**) per cell were quantified by automated LD quantification (ALDQ) method. Values represent mean ± SEM of three independent experiments and at least 120 cells per condition were included in each experiment. Each data point represents the mean value of an individual experiment. Student’s t-test (unpaired, two-tailed). **p < 0.01. (**F**)-(**G**) Detection of perilipin 2 in RER1 ko and wt THP-1 undifferentiated (**F**) and differentiated (**G**) cells. Cellular membranes were isolated and western immunoblotting was used for the detection of the indicated protein. (**H**)-(**I**) Quantification of perilipin 2 by western immunoblotting (as shown in A and B). Perilipin 2 was normalized to the full protein stained by Ponceau S. Data represent mean ± SEM of five independent experiments with one to three samples per experiment. Each data point represents the mean value of an individual experiment. Student’s t-test (unpaired, two-tailed). *p < 0.05, **p < 0.01. Similar data on the accumulation of lipid droplets and increased levels of perilipin 2 were obtained in two independent RER1 ko clones (Suppl Fig. 14)

To assess the role of RER1 in lipid homeostasis of an immune cell model, we first analyzed LDs in monocyte-like undifferentiated and macrophage-like differentiated THP-1 cells upon incubation with the lipophilic dye LD540. Flow cytometry analysis showed significantly increased LD540 fluorescence in RER1-deficient THP-1 cells as compared to wt cells (Fig. 1A and B). Similarly, fluorescence microscopy also revealed a substantially increased number and volume of LDs in RER1-deficient macrophage-like cells compared with wt cells (Fig. 1C-E).

In addition, the lipid droplet coat protein perilipin 2 was also significantly elevated in both RER1-deficient THP-1 monocytes (Fig. 1F and H) and macrophage-like cells (Fig. 1G and I, Suppl. Figure 1A and B). These observations strongly suggest that RER1 plays an important role in the metabolism of LDs.

### RER1 deficiency alters the levels of cholesterol esters (CEs) and triacylglycerols (TAGs)

**Fig. 2:**
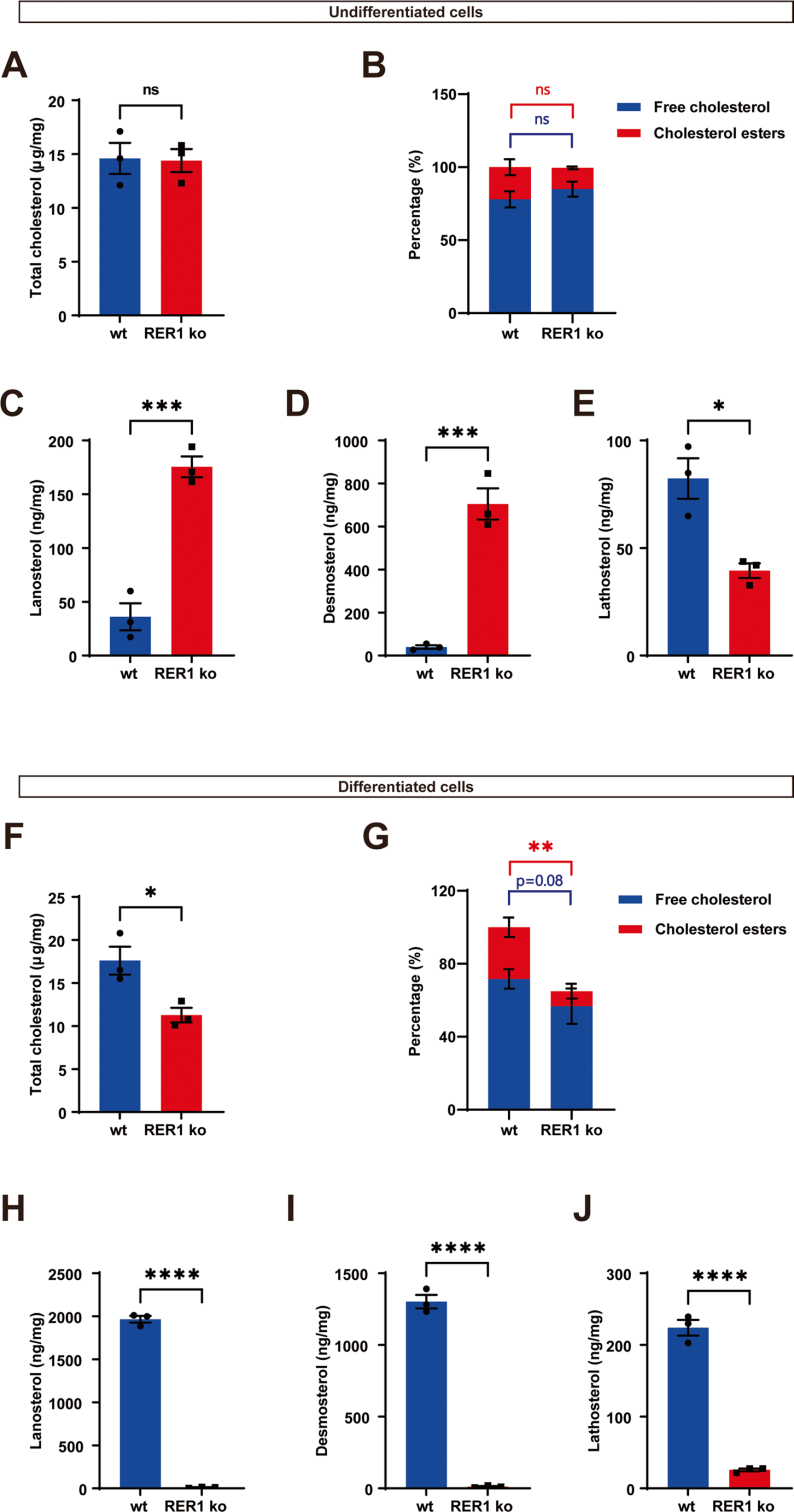
Content analysis of a panel of sterols in RER1 ko and wt THP-1 cells by GC-FID and GC-MS-SIM. Free cholesterol and cholesteryl esters are expressed as a percentage of total cholesterol. Total cholesterol (**A** and **F**, absolute amount), free cholesterol, cholesterol esters (**B** and **G**, % of total cholesterol) as well as cholesterol precursors (lanosterol (**C** and **H**), desmosterol (**D** and **I**), lathosterol (**E** and **J**)) were analyzed in THP-1 undifferentiated (**A**-**E**) and differentiated (**F**-**J**) cells. Values represent mean ± SEM of three independent experiments each performed with triplicate samples. Each data point represents the mean value of an individual experiment. Student’s t-test (unpaired, two-tailed). *p < 0.05, ***p < 0.001, ****p < 0.0001

Since LDs mainly contain CEs and TAGs, comprehensive GC-FID and GC-MS-SIM analyses were performed to detect cholesterol and several metabolites. The total cholesterol level was similar in wt and RER1-deficient THP-1 monocytes (Fig. 2A). The level of CEs was decreased to some extent, but differences were not significant (Fig. 2B). GC-MS-SIM analysis further showed that monocytic THP-1 cells lacking expression of RER1 contained strongly increased levels of the cholesterol precursors lanosterol and desmosterol (Fig. 2C and D), indicating upregulation of de novo cholesterol biosynthesis. Since the level of lathosterol was significantly decreased in RER1-deficient cells (Fig. 2E), these data also suggest that the upregulation of cholesterol biosynthesis in RER1-deficient monocytic THP-1 cells predominantly involves the Bloch pathway rather than the Kandutsch-Russell pathway (Suppl. Figure 2).

In macrophage-like differentiated cells, total cholesterol was significantly decreased in RER1-deficient as compared to the respective wt cells (Fig. 2F). Macrophage-like differentiated RER1-deficient cells also had a lower ratio of free cholesterol to esterified CEs as compared to the respective wt cells (Fig. 2G). In addition, macrophage-like differentiated THP-1 RER1-deficient cells had strongly reduced levels of cholesterol precursors lanosterol, lathosterol and desmosterol (Fig. 2H-J), indicating a downregulation of *de novo* sterol synthesis.

**Fig. 3:**
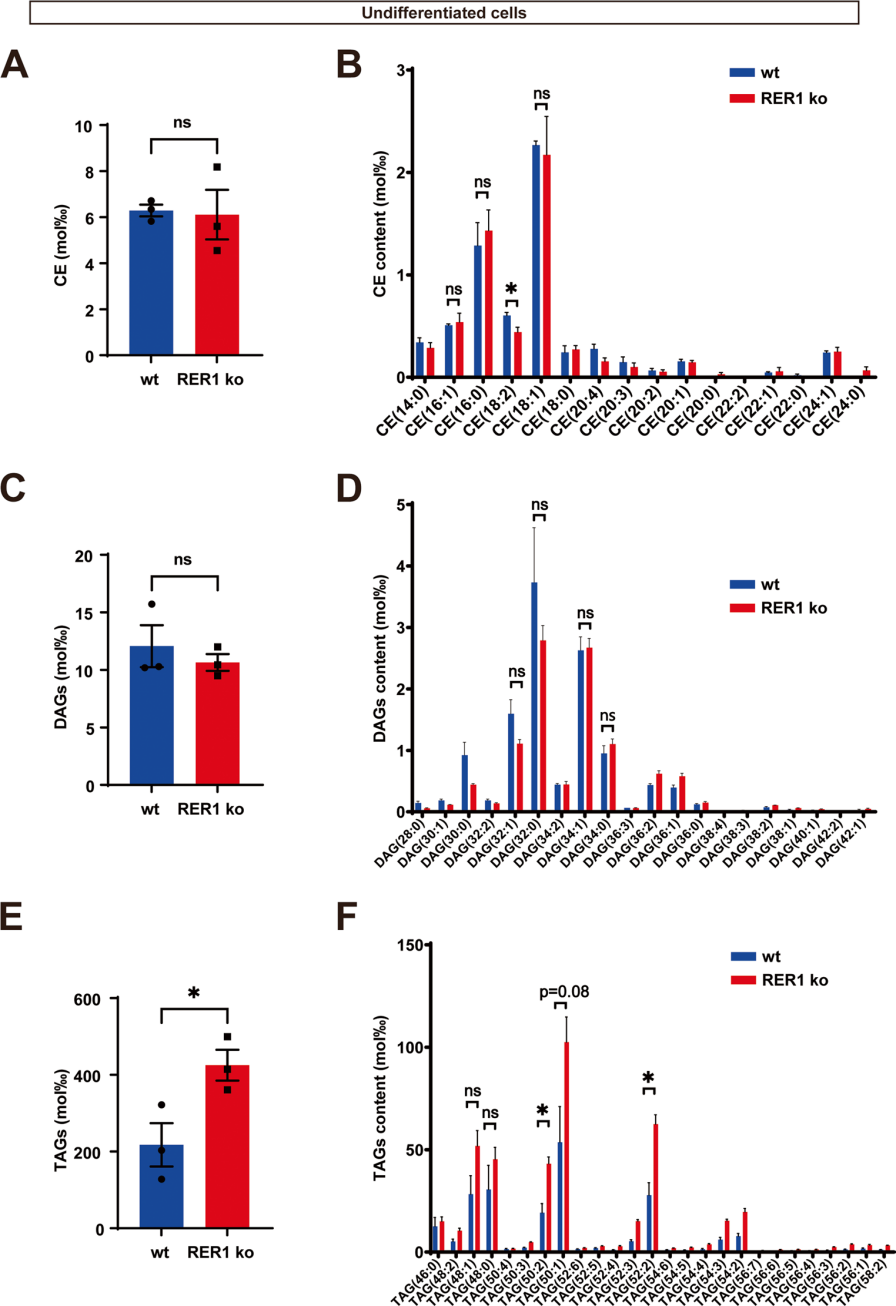
Analysis of cholesterol esters (CEs) species, diacylglycerol (DAGs) species and triacylglycerol (TAGs) species in THP-1 undifferentiated cells. Total CEs (**A**) and different species (**B**), total diacylglycerol (**C**) and different species (**D**), total triacylglycerol (**E**) and different species (**F**) were analyzed by tandem mass spectrometry. Values represent mean ± SEM of three independent experiments each performed with triplicate samples. Each data point represents the mean value of an individual replicate. Student’s t-test (unpaired, two-tailed). *p < 0.05

To further analyze the individual species of CEs, tandem mass spectrometry was performed. In line with GC-FID and GC-MS-SIM analysis (Fig. 2), the level of CEs was not changed in RER1-deficient THP-1 monocytes (Fig. 3A). CE (18:1) and CE (16:0) represented the most abundant CEs in THP-1 monocytes (Fig. 3B). To obtain more detailed insight into RER1 dependent alterations in the metabolism of LD associated lipids, MS analysis of acylglycerols was performed. Levels of diacylglycerol (DAG) were not significantly changed in undifferentiated RER1-deficient cells as compared to wt cells (Fig. 3C and D), while the TAGs were significantly increased (Fig. 3E and F).

**Fig. 4:**
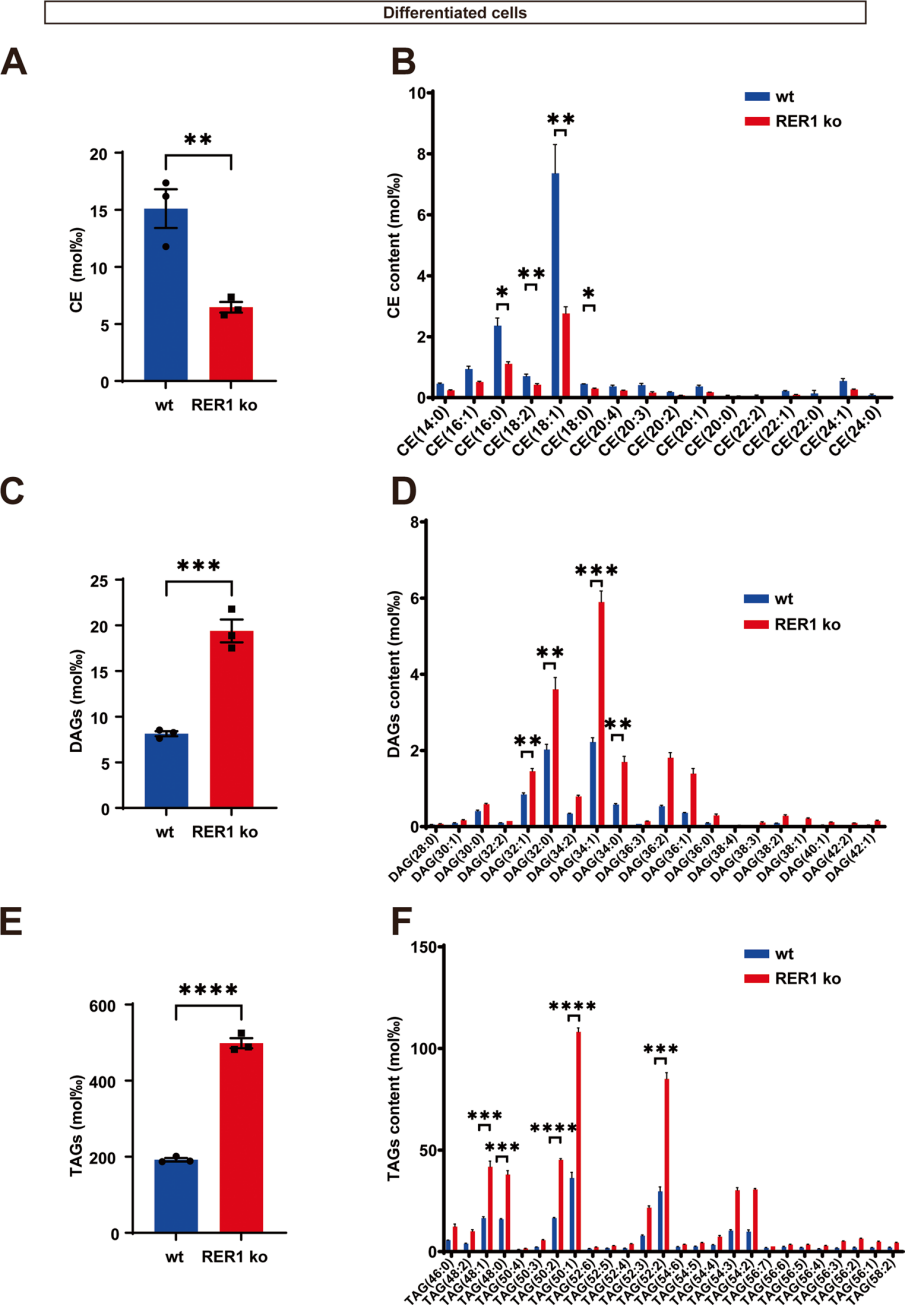
Analysis of cholesterol esters (CEs) species, diacylglycerol (DAGs) species and triacylglycerol (TAGs) species in THP-1 differentiated cells. Total CEs (**A**) and different species (**B**), total diacylglycerol (**C**) and different species (**D**), total triacylglycerol (**E**) and different species (**F**) were analyzed by tandem mass spectrometry. Values represent mean ± SEM of three independent experiments each performed with triplicate samples. Each data point represents the mean value of an individual replicate. Student’s t-test (unpaired, two-tailed). *p < 0.05, **p < 0.01, ***p < 0.001, ****p < 0.0001

In RER1-deficient macrophage-like cells, CEs were significantly reduced (Fig. 4A). Similar to THP-1 monocytes, CE (18:1) represented the most abundant CE species in THP-1 macrophage-like cells (Fig. 4B). This species was significantly reduced in RER1-deficient macrophage-like cells as compared with wt cells (Fig. 4B). Other species, such as CE (16:0), CE (16:1) and CE (18:2), were also significantly reduced in RER1-deficient cells (Fig. 4B). Thus, a decrease in these individual CE species contributes to the overall decreased CE content in RER1-deficient cells. MS analysis of acylglycerols showed that RER1-deficient macrophage-like differentiated THP-1 cells had increased levels of both DAGs and TAGs (Fig. 4C-D), the respective changes in DAGs and TAGs were detected for most of the TAG and DAG species. Together, these results indicate complex changes in the cellular lipid composition in RER1-deficient cells and support the contribution of increased acylglycerols to the elevated number of LDs. Additionally, RER1 deficiency had different effects on cholesterol synthesis in monocytic and macrophage-like THP-1 cells.

### Lipid metabolism related pathways are upregulated in RER1-deficient cells

**Fig. 5:**
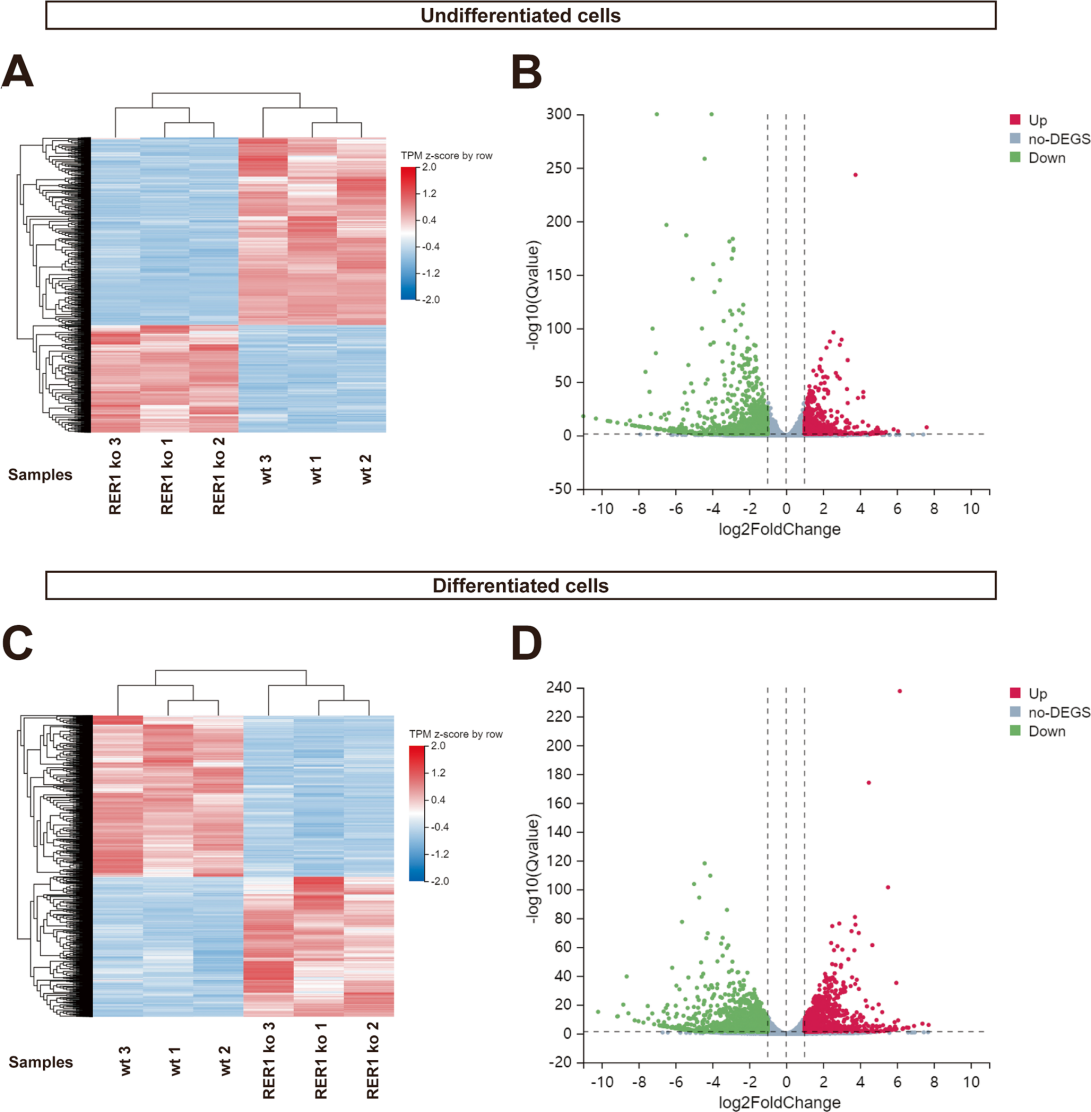
Differential mRNA expression associated with RER1 deficiency in THP-1 cells. (**A**) and (**C**) Heatmap representations of the differential cluster analysis. Here, wt 1, 2, 3 and RER1 ko 1, 2, 3 indicate different biological replicates from three individual experiments of the same clone. Differentially expressed genes (DEGs) were used to do the hierarchical clustering. (**B**) and (**D**) Volcano plot of up-regulation and down-regulation genes, red circles indicate up-regulated mRNAs, and green circles indicate down-regulated mRNAs. y-Axis corresponds to the log10 (Q value, adjust p value), and the x-axis displays the Log2FoldChange value

We also performed RNA sequencing (RNA seq) to assess the potential involvement of RER1 in transcriptional regulation of lipid metabolisms in both monocytes and macrophage-like cells. Hierarchical clustering analysis showed that a large number of genes was robustly changed in RER1-deficient monocytes as compared with wt monocytes (Fig. 5A). Using the criteria of Qvalue < 0.05 and|log2FoldChange| ≥ 1608 upregulated mRNAs and 1062 down-regulated mRNAs were found in the RER1-deficient monocytic THP-1 cells (Fig. 5B). Similarly, comprehensive transcriptomic changes were observed in macrophage-like RER1-deficient cells (Fig. 5C), with the upregulation of 1423 genes and the downregulation of 1635 genes as compared to the respective wt cells (Fig. 5D).

**Fig. 6:**
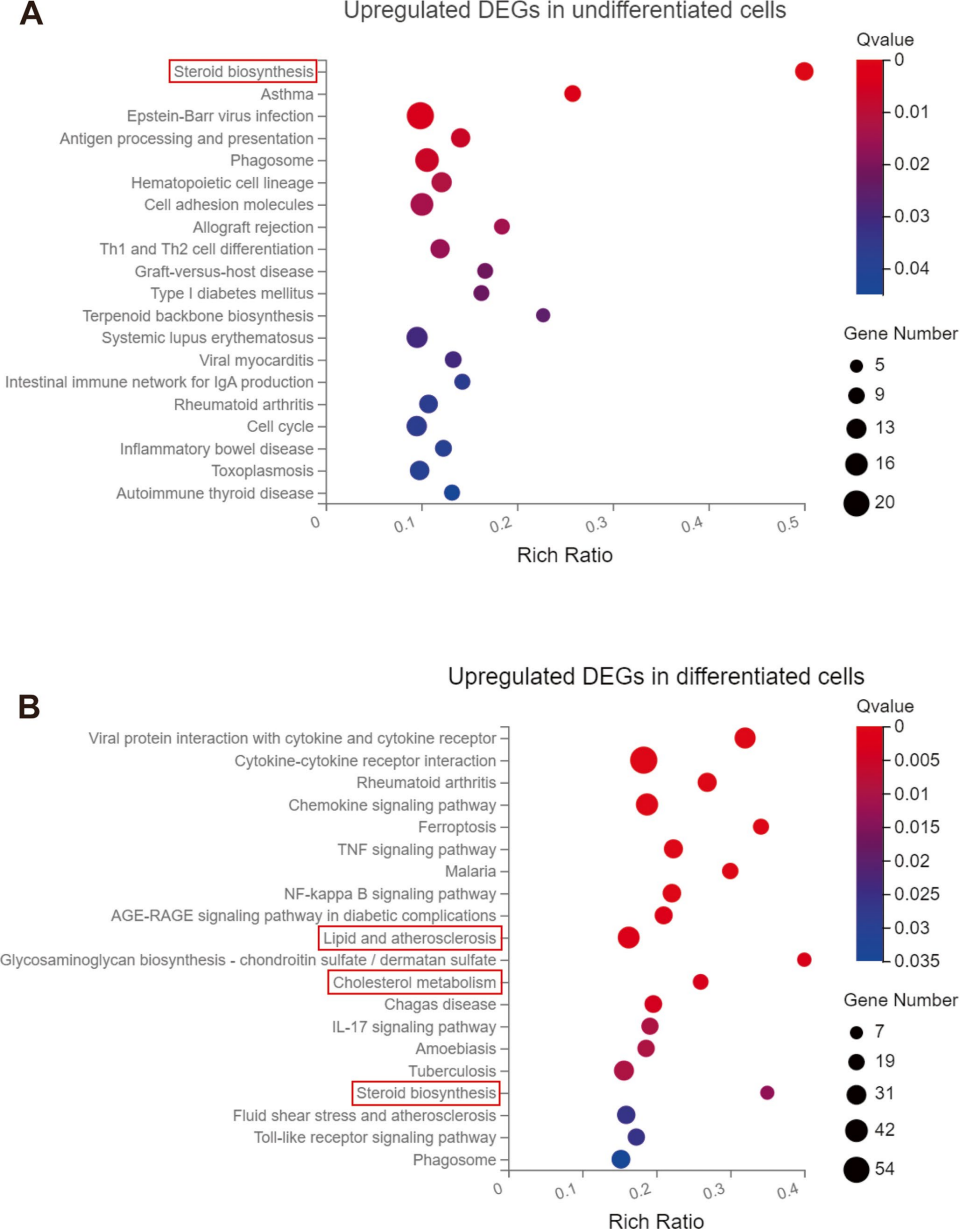
Kyoto Encyclopedia of Genes and Genomes (KEGG) enrichment analysis of differentially expressed genes (DEGs) in RER1-deficient versus wt THP-1 cells.(**A**) Upregulated KEGG pathways in undifferentiated THP-1 cells; (**B**) Upregulated KEGG pathways in differentiated THP-1 cells. Lipid metabolism-related pathways are highlighted (red boxes), and associated genes are listed in Supplementary Tables 1, 9, 10, and 11

KEGG function enrichment analysis revealed that genes related to lipid metabolism were significantly enriched in RER1-deficient cells as compared to wt cells (Fig. 6A and B). The steroid biosynthesis pathway was strongly upregulated in RER1-deficient monocytic THP-1 cells (Fig. 6A, Suppl. Figure 3). RER1-deficient macrophage-like differentiated THP-1 cells also showed significant upregulation of the pathways ‘lipid and atherosclerosis’, ‘cholesterol metabolism’ and ‘steroid biosynthesis’ as compared to the respective wt cells (Fig. 6B, Suppl. Figure 4). However, several immune related biological processes were more strongly affected by the deficiency of RER1 in the macrophage-like, differentiated cell type (Fig. 6B).

**Fig. 7:**
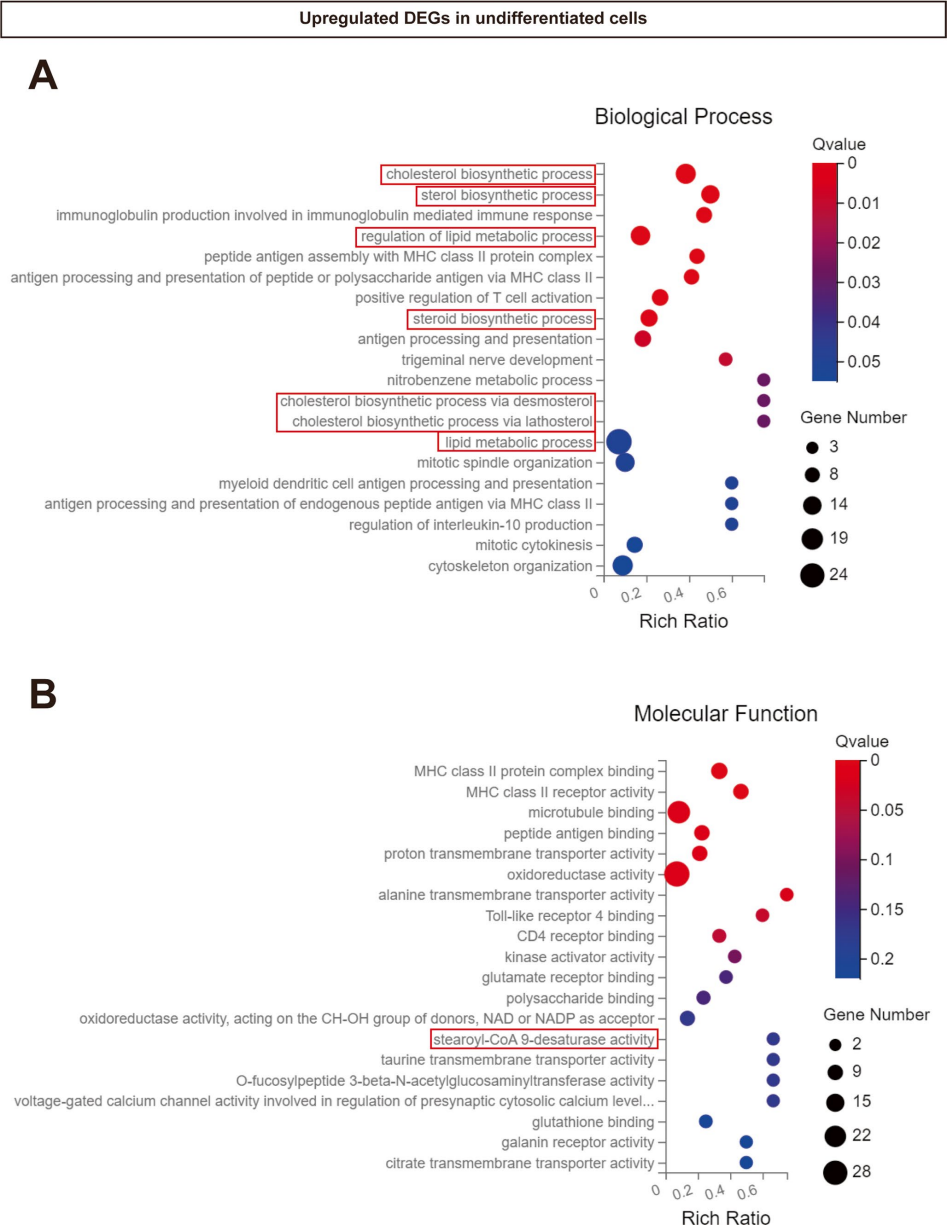
Gene Ontology (GO) enrichment analysis of differentially expressed genes (DEGs) in RER1-deficient versus wt undifferentiated THP-1 cells. (**A**) Upregulated GO terms for biological processes; (**B**) Upregulated GO terms for molecular functions. Lipid metabolism-related terms are highlighted (red boxes), with associated genes listed in Supplementary Tables 2–8

In the GO enrichment analysis, the upregulated DEGs in RER1-deficient THP-1 monocytes were partially attributed to ‘cholesterol biosynthetic process’, ‘sterol biosynthetic process’, ‘regulation of lipid metabolic process’, ‘steroid biosynthetic process’, ‘cholesterol biosynthetic process via desmosterol and lathosterol’, ‘lipid metabolic process’ (Fig. 7A, Suppl. Figure 5). Upregulated DEGs related to stearoyl-CoA 9-desaturase activity were observed in RER1-deficient THP-1 monocytes (Fig. 7B, Suppl. Figure 6). However, changes in immune related molecular functions were also identified (Fig. 7 and Suppl. Figure 7). Notably, there were no lipid metabolism related biological processes and molecular functions found in the TOP20 of upregulated GO enrichment analysis in RER1-deficient macrophage-like cells as compared to wt cells (Suppl. Figure 8).

**Fig. 8:**
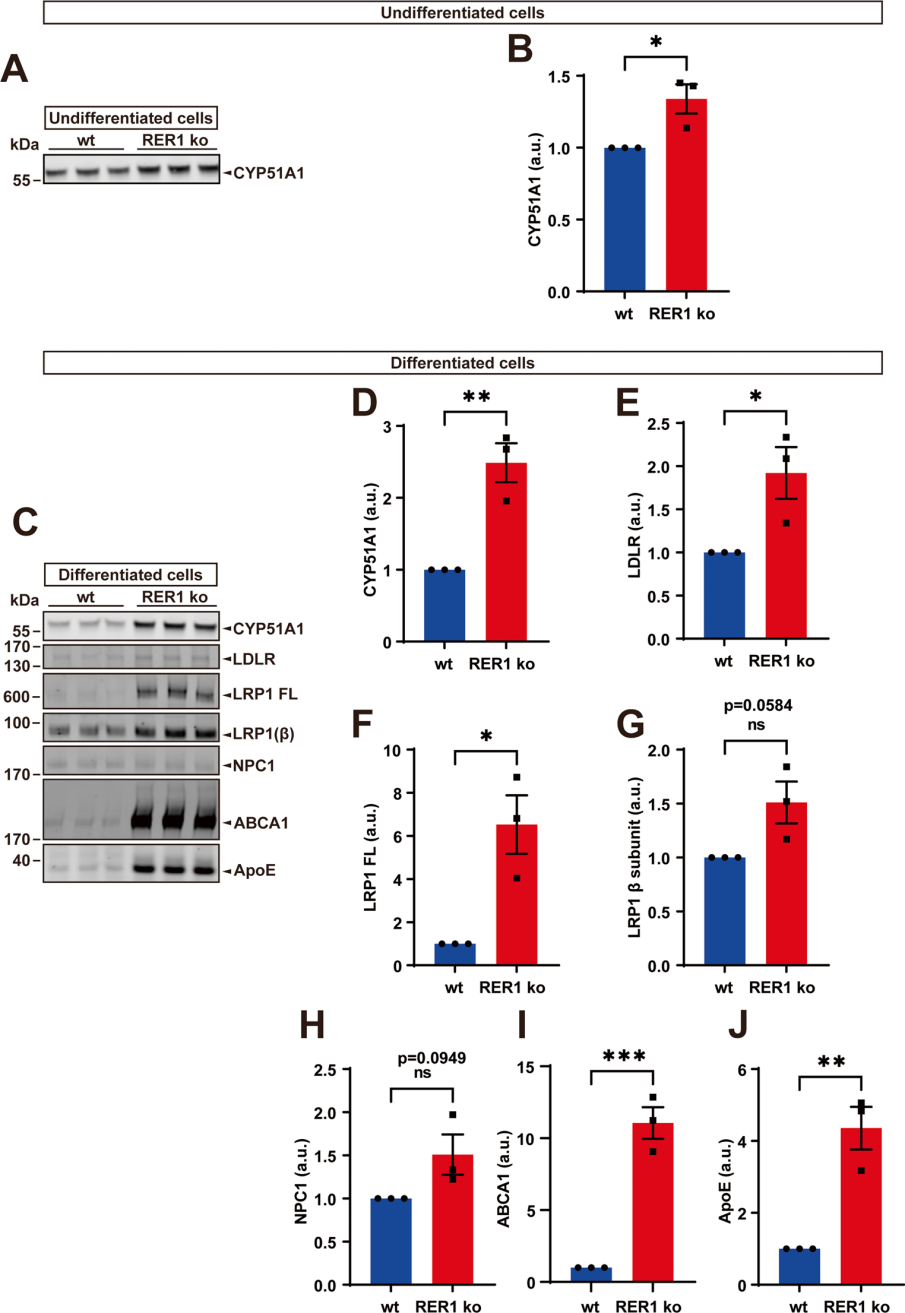
Expressions of lipid metabolism related proteins in wt and RER1 ko THP-1 cells. (**A**) and (**C**) Detection of lipid metabolism related proteins in RER1 ko and wt THP-1 undifferentiated (**A**) and differentiated (**C**) cells. Cellular membranes were isolated, and western immunoblotting was used for the detection of the indicated protein. Quantification of, CYP51A1 (**B** and **D**), LDLR (**E**), LRP1 full length (FL, **F**), LRP1 β subunit (**G**), NPC1 (**H**), ABCA1 (**I**) and ApoE (**J**) by western immunoblotting (as shown in **A** and **C**). Indicated proteins were normalized to the full protein stained by ponceau. Values represent mean ± SEM of three independent experiments with one to three samples per experiment. Each data point represents the mean value of an individual experiment. Student’s t-test (unpaired, two-tailed). *p < 0.05, **p < 0.01, ***p < 0.001

We further analyzed protein expression of lanosterol 14-alpha demethylase (CYP51A1), which is an enzyme known to catalyze the removal of the 14 alpha-methyl group from lanosterol [37]. CYP51A1 protein was increased in RER1-deficient monocytic and macrophage-like differentiated cells as compared to the respective wt cells (Fig. 8A-D), corresponding with their increased mRNA levels (Suppl. Figure 3–5). In addition, proteins related to the uptake, delivery and intracellular transport of cholesterol, including low density lipoprotein receptor (LDLR), low density lipoprotein receptor-related protein 1 (LRP1) and Nieman-Pick C proteins 1 (NPC1), were increased in RER1-deficient, macrophage-like cells as compared to wt cells (Fig. 8C and E-H). Additionally, the level of ATP binding cassette subfamily A member 1 (ABCA1) and Apolipoprotein E (ApoE) which function during cholesterol efflux, were also strongly increased in RER1-deficient macrophage-like cells as compared to wt cells (Fig. 8C, I and J). These data on protein expression levels are consistent with a specific upregulation of these components at the mRNA level in macrophage-like cells (Suppl. Figure 4).

## Discussion

The presented data demonstrate an important role of RER1 in the lipid metabolism of monocytic and macrophage-like cells. The deficiency of RER1 resulted in the accumulation of lipid droplets, complex changes of cholesterol and acylglyceride metabolism, and alterations in lipid and immune related gene expression.

LDs are dynamic organelles found in different cell types and serve for storage of lipids in form of TAGs and CEs [25, 26, 38, 39]. LDs thereby contribute to the regulation of important metabolic and signaling pathways and are related to inflammation and immune cell function in several common diseases, including obesity, diabetes, atherosclerosis, neurodegenerative disorders [20–23, 39–43].

Mass spectrometry revealed significantly increased levels of TAGs in RER1-deficient THP-1 cells as compared to wt cells, both in the monocytic and macrophage-like state. Macrophage-like cells also had increased levels of DAGs. Since levels of CEs were unaltered in monocytic or rather decreased in RER1-deficient macrophage-like cells, the accumulation of LDs is likely caused by elevated incorporation of TAGs and DAGs. Indeed, it has been shown that, in addition to TAGs, DAGs are also stored in LDs and play an important role in LD associated lipid metabolism [44, 45].

Despite unaltered levels of total cholesterol and cholesterol esters, monocytic RER1-deficient cells showed strongly increased levels of cholesterol precursors, suggesting upregulation of cholesterol *de novo* synthesis. This upregulation of cholesterol biosynthesis in RER1-deficient monocytic cells mainly involves the Bloch pathway as indicated by elevated levels of lanosterol and desmosterol, and decreased levels of lathosterol. mRNA sequencing also supports an upregulation of genes involved in sterol synthesis pathways in RER1-deficient monocytes. In contrast, macrophage-like RER1-deficient cells, rather show strongly decreased levels of cholesterol precursors and lower levels of total cholesterol. Nonetheless, KEGG analysis of DEGs also revealed upregulation of lipid metabolic pathways in RER1-deficient macrophage-like cells.

However, the identified lipid-related pathways not only include genes related to cholesterol *de novo* synthesis but also genes regulating the uptake, intracellular transport, metabolism, and the secretion of lipids and lipoproteins that could contribute to the observed alterations in the cellular lipid composition in RER1-deficient monocytic and macrophage-like cells.

Lipids and lipid metabolism play critical roles in physiological and pathophysiological functions of monocytes and macrophages [22, 46, 47]. The treatment of monocytes with very-low and low-density lipoproteins induces accumulation of cytosolic neutral lipids and impairs migration [48]. Lipopolysaccharide (LPS) activated pro-inflammatory macrophages accumulate LDs by increased uptake of FAs and synthesis of TAGs and reduced TAG hydrolysis and show impaired inflammatory responses [20, 49]. Importantly, LDs may serve as a reservoir of lipid precursors to support the production of inflammatory mediators, such as eicosanoid-derived leukotrienes and prostaglandins (PGs), which modulate cell proliferation, activation, and migration [47]. Excessive LD storage in monocytes and macrophages is related to different diseases, including obesity, diabetes and atherosclerosis [39–42]. Strong accumulation of LDs mainly containing excessive esterified cholesterol in macrophages results in formation of foam cells accumulated in the arterial wall during atherosclerosis, which is the hallmark of this disease [50–53]. In obese mice, increased accumulation of lipids in adipose tissue macrophages led to their pro-inflammatory polarization, thereby contributing to chronic inflammation and insulin resistance in adipose tissue [54]. Thus, it will be interesting to further dissect the functional implications of RER1 in lipid metabolisms in monocytes and macrophages.

As a protein sorting receptor, RER1 controls expression and subcellular transport of select client proteins, including components of the γ-secretase complex and the TREM2-DAP12 complex, thereby controlling their assembly and subcellular localization. We analyzed the expression of PEN2, nicastrin, presenilin 1 (PS1) and presenilin 2 (PS2) in wt and RER1-deficient undifferentiated and differentiated cells. Indeed, the expression of nicastrin, PEN2, PS1 and PS2 were decreased in both undifferentiated and differentiated RER1-deficient cells as compared to wt cells (Suppl. Figure 9). These findings are consistent with previous studies, showing decreased levels of γ-secretase complex components in RER1-deficient HAP1 cells [9]. Interestingly, LD accumulation was previously described in presenilin (PS) ko mouse embryonic fibroblasts due to the impairment of γ-secretase function [55, 56]. In addition, γ-secretase has also been implicated in the uptake of lipoprotein particles and the cellular cholesterol metabolism [57–60]. Thus, it is possible that altered expression of γ-secretase complexes potentially contributes to the lipid-related phenotypes in the RER1-deficient THP-1 cells. Additionally, our recent results indicate that RER1 deficiency strongly decreases the expression of TREM2-DAP12 complexes, resulting in impaired immune signaling by this complex [15]. TREM2 has been shown to regulate blood cholesterol metabolism in obese mice by influencing the transcriptional profile of macrophages within adipose tissue, establishing a direct connection between TREM2 and lipid metabolism [61]. Cell-specific lipidomics analysis demonstrated that TREM2 deficiency disrupts the regulation of genes involved in lipid metabolism, resulting in CE accumulation in microglia due to impaired cholesterol transport [62], indicating an important role of TREM2 in lipid metabolism. Notably, macrophage-like differentiated TREM2-deficient THP-1 cells, but not undifferentiated cells, had increased levels of perilipin 2 (Supp. Figure 10). It should be noted that undifferentiated THP-1 cells have very low if any expression of TREM2 and DAP12 [15]. Thus, the accumulation of perilipin 2 observed in macrophage-like differentiated THP-1 RER1-deficient cells might involve decreased TREM2-DAP12 signaling. In addition, macrophage-like RER1-deficient and TREM2-deficient cells showed very similar phenotypes with strongly decreased levels of cholesterol and its precursors lanosterol, lathosterol, and desmosterol (Suppl. Figure 11). Levels of cholesterol esters were also decreased to similar extent in RER1-deficient and TREM2-deficient cells. Thus, these data further support that the effects observed in RER1-deficient cells regarding cellular cholesterol metabolism could involve decreased expression of TREM2-DAP12 complexes in the macrophage-like differentiated cell state. Notebly, increased TAGs were also observed in TREM2 ko cells, which was similar to RER1 ko cells (Supp. Figure 12). Additionally, several lipid metabolism related genes that were significantly upregulated in RER1-deficient differentiated cells, were also found to be increased in TREM2-deficient differentiated cells as compared to wt cells, including SQLE, HMGCR, CYP51A1, LDLR, LRP1, NPC1, ABCA1, INSIG1, SC5D (Suppl. Figure 13). However, we also observed differences between TREM2-deficient and RER1-deficient cells. While DAGs were increased in RER1-deficient macrophage-like cells, their levels in TREM2-deficient cells were very similar to that of wt cells (Suppl. Figure 12). These combined results suggest that the RER1 dependent changes in the lipid metabolism of macrophage-like cells, at least in part, could involve decreased expression of TREM2-DAP12 complexes. However, it has also been described that the deficiency of RER1 could cause ER stress [63], which might also contribute to increased formation of LDs [30]. Thus, further investigations are needed to assess a potential functional contribution of γ-secretase and/or TREM2-DAP12 complexes or other molecular mechanisms in the lipid metabolism upon deficiency of RER1 in monocytes and macrophages. It would also be interesting to further investigate the potential roles of RER1 in the regulation of the metabolism and trafficking of additional lipid related proteins, and the functional implications for monocytes and macrophages in vivo. 

The findings collectively demonstrate that the deficiency of RER1 results in the accumulation of lipid droplets and induces significant alterations in cholesterol and glycerolipid metabolism. Furthermore, RER1 plays a critical role in regulating the expression of genes involved in lipid metabolism within monocytes and macrophage-like cells. These insights highlight the importance of RER1 in maintaining lipid homeostasis and its potential impact on immune cell function.

## Electronic supplementary material

Below is the link to the electronic supplementary material.

Supplementary figure 1 **Comparison of perilipin 2 in RER1 ko and wt THP-1 differentiated cells by immunocytochemistry. (A)** Representative images are shown. Cells were co-stained with the perilipin 2 (red) and DAPI (blue). Scale bar = 10μm. **(B)** Quantification of perilipin 2 intensity shown in **(A)**. Three independent experiments were performed, and at least 100 cells per experiment for one cell type were included in the quantification. Values represent mean ± SEM of three independent experiments were used and each data point represents the mean value of an individual experiment. Student’s t-test (unpaired, two-tailed). *p < 0.05.

High Resolution Image (TIF 8970 kb)

Supplementary figure 2 **The schematic of the two cholesterol biosynthesis pathways.** Cholesterol biosynthesis takes place by two pathways, namely, the Kandutsch-Russell and Bloch pathways.

High Resolution Image (TIF 8970 kb)

Supplementary figure 3 **Kyoto Encyclopedia of Genes and Genomes (KEGG) enrichment analysis showed steroid biosynthesis is upregulated in RER1 ko THP-1 undifferentiated cells.** Heatmap of 10 genes which are upregulated in steroid biosynthesis pathway.

High Resolution Image (TIF 8970 kb)

Supplementary figure 4 **Kyoto Encyclopedia of Genes and Genomes (KEGG) enrichment analysis showed lipid metabolism related pathways are upregulated in RER1 ko THP-1 differentiated cells.** Heatmap of lipid metabolism related pathways, including lipid and atherosclerosis **(A)**, cholesterol metabolism **(B)** and steroid biosynthesis **(C)**.

High Resolution Image (TIF 8970 kb)

Supplementary figure 5 **Gene Ontology (GO) biological process enrichment analysis showed lipid metabolism related pathways are upregulated in RER1 ko undifferentiated THP-1 cells.** Heatmap of lipid metabolism related pathways, including cholesterol biosynthetic process **(A)**, sterol biosynthetic process **(B)**, regulation of lipid metabolic process **(C)**, steroid biosynthetic process **(D)**, cholesterol biosynthetic process via desmosterol **(E)**, cholesterol biosynthetic process via lathosterol **(F)** and lipid metabolic process **(G)**.

High Resolution Image (TIF 8970 kb)

Supplementary figure 6 **Gene Ontology (GO) molecular function enrichment analysis stearoyl-CoA 9-desaturase activity is upregulated in RER1 ko undifferentiated THP-1 cells.** Heatmap of two genes which are upregulated in stearoyl-CoA 9-desaturase activity.

High Resolution Image (TIF 8970 kb)

Supplementary figure 7 **Gene Ontology (GO) enrichment analysis of differentially expressed genes (DEGs) in RER1-deficient versus wt undifferentiated THP-1 cells.** Upregulated GO terms for cellular component.

High Resolution Image (TIF 8970 kb)

Supplementary figure 8 **Gene Ontology (GO) enrichment analysis of upregulated DEGs in RER1-deficient and wt differentiated THP-1 cells.** GO enrichment analysis of genes related to biological process **(A)**, cellular component **(B)** and molecular function **(C)** were performed in RER1 ko and wt THP-1 differentiated cells.

High Resolution Image (TIF 8970 kb)

Supplementary figure 9 **Analysis of γ-secretase components and amyloid precursor protein (APP) in wt and RER1 ko THP-1 cells. (A)** and **(C)** Detection of γ-secretase complex components, and APP in RER1 ko and wt THP-1 undifferentiated **(A)** and differentiated **(C)** cells. Cellular membranes were isolated, and western immunoblotting was used for the detection of the indicated protein. **(B**) and **(D)** Quantification of Nicastrin (mature and immature forms), PEN2, PS1 (C-terminal fragments, CTF and N-terminal fragments, NTF), PS2 (CTF) and APP (full length, FL and CTF) by western immunoblotting (as shown in **A** and **C**). Indicated proteins were normalized to the full protein stained by ponceau. Values represent mean ± SEM of three to five independent experiments with one to three samples per experiment. Each data point represents the mean value of an individual experiment. Student’s t-test (unpaired, two-tailed). *p < 0.05, **p < 0.01, ***p < 0.001.

High Resolution Image (TIF 8970 kb)

Supplementary figure 10 **Comparison of perilipin 2 expression level in TREM2 ko and wt THP-1 differentiated cells. (A)** Detection of perilipin 2 in TREM2 ko and wt THP-1 differentiated cells. Cellular membranes were isolated and western immunoblotting was used for the detection of perilipin 2. **(B)** Quantification of perilipin 2 by western immunoblotting (as shown in **A**). Perilipin 2 was normalized to the full protein stained by Ponceau. Data represent mean ± SEM of five independent experiments with one to three samples per experiment. **(C)** Comparison of perilipin 2 in TREM2 ko and wt THP-1 differentiated cells by immunocytochemistry. Representative images are shown. Cells were co-stained with the perilipin 2 (red) and DAPI (blue). Scale bar = 10μm. **(D)** Quantification of perilipin 2 intensity shown. Each data point represents the mean value of an individual experiment. Student’s t-test (unpaired, two-tailed). *p < 0.05.

High Resolution Image (TIF 8970 kb)

Supplementary figure 11 **Content analysis of a panel of sterols in TREM2 ko and wt THP-1 differentiated cells, determined by GC-FID and GC-MS-SIM.** Free cholesterol and cholesteryl esters are expressed as a percentage of total cholesterol. Total cholesterol (**A**, absolute amount), free cholesterol and cholesterol esters (**B**, % of total cholesterol) as well as cholesterol precursors (lanosterol **(C)**, desmosterol **(D)**, lathosterol **(E)** were analyzed in THP-1 differentiated cells. Values represent mean ± SEM of three independent experiments each performed with triplicate samples. Each data point represents the mean value of an individual experiment. Student’s t-test (unpaired, two-tailed). *p < 0.05, **p < 0.01, ***p < 0.001, ****p < 0.0001.

High Resolution Image (TIF 8970 kb)

Supplementary figure 12 **Analysis of cholesterol esters (CEs) species, diacylglycerol (DAGs) species and triacylglycerol (TAGs) species by tandem mass spectrometry in TREM2 ko and wt differentiated cells.** Total CEs **(A)** and different species **(B)**, total diacylglycerol **(C)** and different species **(D)**, total triacylglycerol **(E)** and different species **(F)** were analyzed in TREM2 ko and wt THP-1 differentiated cells. Values represent mean ± SEM of three independent experiments each performed with triplicate samples. Each data point represents the mean value of an individual replicate. Student’s t-test (unpaired, two-tailed). **p < 0.01. ***p < 0.001, ****p < 0.0001.

High Resolution Image (TIF 8970 kb)

Supplementary figure 13 **The upregulated lipid related genes and proteins in TREM2 ko differentiated cells as compared to wt cells. (A)** The overlapping upregulated genes that are related to lipid metabolism between RER1 ko and TREM2 ko THP-1 cells as compared to THP-1 wt cells. **(B)** Expressions of lipid metabolism related proteins in wt and TREM2 ko THP-1 differentiated cells. Cellular membranes were isolated, and western immunoblotting was used for the detection of the indicated protein. Quantification of CYP51A1, LDLR, LRP1, NPC1, ABCA1 and ApoE by western immunoblotting. Indicated proteins were normalized to the full protein stained by ponceau. Values represent mean ± SEM of three independent experiments with one to three samples per experiment. Each data point represents the mean value of an individual experiment. Student’s t-test (unpaired, two-tailed). **p < 0.01, ****p < 0.0001.

High Resolution Image (TIF 8970 kb)

Supplementary figure 14 **Accumulation of lipid droplets (LDs) in different RER1 ko clones. (A)** Detection of perilipin 2 in three different RER1 ko clones and wt THP-1 undifferentiated and differentiated cells. Cellular membranes were isolated and western immunoblotting was used for the detection of the indicated protein (clone 1: R1A7, clone 2: R2A10, clone 3: R1C7 (which was used for all other experiments)). **(B)** Comparison of LDs in different RER1 ko clones and wt THP-1 differentiated cells by LD540 staining. Representative images are shown. Cells were co-stained with LD540 (red) and DAPI (blue) to visualize LDs and nuclei, respectively. Scale bar = 10μm. LDs numbers and volume per cell were quantified by automated LD quantification (ALDQ) method. **(C)** Comparison of perilipin 2 in RER1 ko and wt THP-1 differentiated cells by immunocytochemistry. Representative images are shown. Cells were co-stained with the perilipin 2 (red) and DAPI (blue). Scale bar = 10μm.

High Resolution Image (TIF 8970 kb)

Supplementary Material 15

## Data Availability

Original data and materials are available upon reasonable request.

## Abbreviations

ABCA1: Transporter proteins ATP-binding cassette transporter 1
AChR: Acetylcholine Receptor
ALDQ: Automatic Lipid Droplet Quantification
ApoE: Apolipoprotein E
BSA: Bovine Serum Albumin
CEs: Cholesterol esters
CMT disease: Charcot-Marie-Tooth disease
CYP51A1: Cytochrome P450 Family 51 Subfamily A Member 1
DAP12: DNAX-activating protein of 12 kDa
DAPI: 4′,6-Diamidino-2-Phenylindole
DEGs: Differentially expressed genes
ER: Endoplasmic reticulum
ERGIC: ER-Golgi Intermediate Compartment
FAs: Fatty acids
FBS: Fetal Bovine Serum
GC-FID: chromatography-flame ionization detection
GC–MS-SIM: GC-mass spectrometry selected ion monitoring
GO: Gene Ontology
KEGG: Kyoto Encyclopedia of Genes and Genomes
ko: Knock out
LDLR: Low density lipoprotein receptor
LDs: Lipid droplets
LPS: Lipopolysaccharide
LRP1: Low density lipoprotein receptor-related protein 1
NPC1: Nieman-Pick C proteins 1
P/S: Penicillin/Streptomycin
PBS: Phosphate Buffer Saline
PBST: Phosphate-Buffered Saline, 0.1% Tween® 20 Detergent
PEN2: Presenilin Enhancer 2
PFA: Paraformaldehyde
PMA: Phorbol 12-Myristate 13-Acetate
PMP22: Peripheral Myelin Protein 22
PS: Presenilin
PS1: Presenilin 1
PS2: Presenilin 2
RER1: Retention in ER Sorting Receptor 1
RNA seq: RNA sequencing
RT: Room Temperature
SDS-PAGE: Sodium dodecyl-sulfate polyacrylamide gel electrophoresis
TAGs: Triacylglycerols
TBST: Tris-Buffered Saline, 0.1% Tween® 20 Detergent
TPM: Transcript per million
TREM2: Triggering Receptor Expressed on Myeloid cells 2
wt: Wild type
