# Supplementary figures and images for: RER1 regulates lipid metabolism in monocytes and macrophages
Source: Cell Mol Life Sci. 2025 Aug 13;82(1):313. doi: 10.1007/s00018-025-05817-3 (PMC12351005; doi:10.1007/s00018-025-05817-3)

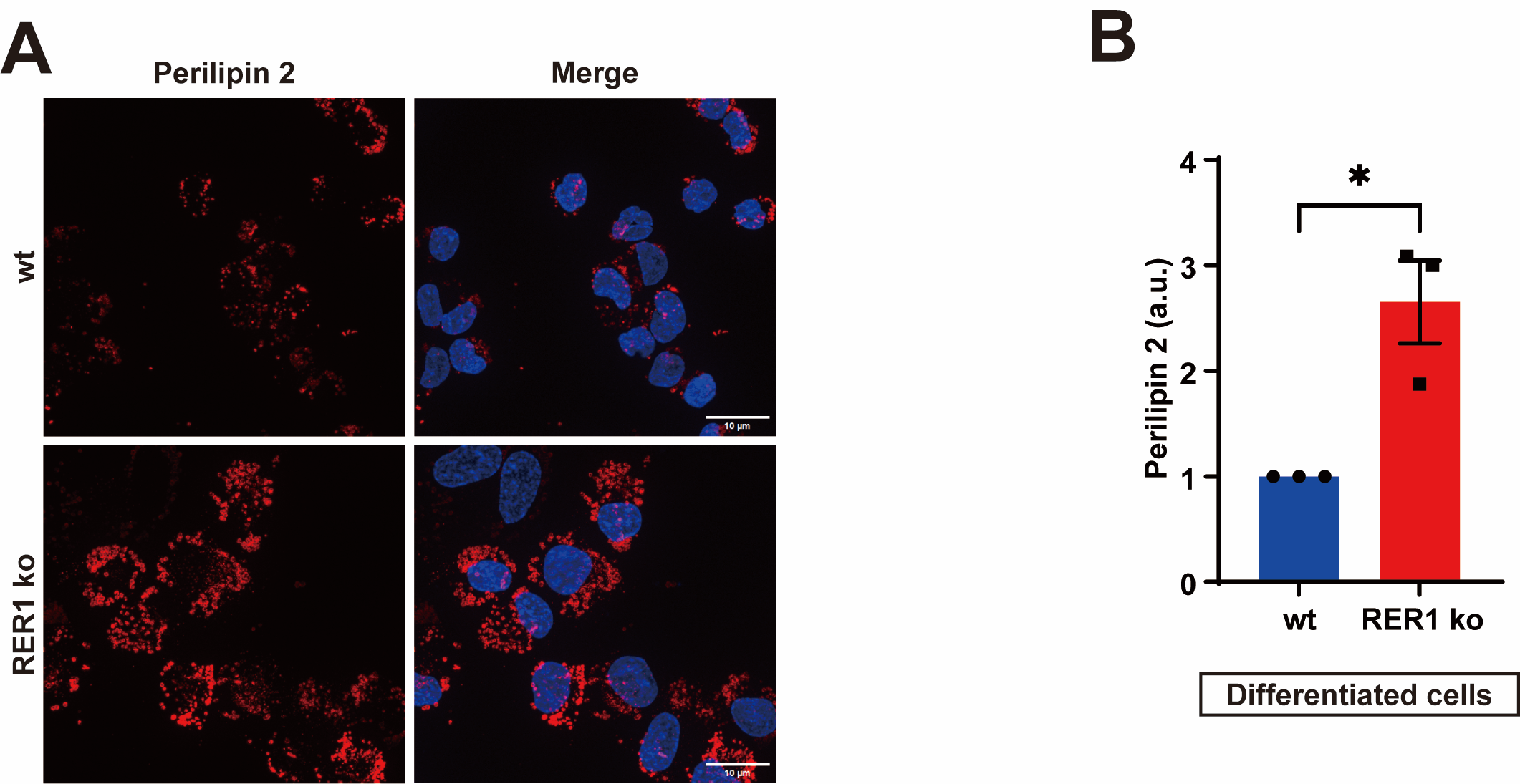

Supplement: Supplementary file 1 — Comparison of perilipin 2 in RER1 ko and wt THP-1 differentiated cells by immunocytochemistry. (A) Representative images are shown. Cells were co-stained with the perilipin 2 (red) and DAPI (blue). Scale bar = 10μm. (B) Quantification of perilipin 2 intensity shown in (A). Three independent experiments were performed, and at least 100 cells per experiment for one cell type were included in the quantification. Values represent mean ± SEM of three independent experiments were used and each data point represents the mean value of an individual experiment. Student’s t-test (unpaired, two-tailed). *p < 0.05. [file 18_2025_5817_Fig9_ESM.png]

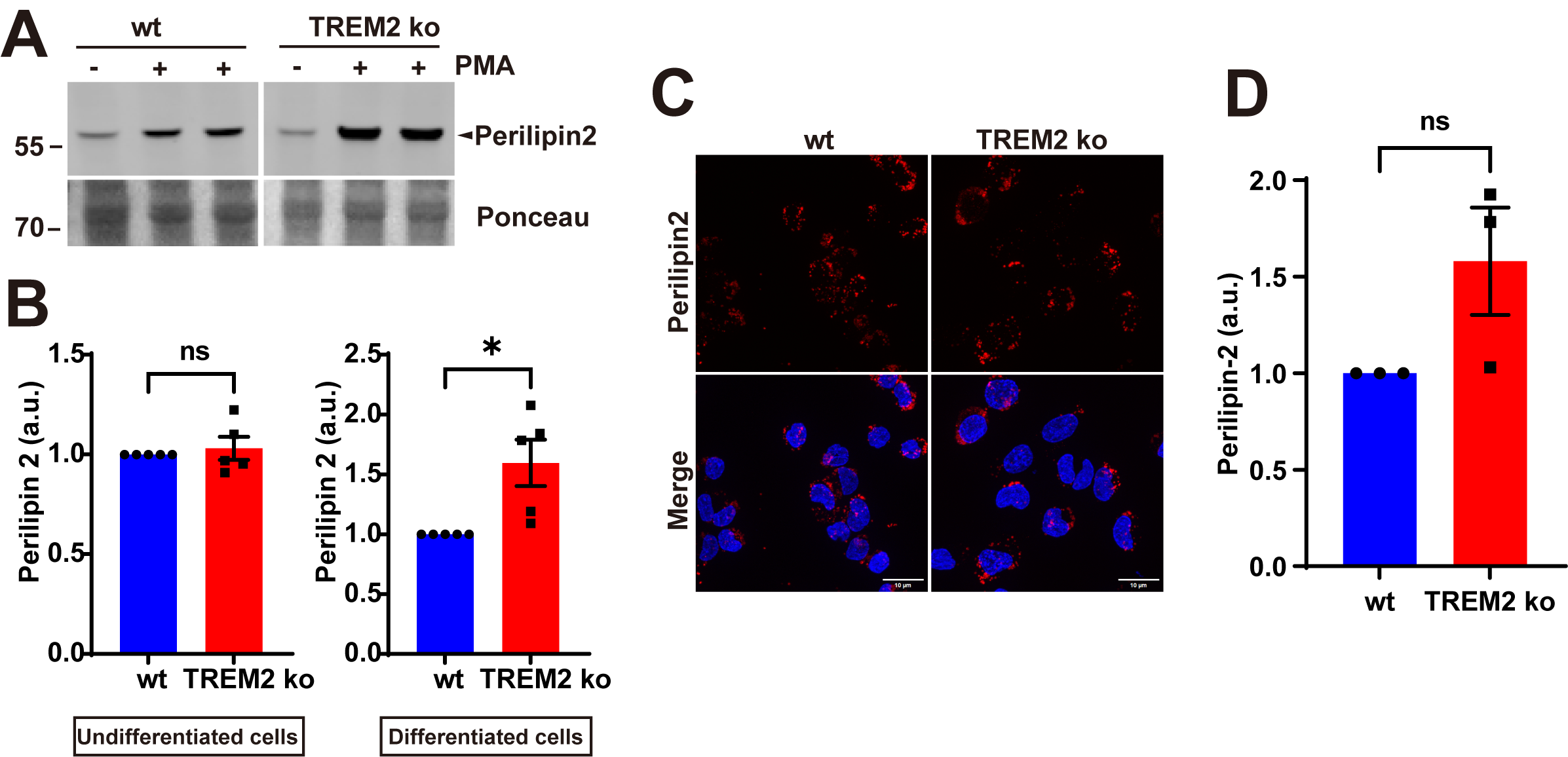

Supplement: Supplementary file 2 — High Resolution Image (TIF 8970 kb) [file 18_2025_5817_MOESM2_ESM.tif]

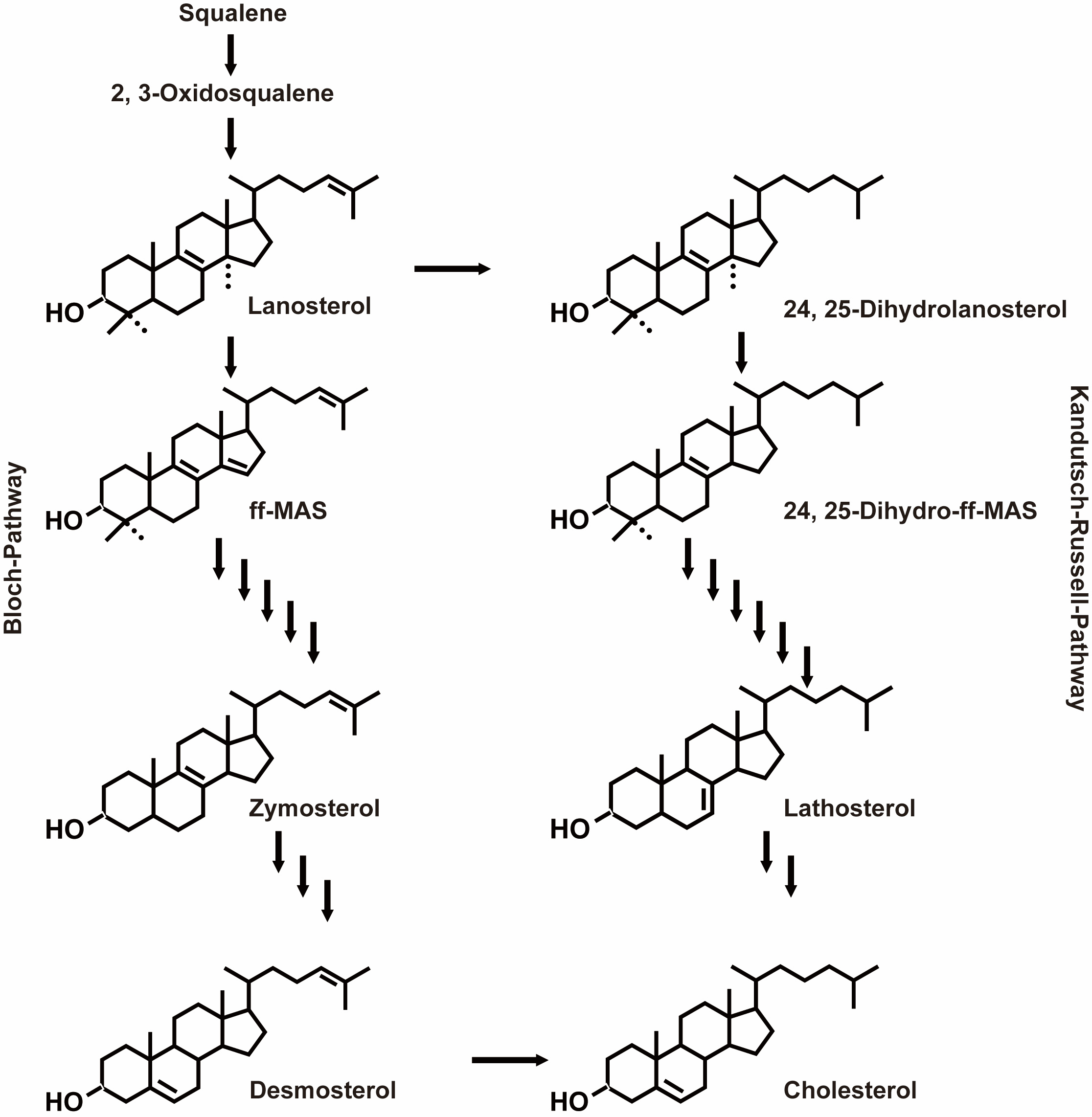

Supplement: Supplementary file 3 — The schematic of the two cholesterol biosynthesis pathways. Cholesterol biosynthesis takes place by two pathways, namely, the Kandutsch-Russell and Bloch pathways. [file 18_2025_5817_Fig10_ESM.png]

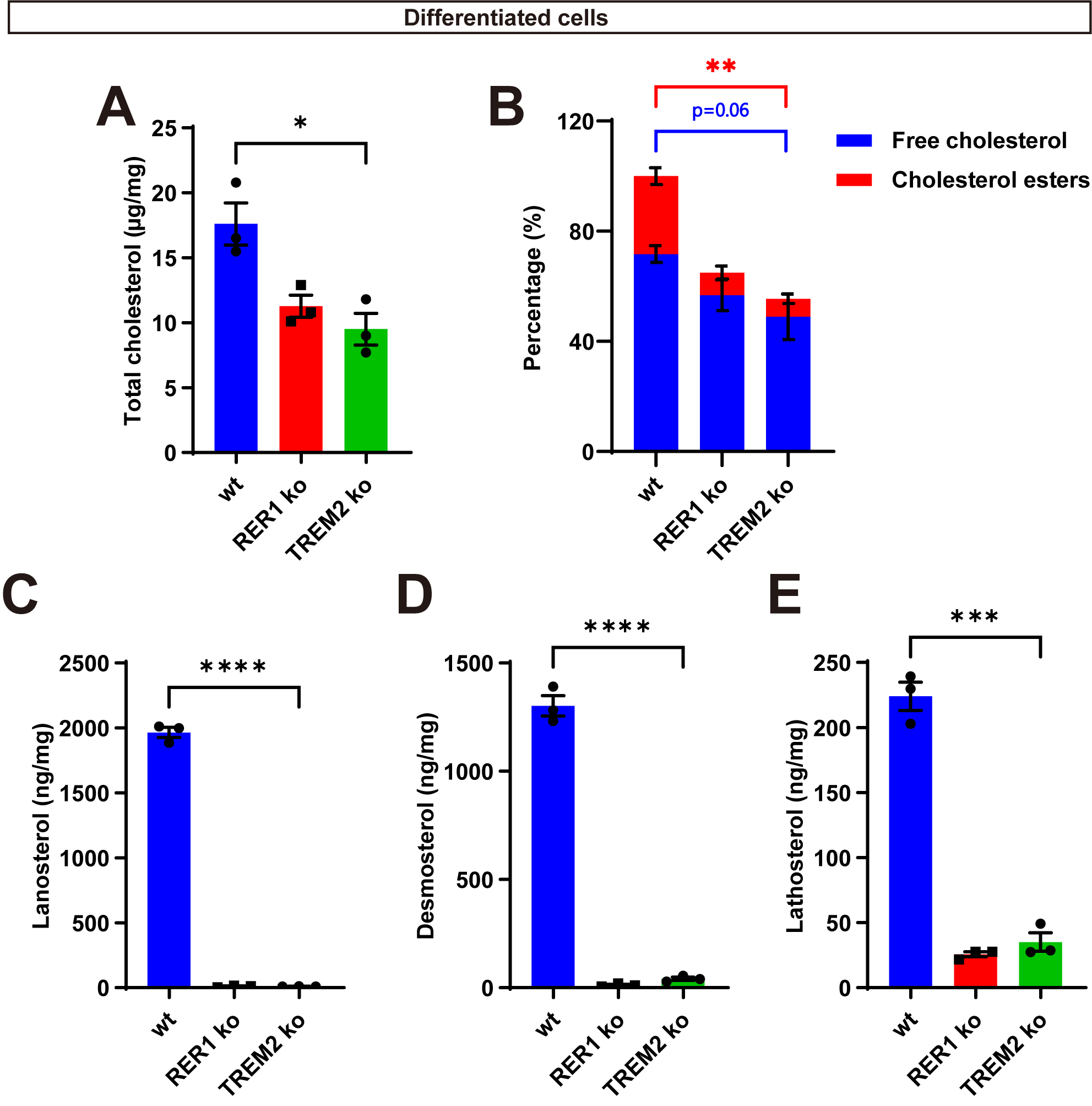

Supplement: Supplementary file 4 — High Resolution Image (TIF 8970 kb) [file 18_2025_5817_MOESM4_ESM.tif]

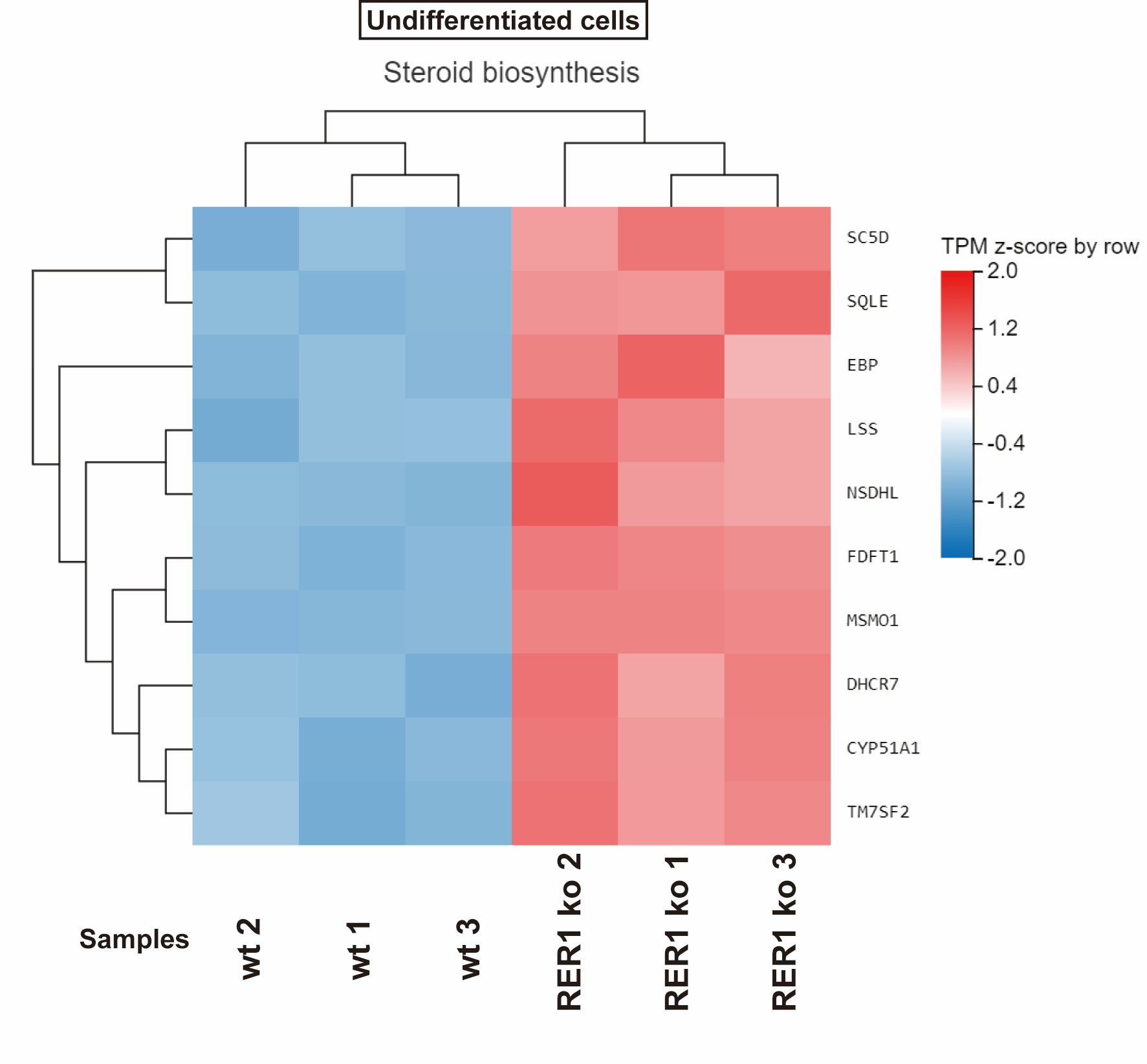

Supplement: Supplementary file 5 — Kyoto Encyclopedia of Genes and Genomes (KEGG) enrichment analysis showed steroid biosynthesis is upregulated in RER1 ko THP-1 undifferentiated cells. Heatmap of 10 genes which are upregulated in steroid biosynthesis pathway. [file 18_2025_5817_Fig11_ESM.png]

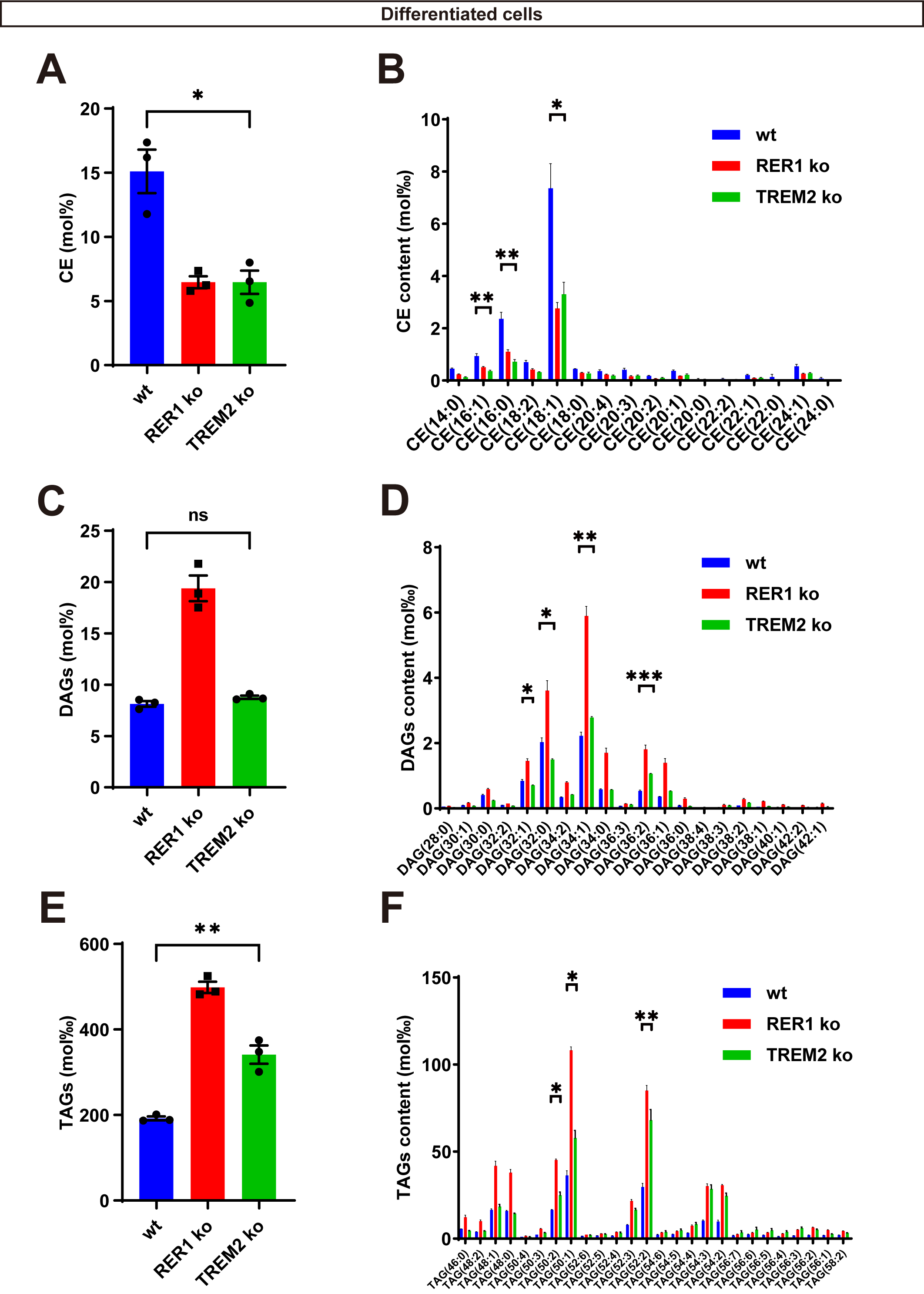

Supplement: Supplementary file 6 — High Resolution Image (TIF 8970 kb) [file 18_2025_5817_MOESM6_ESM.tif]

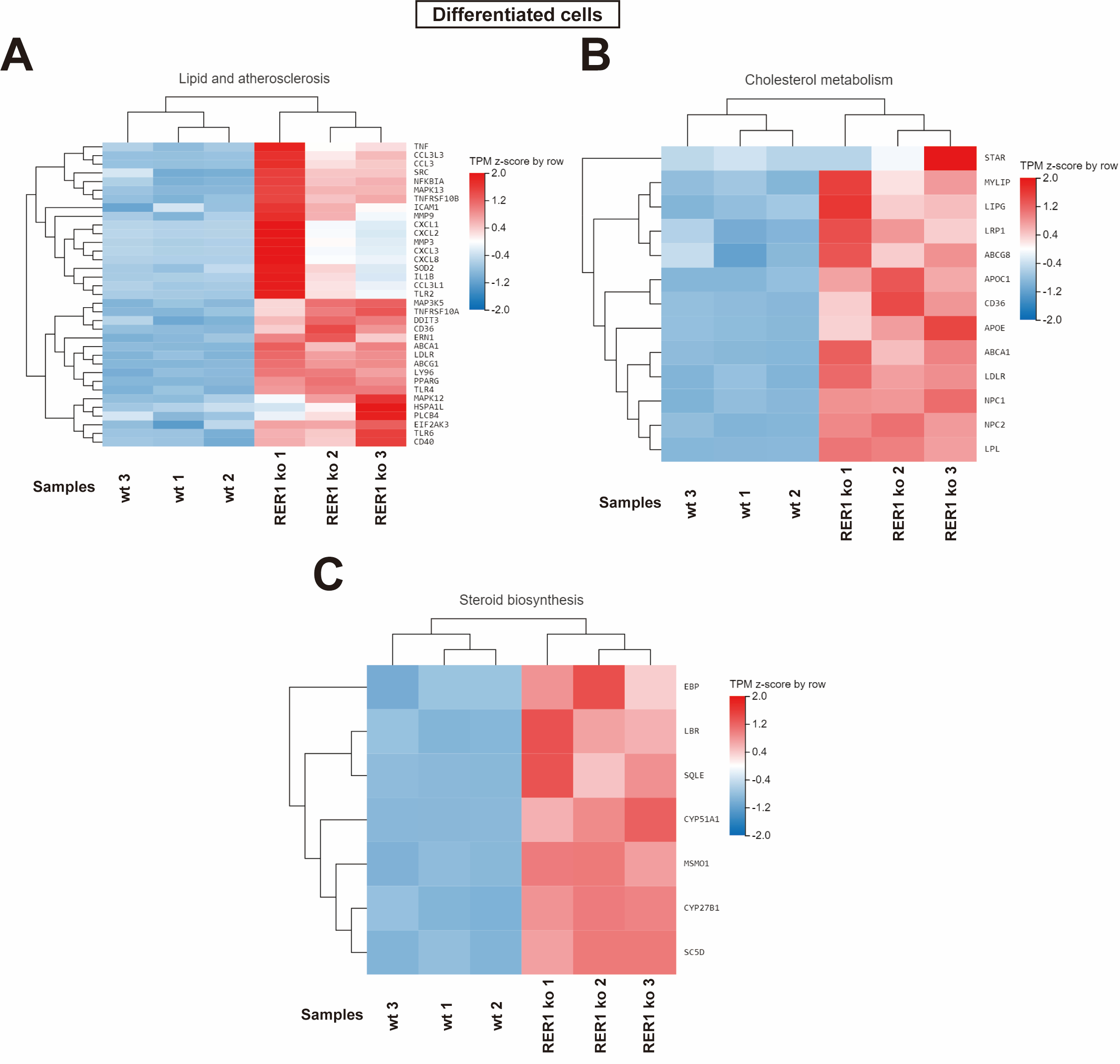

Supplement: Supplementary file 7 — Kyoto Encyclopedia of Genes and Genomes (KEGG) enrichment analysis showed lipid metabolism related pathways are upregulated in RER1 ko THP-1 differentiated cells. Heatmap of lipid metabolism related pathways, including lipid and atherosclerosis (A), cholesterol metabolism (B) and steroid biosynthesis (C). [file 18_2025_5817_Fig12_ESM.png]

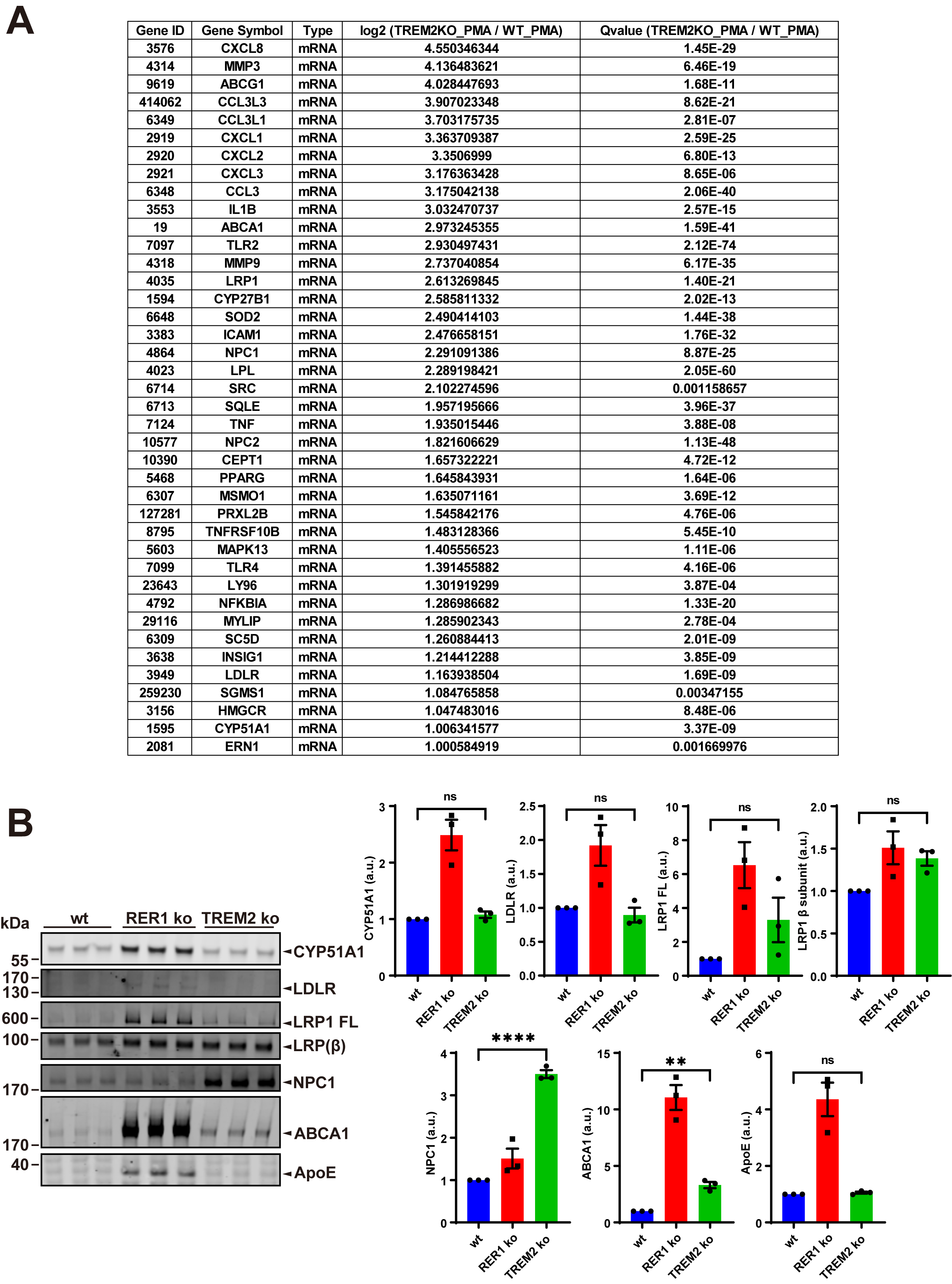

Supplement: Supplementary file 8 — High Resolution Image (TIF 8970 kb) [file 18_2025_5817_MOESM8_ESM.tif]

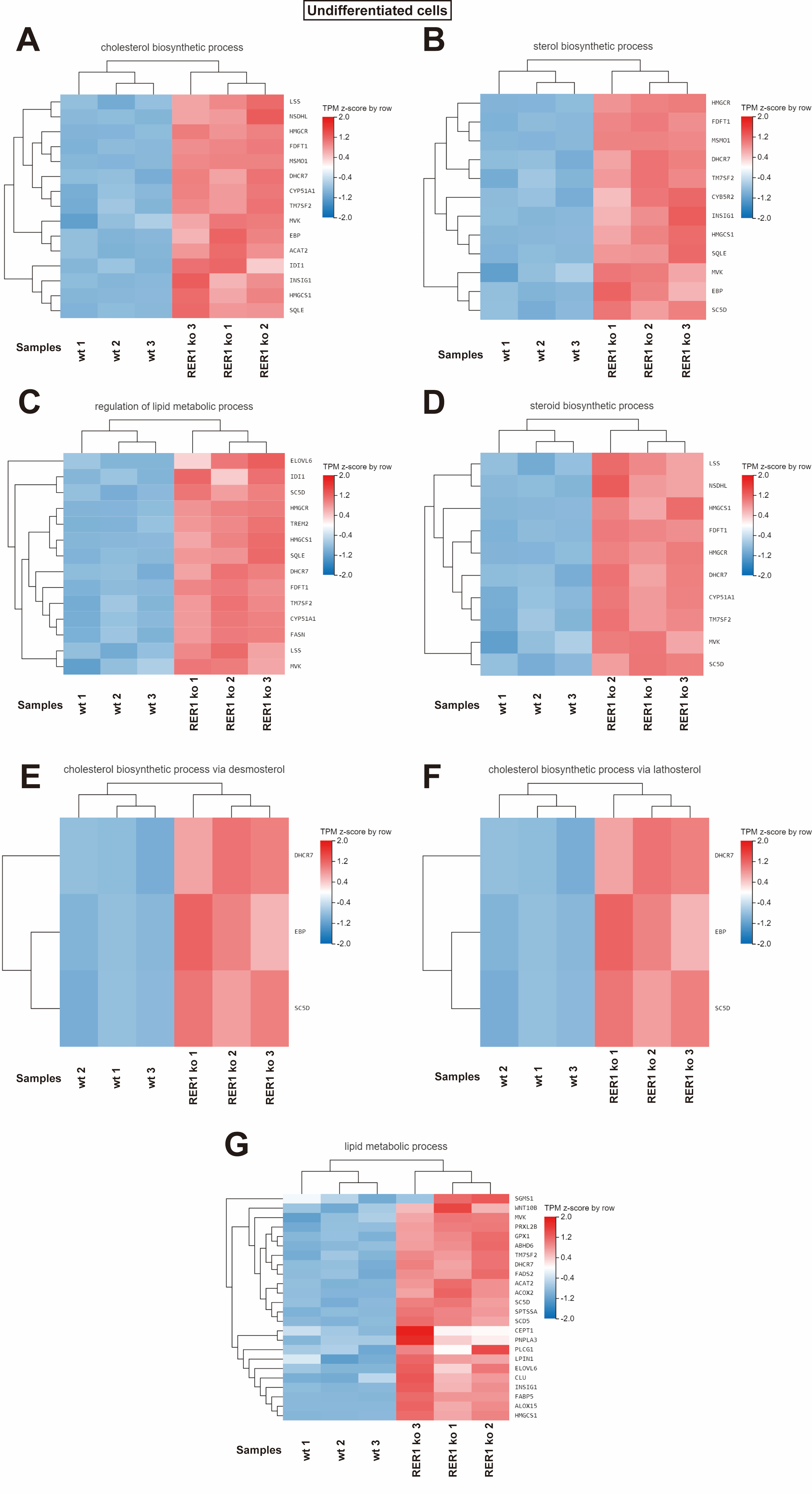

Supplement: Supplementary file 9 — Gene Ontology (GO) biological process enrichment analysis showed lipid metabolism related pathways are upregulated in RER1 ko undifferentiated THP-1 cells. Heatmap of lipid metabolism related pathways, including cholesterol biosynthetic process (A), sterol biosynthetic process (B), regulation of lipid metabolic process (C), steroid biosynthetic process (D), cholesterol biosynthetic process via desmosterol (E), cholesterol biosynthetic process via lathosterol (F) and lipid metabolic process (G). [file 18_2025_5817_Fig13_ESM.png]

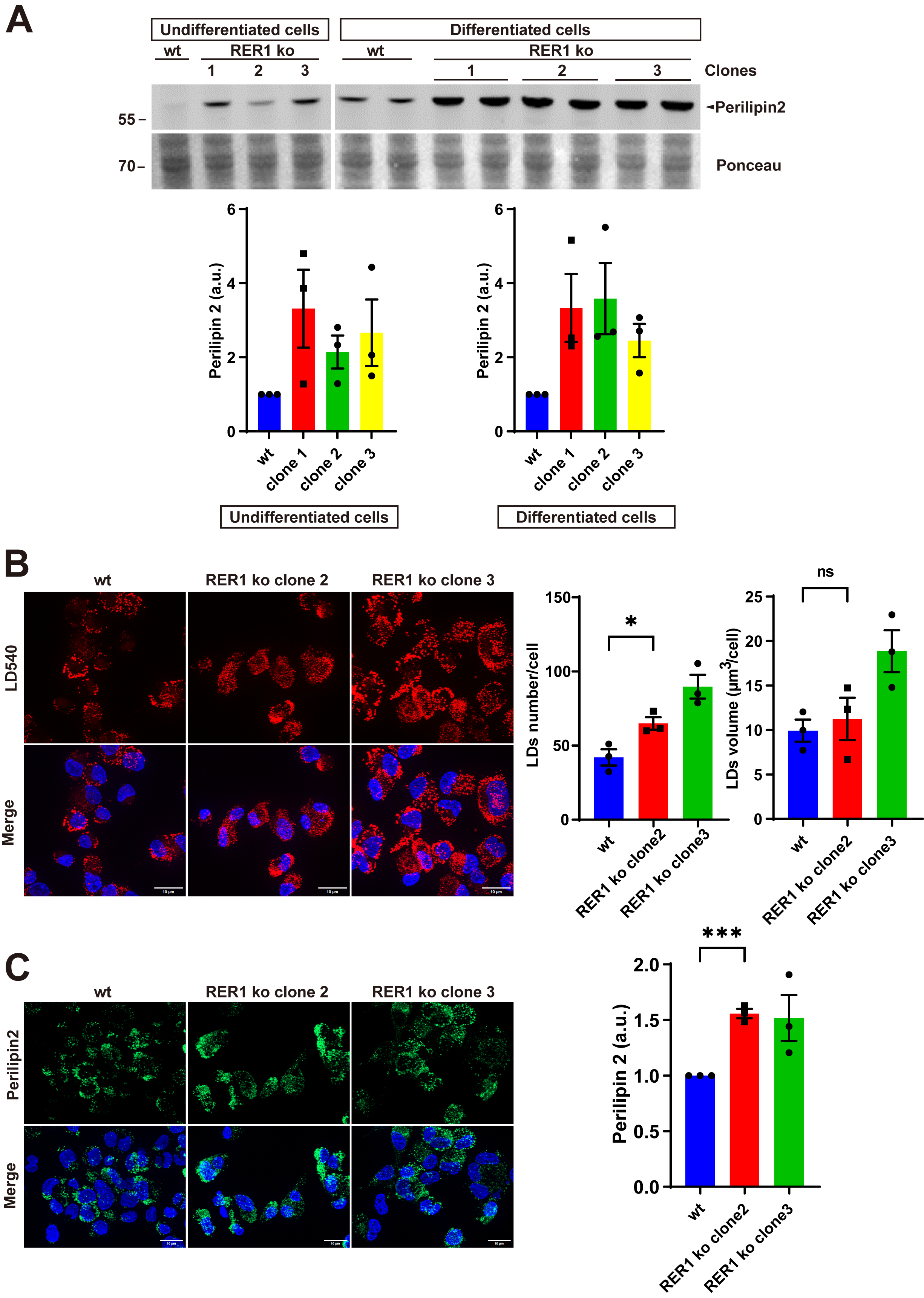

Supplement: Supplementary file 10 — High Resolution Image (TIF 8970 kb) [file 18_2025_5817_MOESM10_ESM.tif]

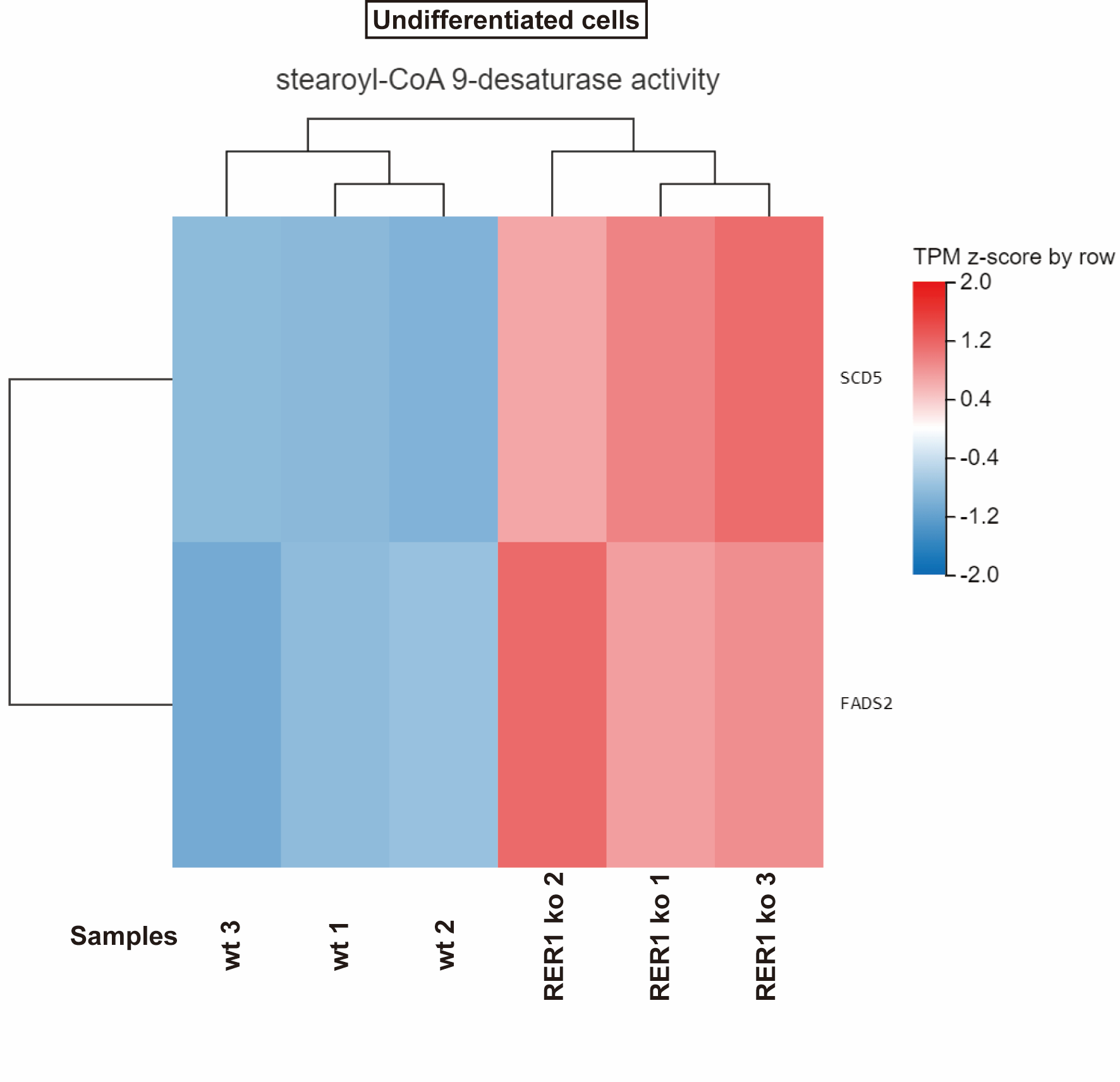

Supplement: Supplementary file 11 — Gene Ontology (GO) molecular function enrichment analysis stearoyl-CoA 9-desaturase activity is upregulated in RER1 ko undifferentiated THP-1 cells. Heatmap of two genes which are upregulated in stearoyl-CoA 9-desaturase activity. [file 18_2025_5817_Fig14_ESM.png]

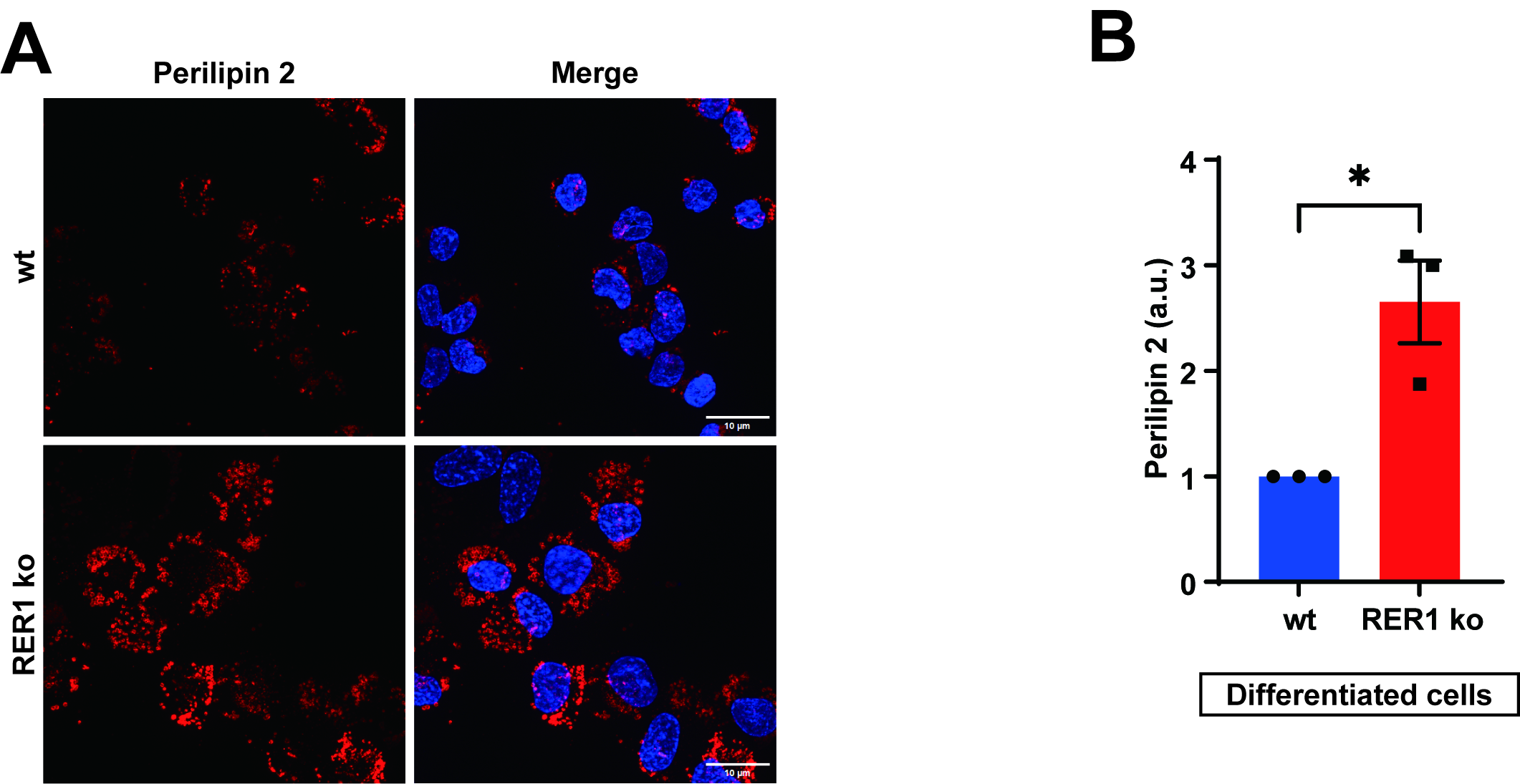

Supplement: Supplementary file 12 — High Resolution Image (TIF 8970 kb) [file 18_2025_5817_MOESM12_ESM.tif]

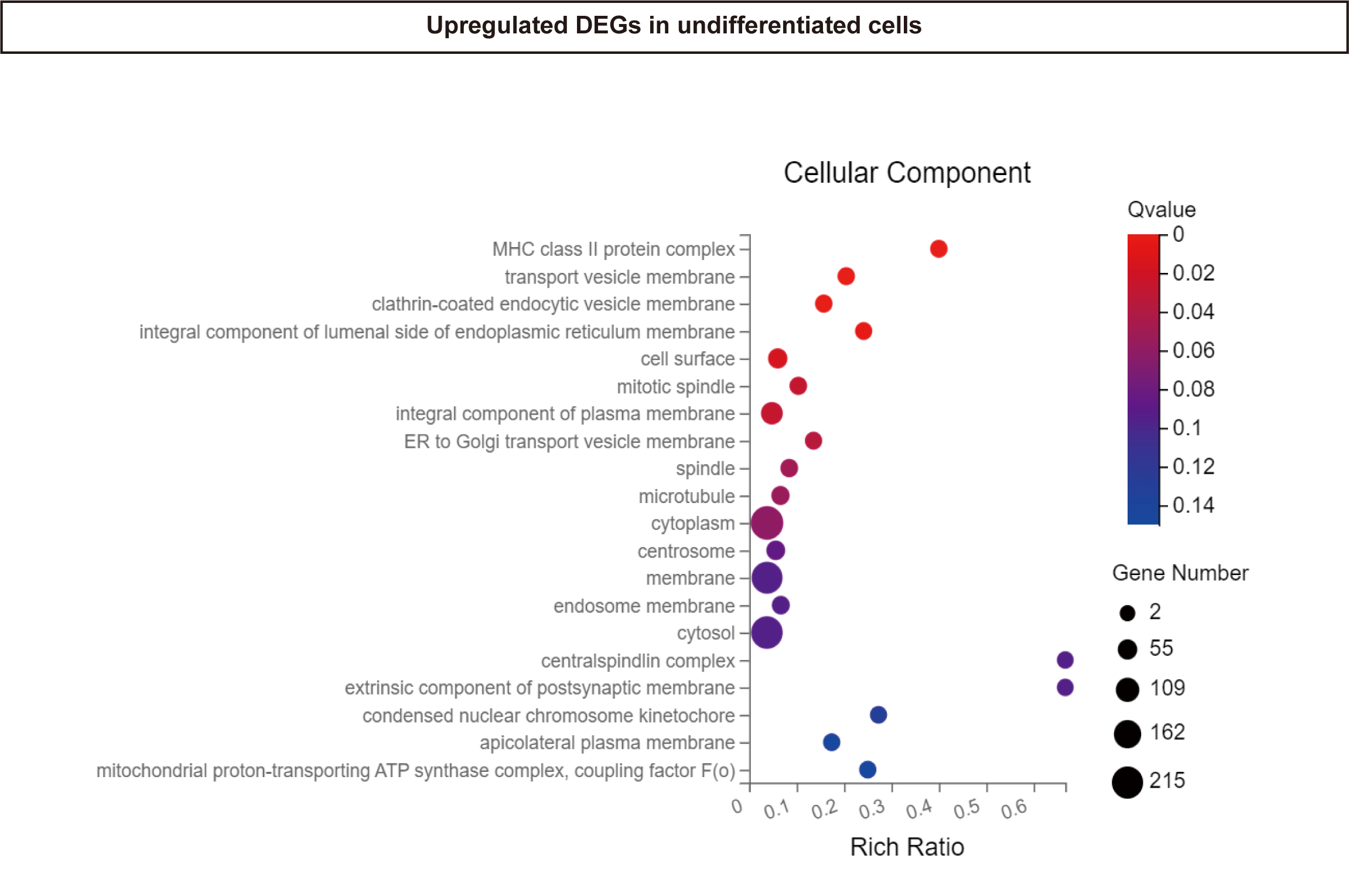

Supplement: Supplementary file 13 — Gene Ontology (GO) enrichment analysis of differentially expressed genes (DEGs) in RER1-deficient versus wt undifferentiated THP-1 cells. Upregulated GO terms for cellular component. [file 18_2025_5817_Fig15_ESM.png]

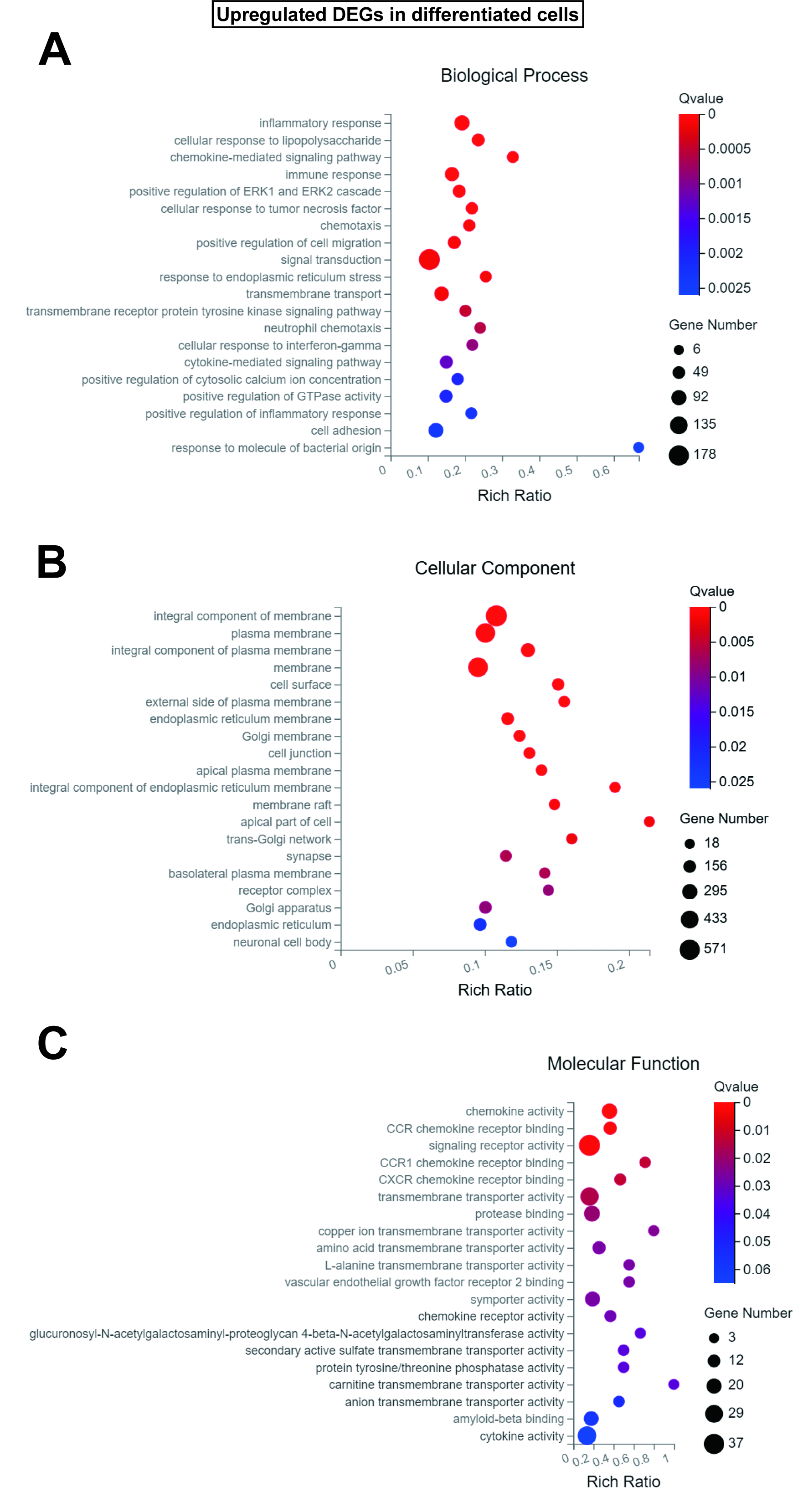

Supplement: Supplementary file 14 — High Resolution Image (TIF 8970 kb) [file 18_2025_5817_MOESM14_ESM.tif]

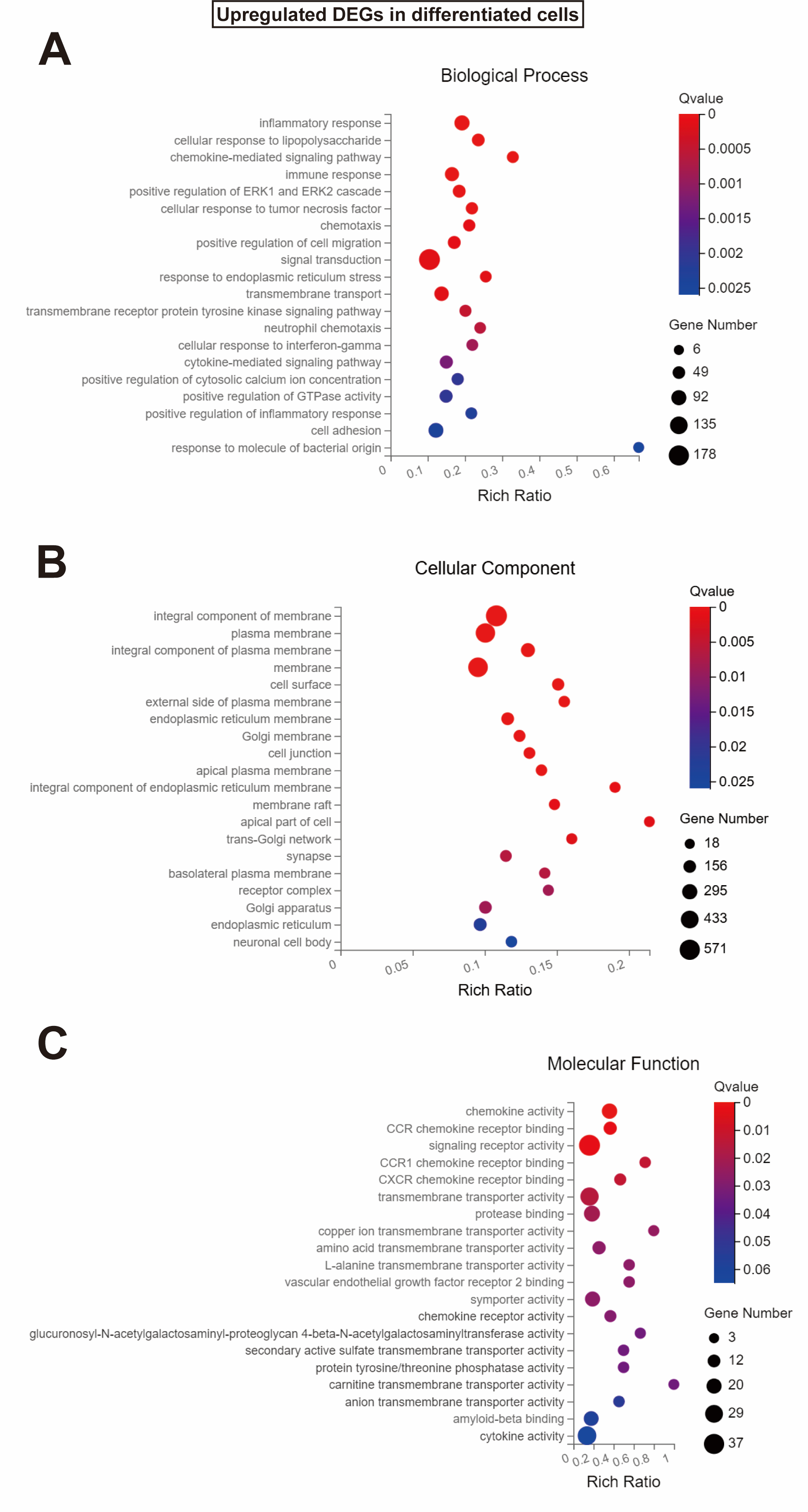

Supplement: Supplementary file 15 — Gene Ontology (GO) enrichment analysis of upregulated DEGs in RER1-deficient and wt differentiated THP-1 cells. GO enrichment analysis of genes related to biological process (A), cellular component (B) and molecular function (C) were performed in RER1 ko and wt THP-1 differentiated cells. [file 18_2025_5817_Fig16_ESM.png]

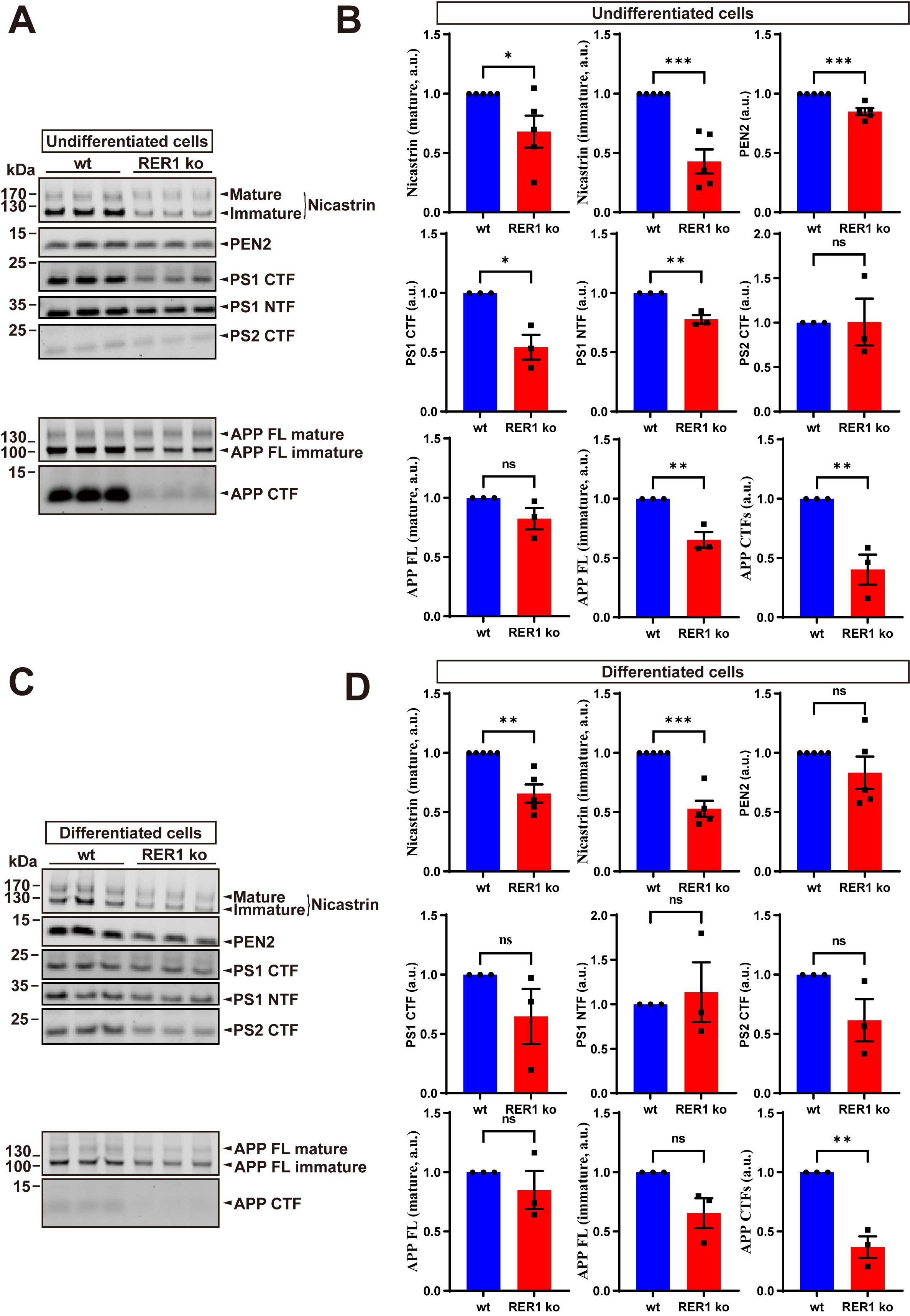

Supplement: Supplementary file 16 — High Resolution Image (TIF 8970 kb) [file 18_2025_5817_MOESM16_ESM.tif]

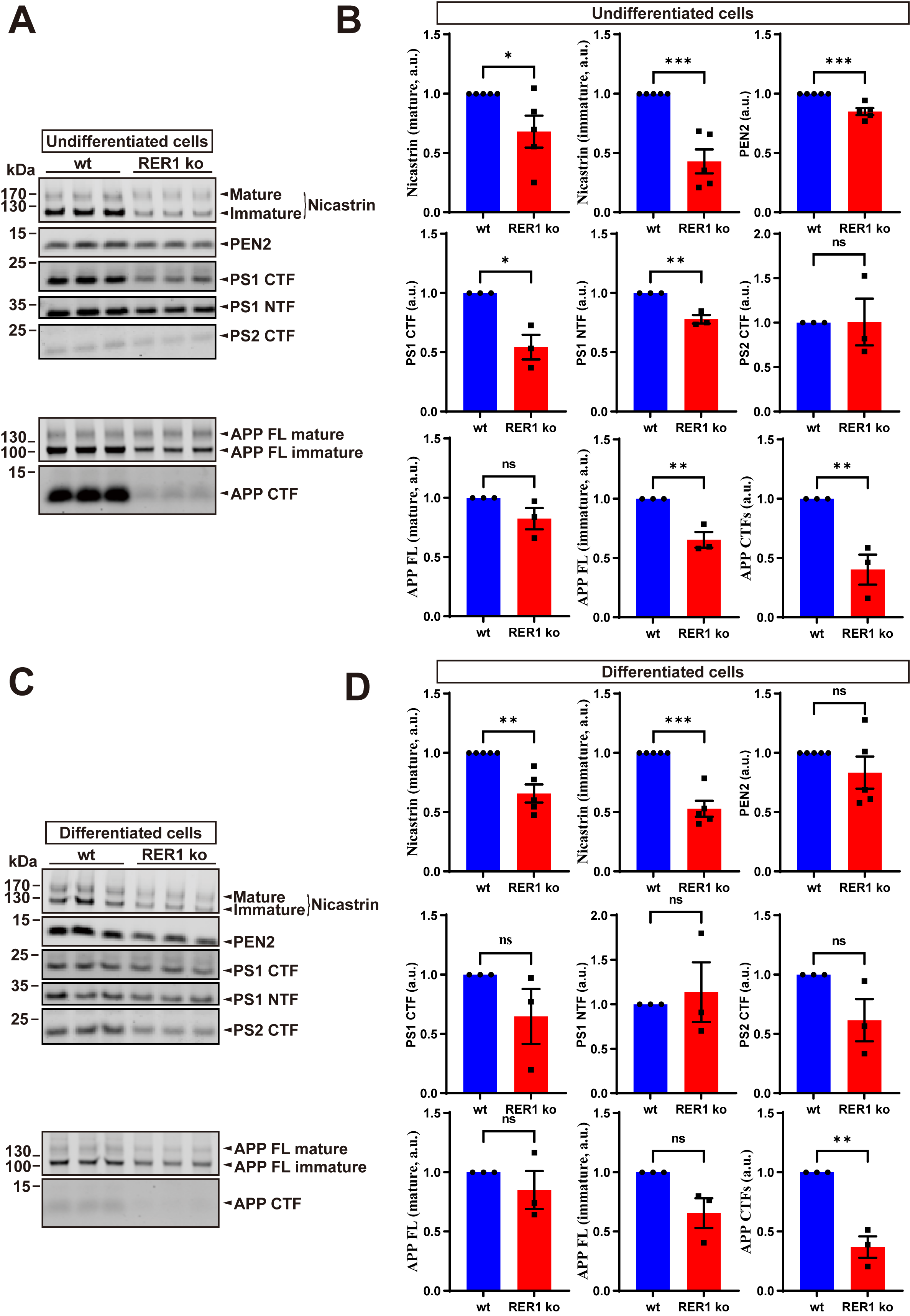

Supplement: Supplementary file 17 — Analysis of γ-secretase components and amyloid precursor protein (APP) in wt and RER1 ko THP-1 cells. (A) and (C) Detection of γ-secretase complex components, and APP in RER1 ko and wt THP-1 undifferentiated (A) and differentiated (C) cells. Cellular membranes were isolated, and western immunoblotting was used for the detection of the indicated protein. (B) and (D) Quantification of Nicastrin (mature and immature forms), PEN2, PS1 (C-terminal fragments, CTF and N-terminal fragments, NTF), PS2 (CTF) and APP (full length, FL and CTF) by western immunoblotting (as shown in A and C). Indicated proteins were normalized to the full protein stained by ponceau. Values represent mean ± SEM of three to five independent experiments with one to three samples per experiment. Each data point represents the mean value of an individual experiment. Student’s t-test (unpaired, two-tailed). *p < 0.05, **p < 0.01, ***p < 0.001. [file 18_2025_5817_Fig17_ESM.png]

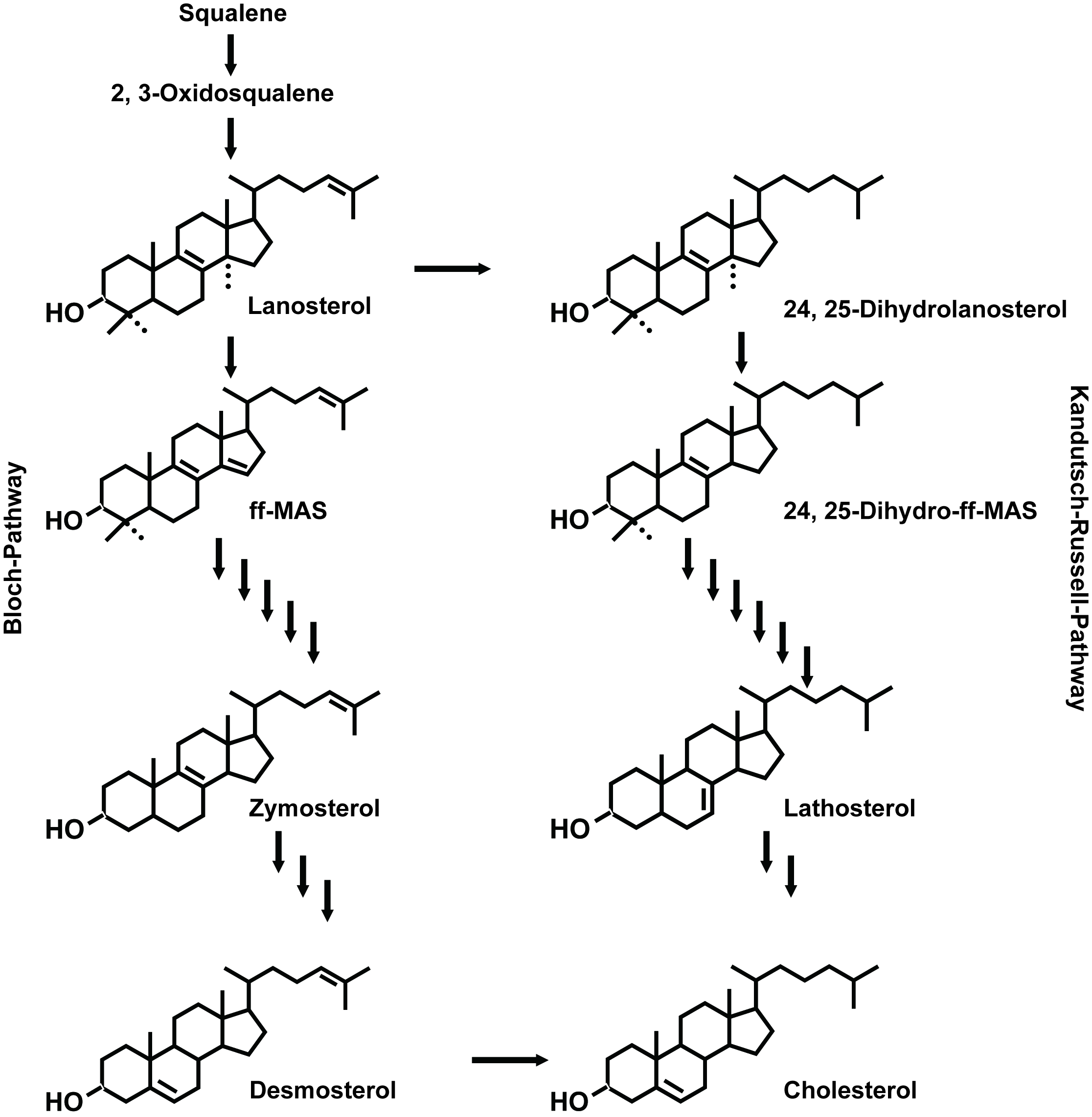

Supplement: Supplementary file 18 — High Resolution Image (TIF 8970 kb) [file 18_2025_5817_MOESM18_ESM.tif]

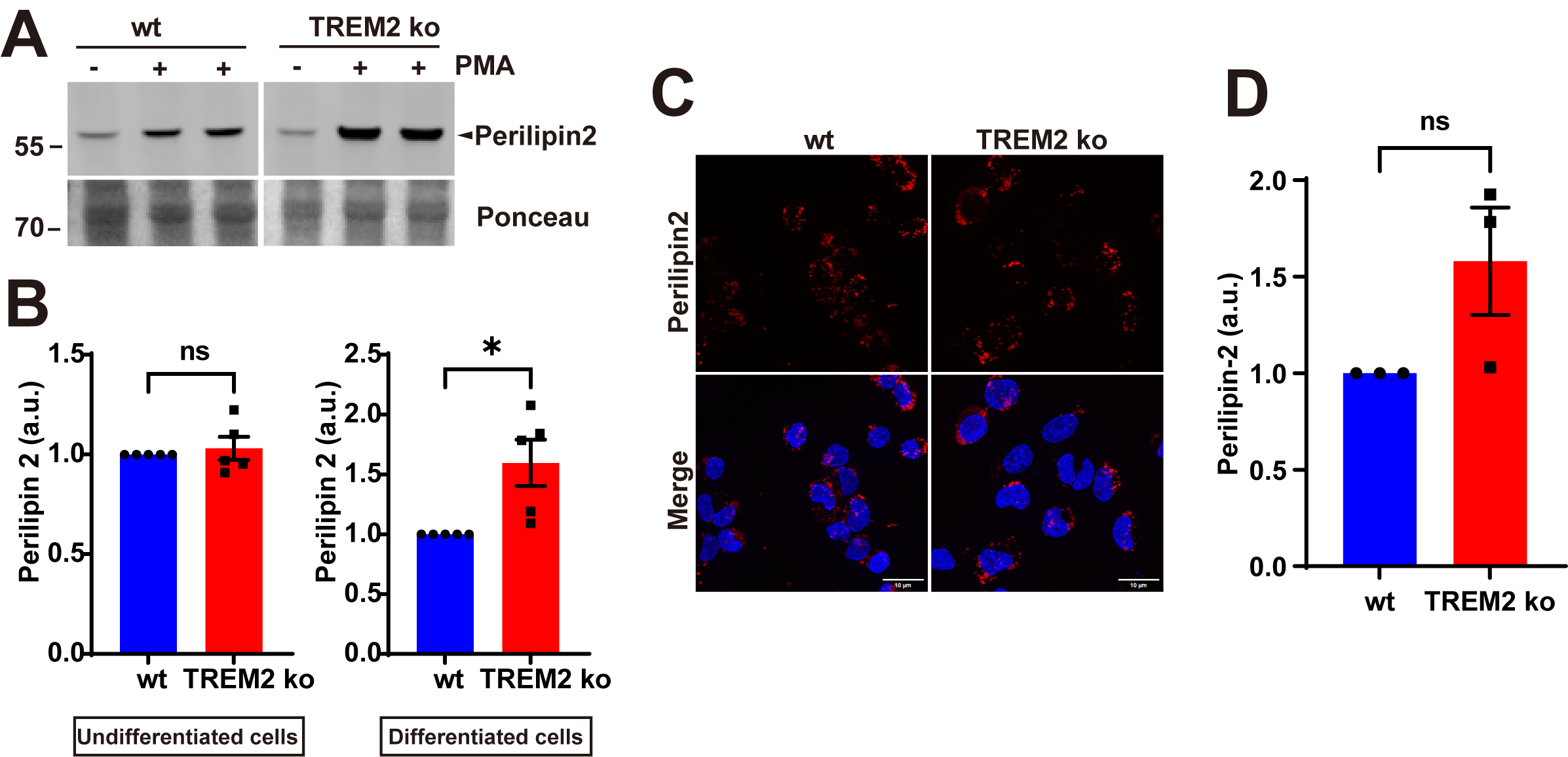

Supplement: Supplementary file 19 — Comparison of perilipin 2 expression level in TREM2 ko and wt THP-1 differentiated cells. (A) Detection of perilipin 2 in TREM2 ko and wt THP-1 differentiated cells. Cellular membranes were isolated and western immunoblotting was used for the detection of perilipin 2. (B) Quantification of perilipin 2 by western immunoblotting (as shown in A). Perilipin 2 was normalized to the full protein stained by Ponceau. Data represent mean ± SEM of five independent experiments with one to three samples per experiment. (C) Comparison of perilipin 2 in TREM2 ko and wt THP-1 differentiated cells by immunocytochemistry. Representative images are shown. Cells were co-stained with the perilipin 2 (red) and DAPI (blue). Scale bar = 10μm. (D) Quantification of perilipin 2 intensity shown. Each data point represents the mean value of an individual experiment. Student’s t-test (unpaired, two-tailed). *p < 0.05. [file 18_2025_5817_Fig18_ESM.png]

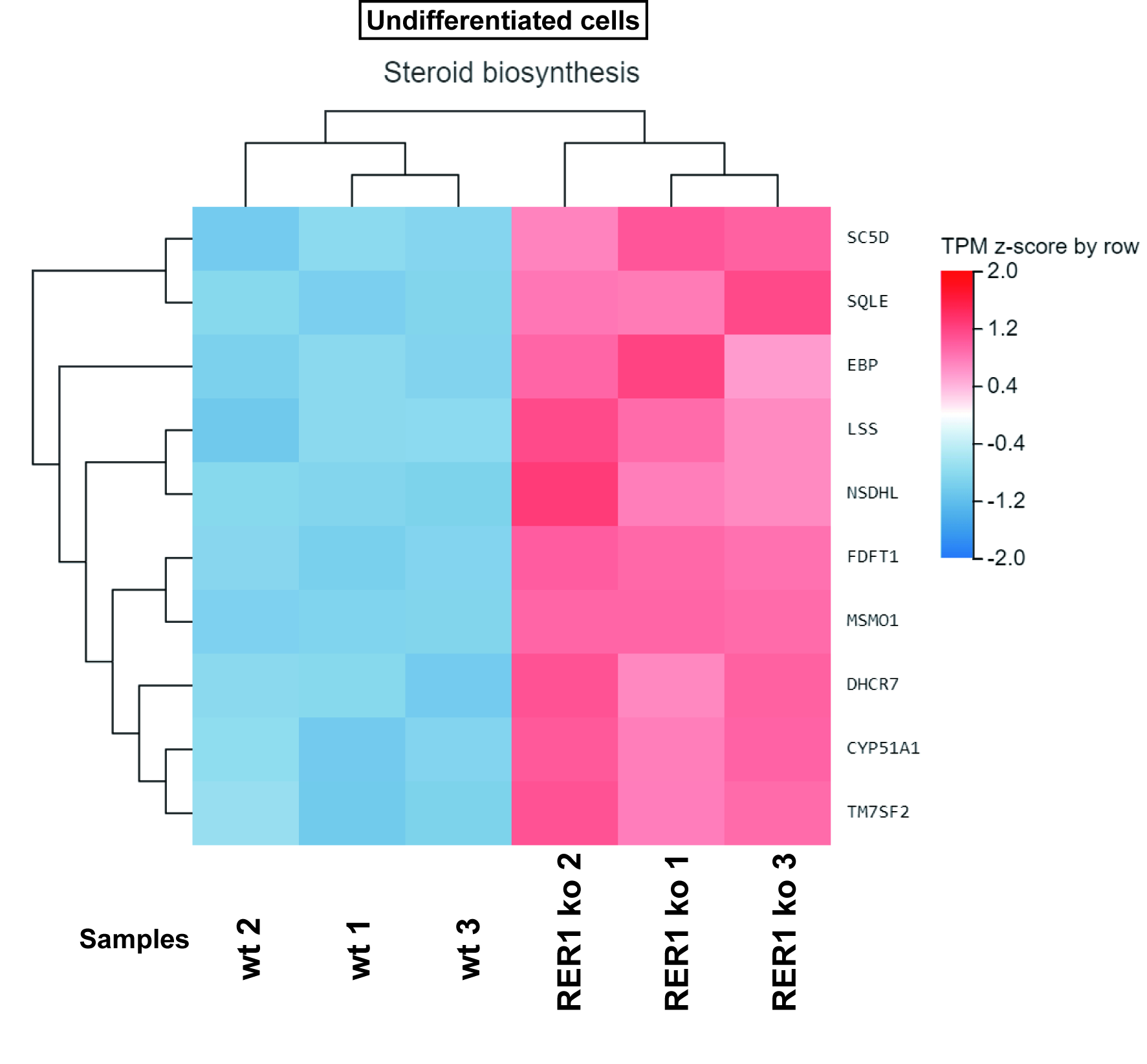

Supplement: Supplementary file 20 — High Resolution Image (TIF 8970 kb) [file 18_2025_5817_MOESM20_ESM.tif]

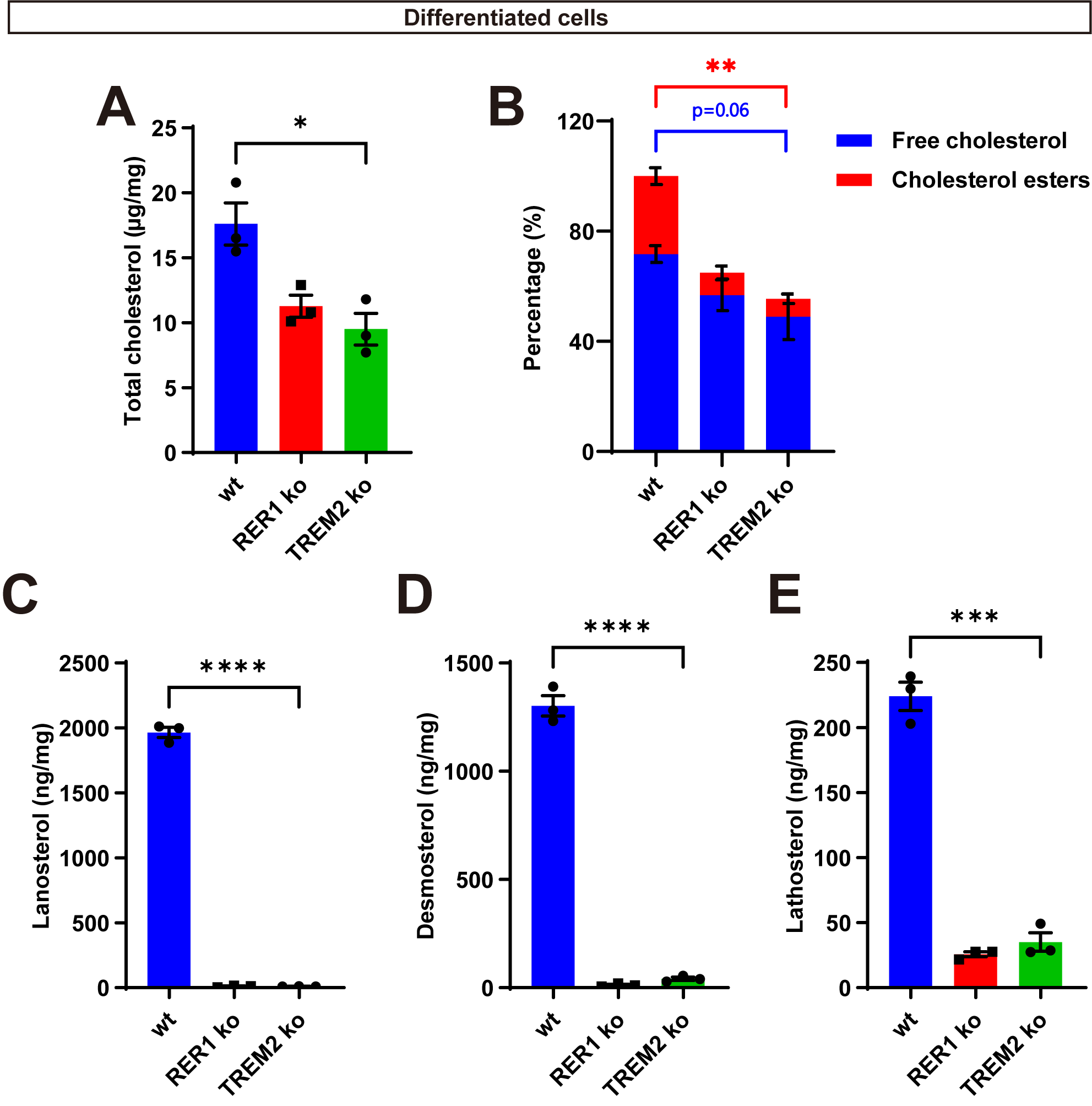

Supplement: Supplementary file 21 — Content analysis of a panel of sterols in TREM2 ko and wt THP-1 differentiated cells, determined by GC-FID and GC-MS-SIM. Free cholesterol and cholesteryl esters are expressed as a percentage of total cholesterol. Total cholesterol (A, absolute amount), free cholesterol and cholesterol esters (B, % of total cholesterol) as well as cholesterol precursors (lanosterol (C), desmosterol (D), lathosterol (E) were analyzed in THP-1 differentiated cells. Values represent mean ± SEM of three independent experiments each performed with triplicate samples. Each data point represents the mean value of an individual experiment. Student’s t-test (unpaired, two-tailed). *p < 0.05, **p < 0.01, ***p < 0.001, ****p < 0.0001. [file 18_2025_5817_Fig19_ESM.png]

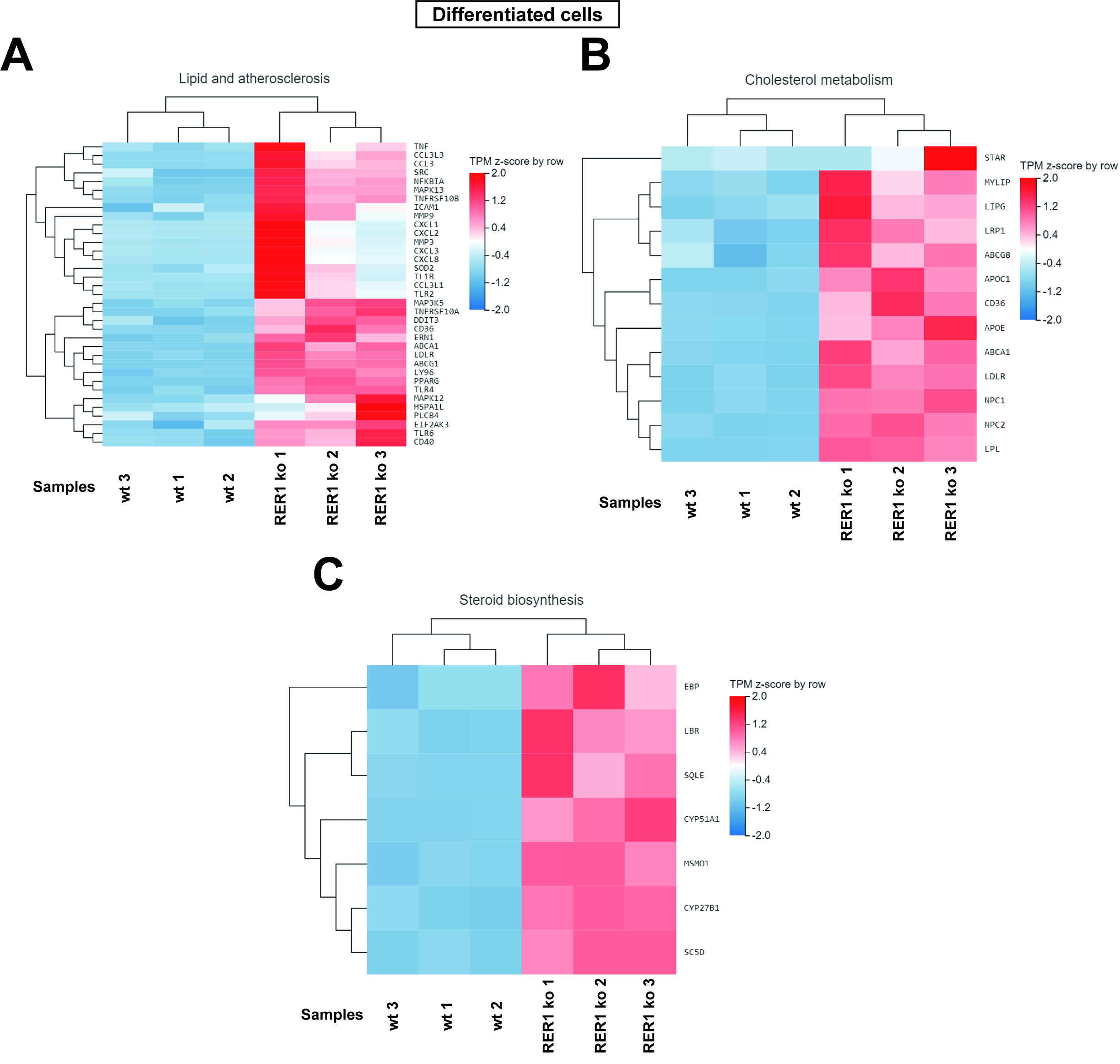

Supplement: Supplementary file 22 — High Resolution Image (TIF 8970 kb) [file 18_2025_5817_MOESM22_ESM.tif]

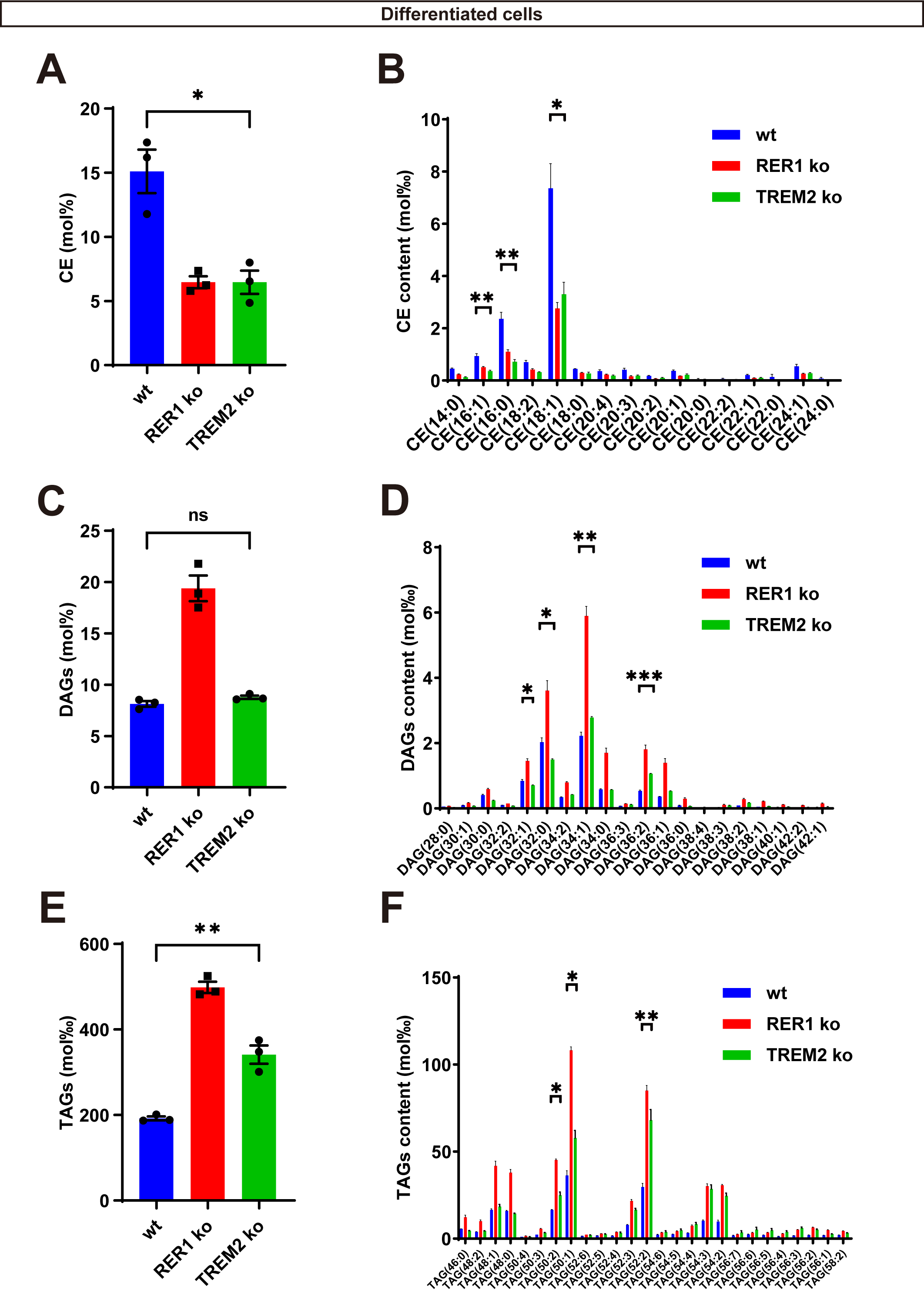

Supplement: Supplementary file 23 — Analysis of cholesterol esters (CEs) species, diacylglycerol (DAGs) species and triacylglycerol (TAGs) species by tandem mass spectrometry in TREM2 ko and wt differentiated cells. Total CEs (A) and different species (B), total diacylglycerol (C) and different species (D), total triacylglycerol (E) and different species (F) were analyzed in TREM2 ko and wt THP-1 differentiated cells. Values represent mean ± SEM of three independent experiments each performed with triplicate samples. Each data point represents the mean value of an individual replicate. Student’s t-test (unpaired, two-tailed). **p < 0.01. ***p < 0.001, ****p < 0.0001. [file 18_2025_5817_Fig20_ESM.png]

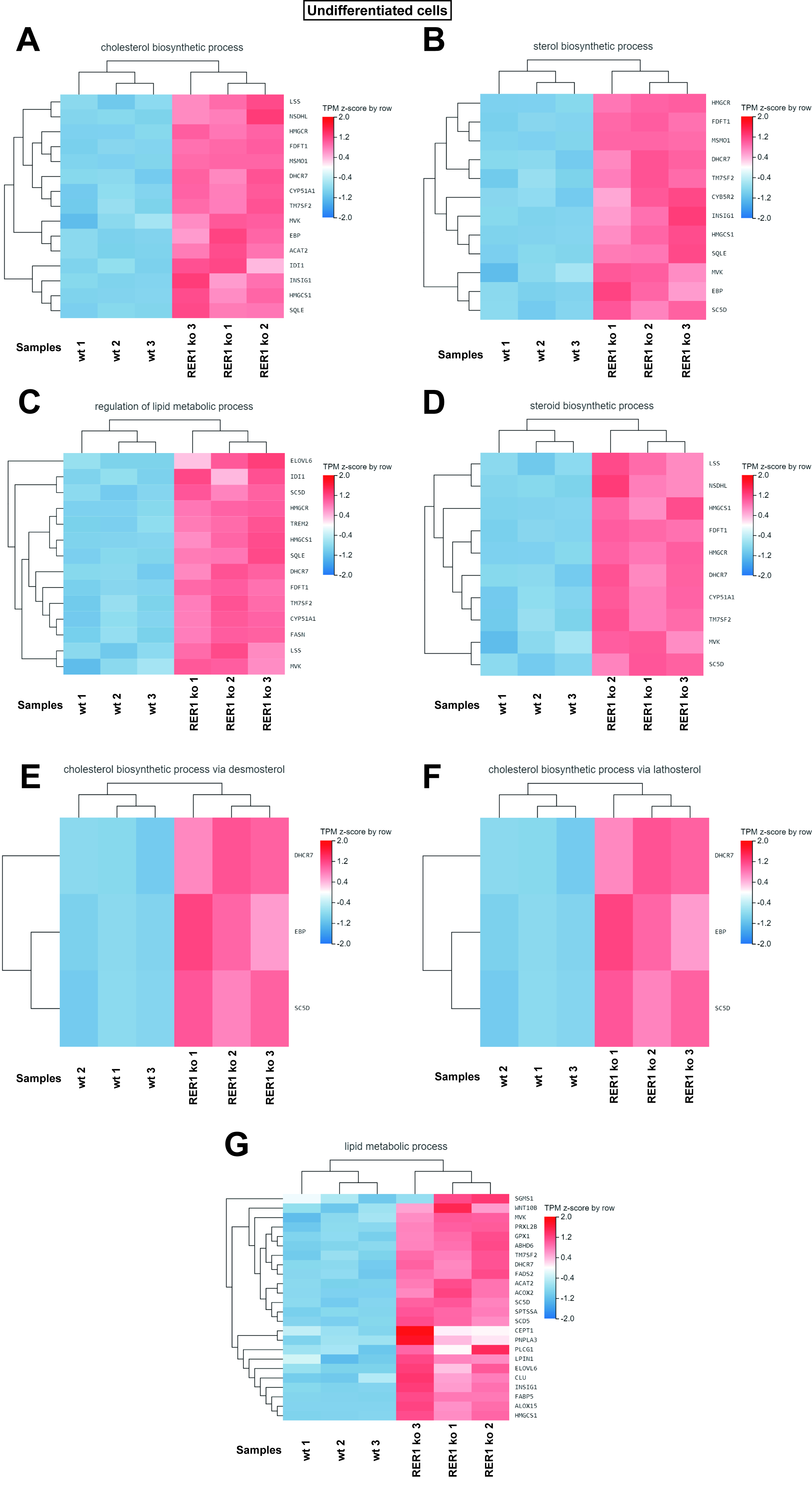

Supplement: Supplementary file 24 — High Resolution Image (TIF 8970 kb) [file 18_2025_5817_MOESM24_ESM.tif]

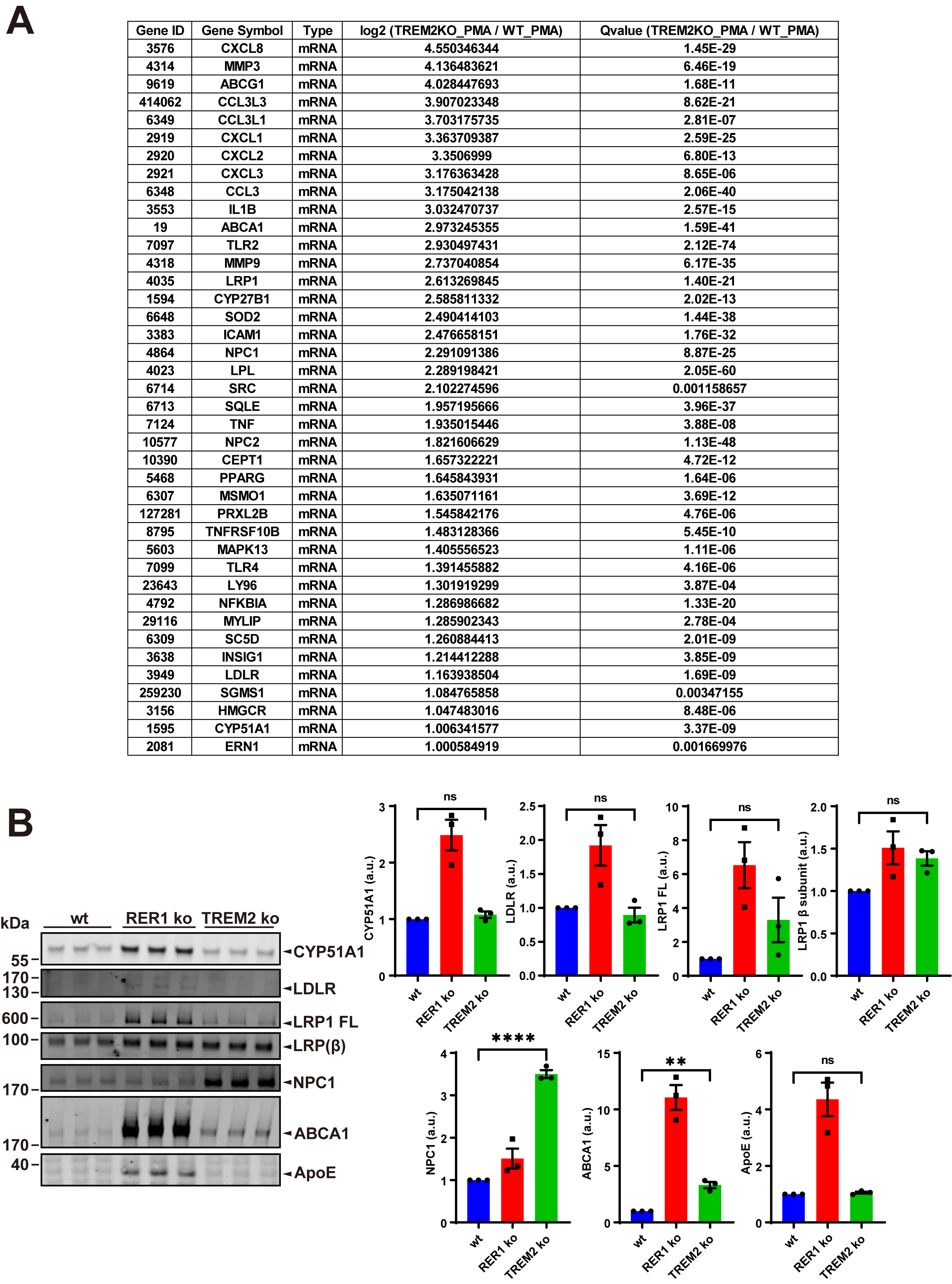

Supplement: Supplementary file 25 — The upregulated lipid related genes and proteins in TREM2 ko differentiated cells as compared to wt cells. (A) The overlapping upregulated genes that are related to lipid metabolism between RER1 ko and TREM2 ko THP-1 cells as compared to THP-1 wt cells. (B) Expressions of lipid metabolism related proteins in wt and TREM2 ko THP-1 differentiated cells. Cellular membranes were isolated, and western immunoblotting was used for the detection of the indicated protein. Quantification of CYP51A1, LDLR, LRP1, NPC1, ABCA1 and ApoE by western immunoblotting. Indicated proteins were normalized to the full protein stained by ponceau. Values represent mean ± SEM of three independent experiments with one to three samples per experiment. Each data point represents the mean value of an individual experiment. Student’s t-test (unpaired, two-tailed). **p < 0.01, ****p < 0.0001. [file 18_2025_5817_Fig21_ESM.png]

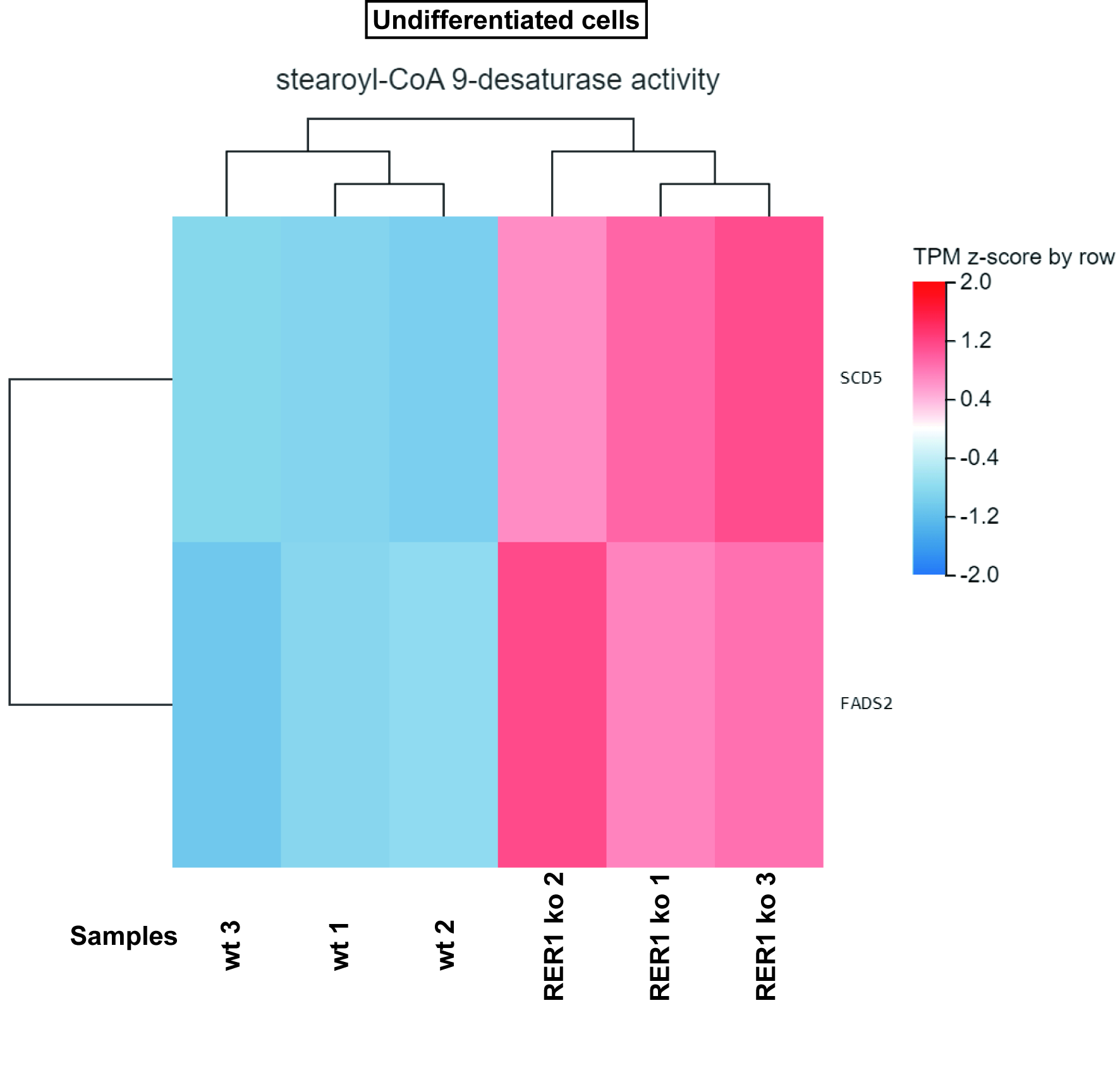

Supplement: Supplementary file 26 — High Resolution Image (TIF 8970 kb) [file 18_2025_5817_MOESM26_ESM.tif]

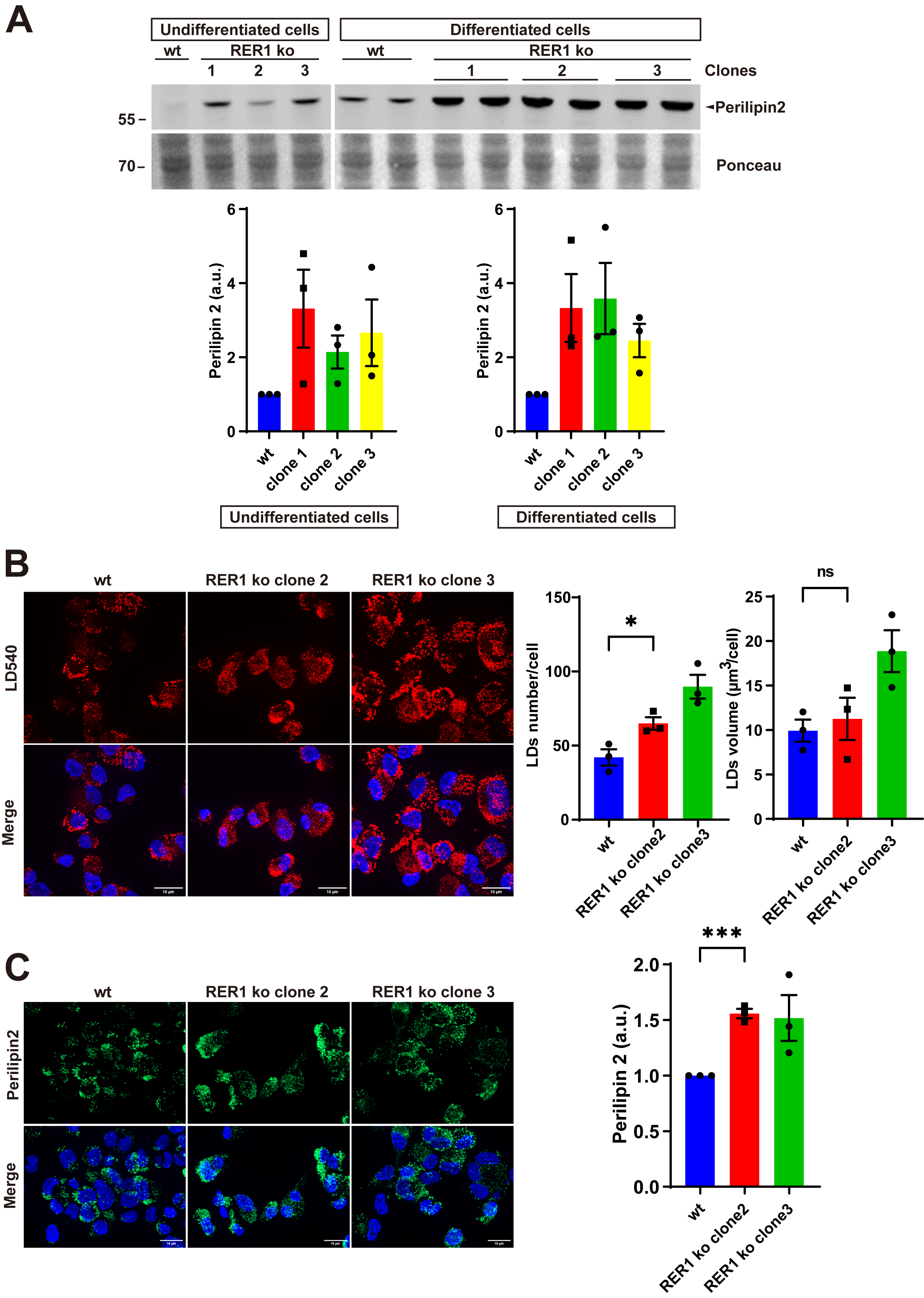

Supplement: Supplementary file 27 — Accumulation of lipid droplets (LDs) in different RER1 ko clones. (A) Detection of perilipin 2 in three different RER1 ko clones and wt THP-1 undifferentiated and differentiated cells. Cellular membranes were isolated and western immunoblotting was used for the detection of the indicated protein (clone 1: R1A7, clone 2: R2A10, clone 3: R1C7 (which was used for all other experiments)). (B) Comparison of LDs in different RER1 ko clones and wt THP-1 differentiated cells by LD540 staining. Representative images are shown. Cells were co-stained with LD540 (red) and DAPI (blue) to visualize LDs and nuclei, respectively. Scale bar = 10μm. LDs numbers and volume per cell were quantified by automated LD quantification (ALDQ) method. (C) Comparison of perilipin 2 in RER1 ko and wt THP-1 differentiated cells by immunocytochemistry. Representative images are shown. Cells were co-stained with the perilipin 2 (red) and DAPI (blue). Scale bar = 10μm. [file 18_2025_5817_Fig22_ESM.png]

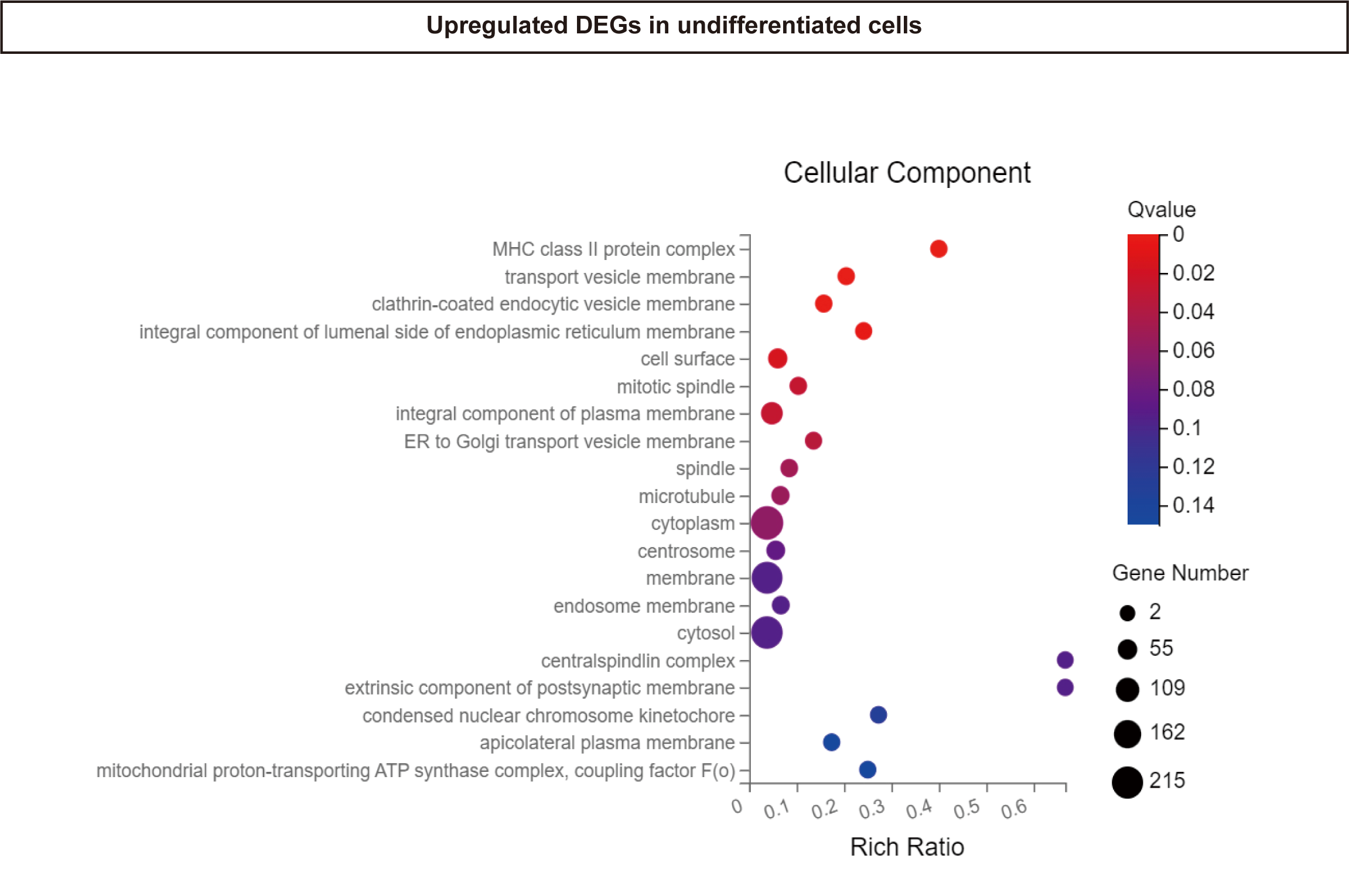

Supplement: Supplementary file 28 — High Resolution Image (TIF 8970 kb) [file 18_2025_5817_MOESM28_ESM.tif]
