# Supplementary material for: RER1 regulates lipid metabolism in monocytes and macrophages
Source: Cell Mol Life Sci. 2025 Aug 13;82(1):313. doi: 10.1007/s00018-025-05817-3 (PMC12351005; doi:10.1007/s00018-025-05817-3)
Supplement: Supplementary file 29 — Supplementary Material 15 [file 18_2025_5817_MOESM29_ESM.docx]

| Gene ID | Gene Symbol | Gene Name | Protein Name | Log2FoldChange  (RER1KO_noPMA / WT_noPMA) | Q value (adjust p value)  (RER1KO_noPMA / WT_noPMA) |
| --- | --- | --- | --- | --- | --- |
| 6307 | MSMO1 | Methylsterol monooxygenase 1 | Methylsterol monooxygenase 1 | 2.0842667 | 7.25E-50 |
| 6713 | SQLE | Squalene epoxidase | Squalene monooxygenase | 1.882558907 | 4.64E-72 |
| 10682 | EBP | EBP cholestenol delta-isomerase | 3-beta-hydroxysteroid-Delta(8),Delta(7)-isomerase | 1.731975796 | 3.91E-29 |
| 1595 | CYP51A1 | Cytochrome P450 family 51 subfamily A member 1 | Lanosterol 14-alpha demethylase | 1.409495453 | 9.27E-39 |
| 50814 | NSDHL | NAD(P) dependent steroid dehydrogenase-like | Sterol-4-alpha-carboxylate 3-dehydrogenase, decarboxylating | 1.37597262 | 7.50E-13 |
| 1717 | DHCR7 | 7-dehydrocholesterol reductase | 7-dehydrocholesterol reductase | 1.323759153 | 2.29E-14 |
| 6309 | SC5D | Sterol-C5-desaturase | Lathosterol oxidase | 1.246517242 | 8.83E-16 |
| 4047 | LSS | Lanosterol synthase | Lanosterol synthase | 1.185656272 | 3.53E-07 |
| 2222 | FDFT1 | Farnesyl-diphosphate farnesyltransferase 1 | Squalene synthase | 1.167520997 | 1.06E-35 |
| 7108 | TM7SF2 | Transmembrane 7 superfamily member 2 | Delta(14)-sterol reductase TM7SF2 | 1.07297873 | 5.41E-07 |

**Suppl. Table 1: Upregulation of steroid biosynthesis in RER1 ko undifferentiated THP-1 cells**

**Suppl. Table 2: Upregulation of cholesterol biosynthetic process in RER1 ko undifferentiated THP-1 cells**

| Gene ID | Gene Symbol | Gene Name | Protein Name | Log2FoldChange  (RER1KO_noPMA / WT_noPMA) | Qvalue (adjust p value)  (RER1KO_noPMA / WT_noPMA) |
| --- | --- | --- | --- | --- | --- |
| 6307 | MSMO1 | Methylsterol monooxygenase 1 | Methylsterol monooxygenase 1 | 2.0842667 | 7.25E-50 |
| 39 | ACAT2 | Acetyl-CoA acetyltransferase 2 | Acetyl-CoA acetyltransferase, cytosolic | 2.007003217 | 3.79E-55 |
| 3156 | HMGCR | 3-hydroxy-3-methylglutaryl-CoA reductase | 3-hydroxy-3-methylglutaryl-CoA reductase | 1.931107601 | 9.83E-61 |
| 6713 | SQLE | Squalene epoxidase | Squalene monooxygenase | 1.882558907 | 4.64E-72 |
| 10682 | EBP | EBP cholestenol delta-isomerase | 3-beta-hydroxysteroid-Delta(8), Delta(7)-isomerase | 1.731975796 | 3.91E-29 |
| 3638 | INSIG1 | insulin induced gene 1, CL6 | Insulin-induced gene 1 protein | 1.644743067 | 4.99E-57 |
| 1595 | CYP51A1 | Cytochrome P450 family 51 subfamily A member 1 | Lanosterol 14-alpha demethylase | 1.409495453 | 9.27E-39 |
| 50814 | NSDHL | NAD(P) dependent steroid dehydrogenase-like | Sterol-4-alpha-carboxylate 3-dehydrogenase, decarboxylating | 1.37597262 | 7.50E-13 |
| 1717 | DHCR7 | 7-dehydrocholesterol reductase, SLOS | 7-dehydrocholesterol reductase | 1.323759153 | 2.29E-14 |
| 3157 | HMGCS1 | 3-hydroxy-3-methylglutaryl-CoA synthase 1 | Hydroxymethylglutaryl-CoA synthase, cytoplasmic | 1.285537157 | 1.32E-42 |
| 4047 | LSS | Lanosterol synthase | Lanosterol synthase | 1.185656272 | 3.53E-07 |
| 2222 | FDFT1 | Farnesyl-diphosphate farnesyltransferase 1 | Squalene synthase | 1.167520997 | 1.06E-35 |
| 4598 | MVK | Mevalonate kinase | Mevalonate kinase | 1.146477972 | 1.34E-05 |
| 7108 | TM7SF2 | Transmembrane 7 superfamily member 2 | Delta(14)-sterol reductase TM7SF2 | 1.07297873 | 5.41E-07 |
| 3422 | IDI1 | Isopentenyl-diphosphate delta isomerase 1 | Isopentenyl-diphosphate Delta-isomerase 1 | 1.010133383 | 1.32E-10 |

**Suppl. Table 3: Upregulation of sterol biosynthetic process in RER1 ko undifferentiated cells**

| Gene ID | Gene Symbol | Gene Name | Protein Name | Log2FoldChange  (RER1KO_noPMA / WT_noPMA) | Qvalue (adjust p value)  (RER1KO_noPMA / WT_noPMA) |
| --- | --- | --- | --- | --- | --- |
| 6307 | MSMO1 | Methylsterol monooxygenase 1 | Methylsterol monooxygenase 1 | 2.0842667 | 7.25E-50 |
| 3156 | HMGCR | 3-hydroxy-3-methylglutaryl-CoA reductase | 3-hydroxy-3-methylglutaryl-CoA reductase | 1.931107601 | 9.83E-61 |
| 6713 | SQLE | Squalene epoxidase | Squalene monooxygenase | 1.882558907 | 4.64E-72 |
| 10682 | EBP | EBP cholestenol delta-isomerase | 3-beta-hydroxysteroid-Delta(8),Delta(7)-isomerase | 1.731975796 | 3.91E-29 |
| 3638 | INSIG1 | Insulin induced gene 1 | Insulin-induced gene 1 protein | 1.644743067 | 4.99E-57 |
| 51700 | CYB5R2 | Cytochrome b5 reductase 2 | NADH-cytochrome b5 reductase 2 | 1.362439787 | 3.00E-06 |
| 1717 | DHCR7 | 7-dehydrocholesterol reductase | 7-dehydrocholesterol reductase | 1.323759153 | 2.29E-14 |
| 3157 | HMGCS1 | 3-hydroxy-3-methylglutaryl-CoA synthase 1 | Hydroxymethylglutaryl-CoA synthase, cytoplasmic | 1.285537157 | 1.32E-42 |
| 6309 | SC5D | Sterol-C5-desaturase, ERG3, S5DES | Lathosterol oxidase | 1.246517242 | 8.83E-16 |
| 2222 | FDFT1 | Farnesyl-diphosphate farnesyltransferase 1 | Squalene synthase | 1.167520997 | 1.06E-35 |
| 4598 | MVK | Mevalonate kinase | Mevalonate kinase | 1.146477972 | 1.34E-05 |
| 7108 | TM7SF2 | Transmembrane 7 superfamily member 2 | Delta(14)-sterol reductase TM7SF2 | 1.07297873 | 5.41E-07 |

**Suppl. Table 4: Regulation of lipid metabolic process is upregulated in RER1 ko undifferentiated cells**

| Gene ID | Gene Symbol | Gene Name | Protein Name | Log2FoldChange  (RER1KO_noPMA / WT_noPMA) | Qvalue (adjust p value)  (RER1KO_noPMA / WT_noPMA) |
| --- | --- | --- | --- | --- | --- |
| 79071 | ELOVL6 | ELOVL fatty acid elongase 6 | Very long chain fatty acid elongase 6 | 5.02457542 | 0.001995805 |
| 3156 | HMGCR | 3-hydroxy-3-methylglutaryl-CoA reductase | 3-hydroxy-3-methylglutaryl-CoA reductase | 1.931107601 | 9.83E-61 |
| 6713 | SQLE | squalene epoxidase | Squalene monooxygenase | 1.882558907 | 4.64E-72 |
| 1595 | CYP51A1 | cytochrome P450 family 51 subfamily A member 1 | Lanosterol 14-alpha demethylase | 1.409495453 | 9.27E-39 |
| 1717 | DHCR7 | 7-dehydrocholesterol reductase | 7-dehydrocholesterol reductase | 1.323759153 | 2.29E-14 |
| 3157 | HMGCS1 | 3-hydroxy-3-methylglutaryl-CoA synthase 1 | Hydroxymethylglutaryl-CoA synthase, cytoplasmic | 1.285537157 | 1.32E-42 |
| 2194 | FASN | fatty acid synthase | Fatty acid synthase | 1.251084436 | 4.21E-36 |
| 6309 | SC5D | sterol-C5-desaturase | Lathosterol oxidase | 1.246517242 | 8.83E-16 |
| 4047 | LSS | lanosterol synthase | Lanosterol synthase | 1.185656272 | 3.53E-07 |
| 2222 | FDFT1 | farnesyl-diphosphate farnesyltransferase 1 | Squalene synthase | 1.167520997 | 1.06E-35 |
| 4598 | MVK | mevalonate kinase | Mevalonate kinase | 1.146477972 | 1.34E-05 |
| 54209 | TREM2 | Triggering receptor expressed on myeloid cells 2 |  | 1.129664455 | 3.98E-11 |
| 7108 | TM7SF2 | transmembrane 7 superfamily member 2 | Delta(14)-sterol reductase TM7SF2 | 1.07297873 | 5.41E-07 |
| 3422 | IDI1 | isopentenyl-diphosphate delta isomerase 1 | Isopentenyl-diphosphate Delta-isomerase 1 | 1.010133383 | 1.32E-10 |

**Suppl. Table 5: The steroid biosynthetic process is upregulated in RER1 ko undifferentiated THP-1 cells**

| Gene ID | Gene Symbol | Gene Name | Protein Name | Log2FoldChange  (RER1KO_noPMA / WT_noPMA) | Qvalue (adjust p value)  (RER1KO_noPMA / WT_noPMA) |
| --- | --- | --- | --- | --- | --- |
| 3156 | HMGCR | 3-hydroxy-3-methylglutaryl-CoA reductase | 3-hydroxy-3-methylglutaryl-CoA reductase | 1.931107601 | 9.83E-61 |
| 1595 | CYP51A1 | Cytochrome P450 family 51 subfamily A member 1 | Lanosterol 14-alpha demethylase | 1.409495453 | 9.27E-39 |
| 50814 | NSDHL | NAD(P) dependent steroid dehydrogenase-like | Sterol-4-alpha-carboxylate 3-dehydrogenase, decarboxylating | 1.37597262 | 7.50E-13 |
| 1717 | DHCR7 | 7-dehydrocholesterol reductase | 7-dehydrocholesterol reductase | 1.323759153 | 2.29E-14 |
| 3157 | HMGCS1 | 3-hydroxy-3-methylglutaryl-CoA synthase 1 | Lanosterol 14-alpha demethylase | 1.285537157 | 1.32E-42 |
| 6309 | SC5D | Sterol-C5-desaturase | Lathosterol oxidase | 1.246517242 | 8.83E-16 |
| 4047 | LSS | Lanosterol synthase | Lanosterol synthase | 1.185656272 | 3.53E-07 |
| 2222 | FDFT1 | Farnesyl-diphosphate farnesyltransferase 1 | Squalene synthase | 1.167520997 | 1.06E-35 |
| 4598 | MVK | Mevalonate kinase | Mevalonate kinase | 1.146477972 | 1.34E-05 |
| 7108 | TM7SF2 | Transmembrane 7 superfamily member 2 | Delta(14)-sterol reductase TM7SF2 | 1.07297873 | 5.41E-07 |

**Suppl. Table 6: Cholesterol biosynthetic process via desmosterol and lathosterol is upregulated in RER1 ko undifferentiated cells**

| Gene ID | Gene Symbol | Gene Name | Protein Name | Log2FoldChange  (RER1KO_noPMA / WT_noPMA) | Qvalue (adjust p value)  (RER1KO_noPMA / WT_noPMA) |
| --- | --- | --- | --- | --- | --- |
| 10682 | EBP | EBP cholestenol delta-isomerase | 3-beta-hydroxysteroid-Delta(8),Delta(7)-isomerase | 1.731975796 | 3.91E-29 |
| 1717 | DHCR7 | 7-dehydrocholesterol reductase | 7-dehydrocholesterol reductase | 1.323759153 | 2.29E-14 |
| 6309 | SC5D | Sterol-C5-desaturase | Lathosterol oxidase | 1.246517242 | 8.83E-16 |

**Suppl. Table 7: Lipid metabolic process is upregulated in RER1 ko undifferentiated THP-1 cells**

| Gene ID | Gene Symbol | Gene Name | Protein Name | Log2FoldChange  (RER1KO_noPMA / WT_noPMA) | Qvalue (adjust p value)  (RER1KO_noPMA / WT_noPMA) |
| --- | --- | --- | --- | --- | --- |
| 8309 | ACOX2 | Acyl-CoA oxidase 2 | Peroxisomal acyl-coenzyme A oxidase 2 | 5.40578578 | 4.58E-04 |
| 246 | ALOX15 | Arachidonate 15-lipoxygenase | Polyunsaturated fatty acid lipoxygenase ALOX15 | 5.134196348 | 0.005724019 |
| 79071 | ELOVL6 | ELOVL fatty acid elongase 6 | Very long chain fatty acid elongase 6 | 5.02457542 | 0.001995805 |
| 79966 | SCD5 | Stearoyl-CoA desaturase 5 | Stearoyl-CoA desaturase 5 | 2.780915058 | 9.74E-23 |
| 7480 | WNT10B | Wnt family member 10B | Protein Wnt-10b | 2.489258935 | 0.01048109 |
| 39 | ACAT2 | Acetyl-CoA acetyltransferase 2 | acetyl-CoA acetyltransferase 2 | 2.007003217 | 3.79E-55 |
| 127281 | PRXL2B | Peroxiredoxin like 2B | Prostamide/prostaglandin F synthase | 1.941103681 | 1.13E-08 |
| 3638 | INSIG1 | Insulin induced gene 1 | Insulin-induced gene 1 protein | 1.644743067 | 4.99E-57 |
| 5335 | PLCG1 | Phospholipase C gamma 1 | 1-phosphatidylinositol 4,5-bisphosphate phosphodiesterase gamma-1 | 1.524097591 | 1.24E-04 |
| 2876 | GPX1 | Glutathione peroxidase 1 | Glutathione peroxidase 1 | 1.413364161 | 6.91E-42 |
| 259230 | SGMS1 | Sphingomyelin synthase 1 | Phosphatidylcholine:ceramide cholinephosphotransferase 1 | 1.389932176 | 0.033195848 |
| 57406 | ABHD6 | Abhydrolase domain containing 6, acylglycerol lipase | Monoacylglycerol lipase ABHD6 | 1.356112209 | 0.004410288 |
| 1717 | DHCR7 | 7-dehydrocholesterol reductase | 7-dehydrocholesterol reductase | 1.323759153 | 2.29E-14 |
| 1191 | CLU | Clusterin | Clusterin | 1.300241265 | 0.017307615 |
| 3157 | HMGCS1 | 3-hydroxy-3-methylglutaryl-CoA synthase 1 | Lanosterol 14-alpha demethylase | 1.285537157 | 1.32E-42 |
| 6309 | SC5D | Sterol-C5-desaturase | Lathosterol oxidase | 1.246517242 | 8.83E-16 |
| 9415 | FADS2 | Fatty acid desaturase 2 | Acyl-CoA 6-desaturase | 1.171429157 | 1.01E-15 |
| 2171 | FABP5 | Fatty acid binding protein 5 | Fatty acid-binding protein 5 | 1.159770456 | 1.01E-33 |
| 80339 | PNPLA3 | Patatin like phospholipase domain containing 3 | 1-acylglycerol-3-phosphate O-acyltransferase PNPLA3 | 1.156253221 | 1.27E-07 |
| 4598 | MVK | Mevalonate kinase | Mevalonate kinase | 1.146477972 | 1.34E-05 |
| 7108 | TM7SF2 | Transmembrane 7 superfamily member 2 | Delta(14)-sterol reductase TM7SF2 | 1.07297873 | 5.41E-07 |
| 171546 | SPTSSA | Serine palmitoyltransferase small subunit A | Serine palmitoyltransferase small subunit A | 1.033638595 | 1.22E-13 |
| 23175 | LPIN1 | Lipin 1 | Phosphatidate phosphatase LPIN1 | 1.009996073 | 2.87E-05 |
| 10390 | CEPT1 | Choline/ethanolamine phosphotransferase 1 | Choline/ethanolaminephosphotransferase 1 | 1.003102894 | 8.06E-07 |

**Suppl. Table 8: The stearoyl-coa 9-desaturase activity is upregulated in RER1 ko undifferentiated THP-1 cells**

| Gene ID | Gene Symbol | Gene Name | Protein Name | Log2FoldChange  (RER1KO_noPMA / WT_noPMA) | Qvalue (adjust p value)  (RER1KO_noPMA / WT_noPMA) |
| --- | --- | --- | --- | --- | --- |
| 79966 | SCD5 | Stearoyl-CoA desaturase 5 | Stearoyl-CoA desaturase 5 | 2.780915058 | 9.74E-23 |
| 9415 | FADS2 | Fatty acid desaturase 2 | Acyl-CoA 6-desaturase | 1.171429157 | 1.01E-15 |

**Suppl. Table 9: The** **lipid and atherosclerosis are upregulated in RER1 ko THP-1 differentiated cells**

| Gene ID | Gene Symbol | Gene Name | Protein Name | Log2FoldChange  (RER1KO_noPMA / WT_noPMA) | Qvalue (adjust p value)  (RER1KO_noPMA / WT_noPMA) |
| --- | --- | --- | --- | --- | --- |
| 3576 | CXCL8 | GCP-1 | Interleukin-8 | 6.066621544 | 7.57E-10 |
| 9619 | ABCG1 | ATP-binding Cassette Sub-family G Member 1 | ATP-binding cassette sub-family G member 1 | 5.968989539 | 7.54E-36 |
| 2921 | CXCL3 | CINC-2b | C-X-C motif chemokine 3 | 5.074361946 | 1.14E-05 |
| 2920 | CXCL2 | CINC-2a | C-X-C motif chemokine 2 | 4.905915124 | 2.59E-05 |
| 2919 | CXCL1 | FSP | Growth-regulated alpha protein | 4.893429044 | 7.38E-06 |
| 414062 | CCL3L3 | D17S1718 | C-C motif chemokine 3-like 1 | 4.689453864 | 3.39E-18 |
| 6348 | CCL3 | G0S19-1 | Acetate--CoA ligase CCL3 | 4.376888214 | 1.50E-19 |
| 6349 | CCL3L1 | D17S1718 | C-C motif chemokine 3-like 1 | 4.207976495 | 5.43E-06 |
| 19 | ABCA1 | ATP binding cassette subfamily A member 1 | Phospholipid-transporting ATPase ABCA1 | 3.934920512 | 1.87E-70 |
| 3553 | IL1B | IL-1 | Interleukin-1 beta | 3.655275635 | 1.66E-05 |
| 4314 | MMP3 | Matrix metallopeptidase 3 | Stromelysin-1 | 3.647444557 | 1.00E-07 |
| 7124 | TNF | Tumor necrosis factor | Tumor necrosis factor | 2.751193753 | 1.79E-04 |
| 5468 | PPARG | Peroxisome proliferator activated receptor gamma | Peroxisome proliferator-activated receptor gamma | 2.627009807 | 1.00E-20 |
| 5603 | MAPK13 | Mitogen-activated protein kinase 13 | Mitogen-activated protein kinase 13 | 2.322800062 | 3.24E-15 |
| 7099 | TLR4 | Toll like receptor 4 | Toll-like receptor 4 | 2.306476019 | 4.30E-18 |
| 8797 | TNFRSF10A | TNF receptor superfamily member 10a | Tumor necrosis factor receptor superfamily member 10A | 2.136574286 | 4.21E-22 |
| 948 | CD36 | CD36 molecule (CD36 blood group) | Platelet glycoprotein 4 | 2.128277266 | 3.03E-26 |
| 3949 | LDLR | Low density lipoprotein receptor | Low-density lipoprotein receptor | 2.105997122 | 4.50E-29 |
| 23643 | LY96 | Lymphocyte antigen 96 | Lymphocyte antigen 96 | 1.987456241 | 3.87E-11 |
| 6714 | SRC | SRC proto-oncogene, non-receptor tyrosine kinase | Proto-oncogene tyrosine-protein kinase Src | 1.893996985 | 0.004275188 |
| 7097 | TLR2 | Toll like receptor 2 | Toll-like receptor 2 | 1.780798796 | 2.69E-08 |
| 6648 | SOD2 | Superoxide dismutase 2 | Superoxide dismutase [Mn], mitochondrial | 1.762910654 | 0.006626211 |
| 8795 | TNFRSF10B | TNF receptor superfamily member 10b | Tumor necrosis factor receptor superfamily member 10B | 1.714914321 | 4.34E-10 |
| 4217 | MAP3K5 | Mitogen-activated protein kinase 5 | Mitogen-activated protein kinase kinase kinase 5 | 1.648072139 | 9.28E-11 |
| 5332 | PLCB4 | Phospholipase C beta 4 | 1-phosphatidylinositol 4,5-bisphosphate phosphodiesterase beta-4 | 1.435641161 | 4.88E-04 |
| 958 | CD40 | CD40 molecule | Tumor necrosis factor receptor superfamily member 5 | 1.420692755 | 1.87E-10 |
| 9451 | EIF2AK3 | Eukaryotic translation initiation factor 2 alpha kinase 3 | Eukaryotic translation initiation factor 2-alpha kinase 3 | 1.394181327 | 3.15E-04 |
| 3305 | HSPA1L | Heat shock protein family A (Hsp70) member 1 like | Heat shock 70 kDa protein 1-like | 1.376496254 | 0.044258948 |
| 4318 | MMP9 | Matrix metallopeptidase 9 | Matrix metalloproteinase-9 | 1.346834459 | 5.41E-07 |
| 1649 | DDIT3 | DNA damage inducible transcript 3 | DNA damage-inducible transcript 3 protein | 1.23301527 | 2.97E-05 |
| 2081 | ERN1 | Endoplasmic reticulum to nucleus signaling 1 | Serine/threonine-protein kinase/endoribonuclease IRE1 | 1.151335199 | 2.54E-04 |
| 10333 | TLR6 | Toll like receptor 6 | Toll-like receptor 6 | 1.140748871 | 7.37E-09 |
| 4792 | NFKBIA | NFKB inhibitor alpha | NF-kappa-B inhibitor alpha | 1.140159714 | 1.79E-08 |
| 3383 | ICAM1 | Intercellular adhesion molecule 1 | Intercellular adhesion molecule 1 | 1.072405047 | 7.40E-04 |
| 6300 | MAPK12 | Mitogen-activated protein kinase 12 | Mitogen-activated protein kinase 12 | 1.01158305 | 0.001110388 |

**Suppl. Table 10: Cholesterol metabolism is upregulated in RER1 ko differentiated THP-1 cells**

| Gene ID | Gene Symbol | Gene Name/ Alias | Protein Name | Log2FoldChange  (RER1KO_noPMA / WT_noPMA) | Qvalue (adjust p value)  (RER1KO_noPMA / WT_noPMA) |
| --- | --- | --- | --- | --- | --- |
| 19 | ABCA1 | ATP binding cassette subfamily A member 1 | Phospholipid-transporting ATPase ABCA1 | 3.934920512 | 1.87E-70 |
| 348 | APOE | Apolipoprotein E | Apolipoprotein E | 2.975272472 | 2.92E-48 |
| 4023 | LPL | Lipoprotein lipase | Lipoprotein lipase | 2.878286259 | 5.01E-77 |
| 4864 | NPC1 | NPC intracellular cholesterol transporter 1 | NPC intracellular cholesterol transporter 1 | 2.643585611 | 8.46E-37 |
| 341 | APOC1 | Apolipoprotein C1 | Apolipoprotein C-I | 2.487522836 | 1.12E-38 |
| 948 | CD36 | CD36 molecule (CD36 blood group) | Platelet glycoprotein 4 | 2.128277266 | 3.03E-26 |
| 3949 | LDLR | Low density lipoprotein receptor | Low-density lipoprotein receptor | 2.105997122 | 4.50E-29 |
| 6770 | STAR | Steroidogenic acute regulatory protein | Steroidogenic acute regulatory protein, mitochondrial | 2.095579028 | 0.022716988 |
| 4035 | LRP1 | LDL receptor related protein 1 | low-density lipoprotein receptor-related protein 1 | 2.071860819 | 2.19E-10 |
| 9388 | LIPG | Lipase G, endothelial type | Endothelial lipase | 1.721075307 | 2.82E-05 |
| 29116 | MYLIP | Myosin regulatory light chain interacting protein | E3 ubiquitin-protein ligase MYLIP | 1.535327645 | 2.21E-05 |
| 10577 | NPC2 | NPC intracellular cholesterol transporter 2 | NPC intracellular cholesterol transporter 2 | 1.247088724 | 5.21E-17 |
| 64241 | ABCG8 | ATP binding cassette subfamily G member 8 | ATP-binding cassette sub-family G member 8 | 1.111005225 | 0.031282198 |

**Suppl. Table 11: Steroid biosynthesis is upregulated in RER1 ko differentiated THP-1 cells**

| Gene ID | Gene Symbol | Gene Name | Protein Name | Log2FoldChange  (RER1KO_noPMA / WT_noPMA) | Qvalue (adjust p value)  (RER1KO_noPMA / WT_noPMA) |
| --- | --- | --- | --- | --- | --- |
| 6713 | SQLE | squalene epoxidase | Squalene monooxygenase | 2.520868079 | 1.01E-39 |
| 6307 | MSMO1 | methylsterol monooxygenase 1 | methylsterol monooxygenase 1 | 2.376808772 | 8.55E-38 |
| 6309 | SC5D | sterol-C5-desaturase | Lathosterol oxidase | 2.301355407 | 5.86E-34 |
| 1595 | CYP51A1 | cytochrome P450 family 51 subfamily A member 1 | Lanosterol 14-alpha demethylase | 2.108690047 | 4.35E-42 |
| 1594 | CYP27B1 | cytochrome P450 family 27 subfamily B member 1 | 25-hydroxyvitamin D-1 alpha hydroxylase, mitochondrial | 1.543361144 | 2.93E-04 |
| 10682 | EBP | EBP cholestenol delta-isomerase | 3-beta-hydroxysteroid-Delta(8),Delta(7)-isomerase | 1.359461691 | 3.81E-10 |
| 3930 | LBR | lamin B receptor | Delta(14)-sterol reductase LBR | 1.253023519 | 1.87E-11 |

**Suppl. Table:12: The TPM values of genes involved in the pathways mentioned in supplementary figures**

| Gene ID | Gene Symbol | WT_noPMA1 TPM | WT_noPMA2 TPM | WT_noPMA3 TPM | RER1KO_noPMA1 TPM | RER1KO_noPMA2 TPM | RER1KO_noPMA3 TPM |
| --- | --- | --- | --- | --- | --- | --- | --- |
| 19 | ABCA1 | 3.05 | 2.56 | 4.22 | 56.22 | 37.34 | 48.81 |
| 39 | ACAT2 | 67.72 | 53.86 | 58.74 | 253.33 | 226.11 | 219.61 |
| 246 | ALOX15 | 0 | 0 | 0 | 0.38 | 0.44 | 0.52 |
| 341 | APOC1 | 383.49 | 497.78 | 378.92 | 2069.56 | 2717.68 | 2012.66 |
| 348 | APOE | 62.95 | 50.56 | 71.63 | 345.91 | 438.51 | 617.74 |
| 948 | CD36 | 18.48 | 16.29 | 19.75 | 64.57 | 105.29 | 80.75 |
| 958 | CD40 | 47.57 | 33.96 | 49.87 | 109.16 | 99.12 | 151 |
| 1191 | CLU | 0.8 | 0.78 | 1.17 | 1.91 | 2.07 | 2.47 |
| 1594 | CYP27B1 | 2.19 | 2.09 | 2.49 | 6.04 | 6.44 | 6.31 |
| 1595 | CYP51A1 | 31.83 | 39.3 | 35.87 | 86.38 | 94.74 | 92.5 |
| 1649 | DDIT3 | 16.63 | 18.68 | 24.81 | 39.36 | 48.5 | 45.97 |
| 1717 | DHCR7 | 9.51 | 9.74 | 8.26 | 20.64 | 23.47 | 22.69 |
| 2081 | ERN1 | 1.82 | 2.03 | 1.71 | 3.97 | 4.42 | 3.34 |
| 2171 | FABP5 | 227.51 | 225.31 | 221.95 | 469.19 | 468.86 | 519.21 |
| 2194 | FASN | 21.41 | 25.98 | 22.06 | 50.65 | 54.59 | 54.14 |
| 2222 | FDFT1 | 156.19 | 170.55 | 165.13 | 354.56 | 363.49 | 347.64 |
| 2876 | GPX1 | 642.52 | 706.02 | 599.99 | 1652.2 | 1785.21 | 1550.66 |
| 2919 | CXCL1 | 2.74 | 4.84 | 8.12 | 317.12 | 64.24 | 41.81 |
| 2920 | CXCL2 | 1.09 | 1.77 | 3.66 | 127.25 | 26.2 | 15.14 |
| 2921 | CXCL3 | 2.64 | 3.7 | 12.17 | 414.22 | 85.18 | 65.36 |
| 3156 | HMGCR | 10.65 | 10.84 | 12.12 | 39.35 | 41.62 | 42.13 |
| 3157 | HMGCS1 | 25.36 | 25.66 | 26.41 | 56.04 | 61.1 | 64.95 |
| 3305 | HSPA1L | 1.11 | 1.36 | 0.92 | 1.47 | 2.06 | 4.89 |
| 3383 | ICAM1 | 13.93 | 10.7 | 8.08 | 28.04 | 20.59 | 16.67 |
| 3422 | IDI1 | 14.8 | 16.18 | 14.56 | 32.68 | 25.99 | 31.95 |
| 3553 | IL1B | 377.52 | 406.19 | 270.42 | 7956.79 | 2972.62 | 1352.09 |
| 3576 | CXCL8 | 47.4 | 71.83 | 109.93 | 9967.86 | 2212.53 | 1853.74 |
| 3638 | INSIG1 | 45.65 | 42.43 | 43.38 | 116.32 | 131.25 | 151.42 |
| 3930 | LBR | 43.44 | 44.19 | 48.56 | 118.24 | 96.49 | 92.72 |
| 3949 | LDLR | 22.44 | 17.37 | 17.4 | 86.1 | 71.87 | 76.47 |
| 4023 | LPL | 113.97 | 116.79 | 111 | 842.26 | 818.28 | 726.49 |
| 4035 | LRP1 | 0.73 | 0.94 | 1.65 | 5.48 | 4.26 | 3.46 |
| 4047 | LSS | 3.35 | 2.84 | 3.39 | 6.96 | 7.48 | 6.51 |
| 4217 | MAP3K5 | 4.42 | 3.9 | 3.55 | 9.2 | 12.81 | 13.45 |
| 4314 | MMP3 | 0.64 | 0.79 | 1.09 | 20.27 | 5.61 | 3.55 |
| 4318 | MMP9 | 794.36 | 1118.63 | 1075.21 | 3309.2 | 2232.89 | 1595.42 |
| 4598 | MVK | 3.41 | 4.5 | 5.07 | 9.46 | 9.33 | 8.48 |
| 4792 | NFKBIA | 191.16 | 198.68 | 245.55 | 531.31 | 386.63 | 405.45 |
| 4864 | NPC1 | 6.36 | 5.23 | 4.36 | 30.46 | 29.87 | 34.9 |
| 5332 | PLCB4 | 1.18 | 1.48 | 2.32 | 2.67 | 3.44 | 6.72 |
| 5335 | PLCG1 | 0.74 | 0.79 | 0.41 | 1.26 | 2.31 | 1.92 |
| 5468 | PPARG | 5.31 | 5.87 | 5.44 | 33.01 | 37.18 | 33.68 |
| 5603 | MAPK13 | 1.78 | 1.78 | 2.44 | 12.18 | 8.14 | 8.1 |
| 6300 | MAPK12 | 9.94 | 11.5 | 10.17 | 14.99 | 19.97 | 25.94 |
| 6307 | MSMO1 | 15.27 | 14.76 | 15.88 | 63.36 | 63.04 | 61.82 |
| 6309 | SC5D | 5.3 | 4.53 | 5.1 | 11.87 | 10.73 | 11.6 |
| 6348 | CCL3 | 72.98 | 77.59 | 88.5 | 2412.23 | 1030.81 | 1188.56 |
| 6349 | CCL3L1 | 13.41 | 20.75 | 48.27 | 878.83 | 311.27 | 193.91 |
| 6648 | SOD2 | 39.91 | 69.24 | 50.72 | 270.41 | 136.07 | 84.89 |
| 6713 | SQLE | 23 | 26.84 | 25.49 | 84.71 | 85.45 | 97.68 |
| 6714 | SRC | 0.28 | 0.3 | 0.84 | 2.25 | 1.4 | 1.4 |
| 6770 | STAR | 0.83 | 0.48 | 0.56 | 0.48 | 1.4 | 5.72 |
| 7097 | TLR2 | 13.85 | 13.53 | 14.67 | 64.73 | 31.88 | 25.36 |
| 7099 | TLR4 | 1.32 | 0.87 | 0.85 | 4.43 | 4.92 | 4.95 |
| 7108 | TM7SF2 | 10.31 | 12.51 | 11.06 | 22.01 | 24.03 | 22.86 |
| 7124 | TNF | 4.23 | 9.43 | 12.51 | 90.95 | 32.32 | 39.61 |
| 7480 | WNT10B | 0.28 | 0.08 | 0.33 | 1.6 | 1.05 | 1.02 |
| 8309 | ACOX2 | 0.08 | 0 | 0 | 1.7 | 1.41 | 1.29 |
| 8795 | TNFRSF10B | 4.49 | 5.71 | 5.54 | 20.35 | 13.64 | 14.96 |
| 8797 | TNFRSF10A | 12.3 | 16.3 | 12.58 | 43.6 | 60.69 | 69.33 |
| 9388 | LIPG | 0.63 | 0.73 | 0.53 | 2.59 | 1.59 | 1.68 |
| 9415 | FADS2 | 15.65 | 16.37 | 13.36 | 31.46 | 35.8 | 32.85 |
| 9451 | EIF2AK3 | 1.52 | 3.06 | 2.36 | 5.42 | 5.35 | 6.5 |
| 9619 | ABCG1 | 0.13 | 0.64 | 0.27 | 21.53 | 18.22 | 19.19 |
| 10333 | TLR6 | 12.66 | 10.53 | 12.74 | 23.08 | 21.75 | 30.56 |
| 10390 | CEPT1 | 24.08 | 20.03 | 17.34 | 30.8 | 30.54 | 56.77 |
| 10577 | NPC2 | 1304.18 | 1334.87 | 1411.43 | 3068.25 | 3254.04 | 2924.28 |
| 10682 | EBP | 46.02 | 39.5 | 41.27 | 150.66 | 136.7 | 117.76 |
| 23175 | LPIN1 | 4.49 | 2.64 | 3.04 | 6.4 | 6.32 | 7.28 |
| 23643 | LY96 | 26.77 | 24.92 | 18.78 | 97.56 | 95.7 | 85.06 |
| 29116 | MYLIP | 2.87 | 2.36 | 2.57 | 8.15 | 5.06 | 6.34 |
| 50814 | NSDHL | 11.12 | 11.38 | 10.56 | 26.15 | 30.92 | 25.51 |
| 51700 | CYB5R2 | 7.01 | 7.43 | 5.98 | 14.62 | 17.77 | 18.49 |
| 54209 | TREM2 | 32.06 | 32.24 | 35.96 | 68.74 | 70.9 | 74.69 |
| 57406 | ABHD6 | 1.34 | 1.45 | 1.52 | 3.46 | 3.81 | 3.31 |
| 64241 | ABCG8 | 0.56 | 0.7 | 0.91 | 1.74 | 1.29 | 1.5 |
| 79071 | ELOVL6 | 0.04 | 0 | 0 | 0.23 | 0.36 | 0.4 |
| 79966 | SCD5 | 1.6 | 1.22 | 1.7 | 9.88 | 8.62 | 10.72 |
| 80339 | PNPLA3 | 7.15 | 8.9 | 8.8 | 15.38 | 13.9 | 24.42 |
| 127281 | PRXL2B | 1.26 | 1.98 | 1.87 | 6.39 | 6.49 | 5.77 |
| 171546 | SPTSSA | 16 | 17.15 | 16.9 | 33 | 31.57 | 34.11 |
| 259230 | SGMS1 | 0.54 | 0.4 | 0.24 | 0.92 | 0.97 | 0.34 |
| 414062 | CCL3L3 | 60.72 | 85.49 | 54.03 | 2518.03 | 999.06 | 1338.18 |
